# Broad Shifts in Gene Expression during Early Postnatal Life Are Associated with Shifts in Histone Methylation Patterns

**DOI:** 10.1371/journal.pone.0086957

**Published:** 2014-01-28

**Authors:** Julian C. Lui, Weiping Chen, Crystal S. F. Cheung, Jeffrey Baron

**Affiliations:** 1 Section on Growth and Development, Program in Developmental Endocrinology and Genetics, Eunice Kennedy Shriver National Institute of Child Health and Human Development, National Institutes of Health, Bethesda, Maryland, United States of America; 2 Microarray Core Facility, National Institute of Diabetes and Digestive and Kidney Diseases, National Institutes of Health, Bethesda, Maryland, United States of America; Laboratoire de Biologie du Développement de Villefranche-sur-Mer, France

## Abstract

During early postnatal life, extensive changes in gene expression occur concomitantly in multiple major organs, indicating the existence of a common core developmental genetic program. This program includes hundreds of growth-promoting genes that are downregulated with age in liver, kidney, lung, and heart, and there is evidence that this component of the program drives the widespread decline in cell proliferation that occurs in juvenile life, as organs approach adult sizes. To investigate epigenetic changes that might orchestrate this program, we performed chromatin immunoprecipitation-promoter tiling array to assess temporal changes in histone H3K4 and H3K27 trimethylation (me3) at promoter regions throughout the genome in kidney and lung, comparing 1- to 4-wk-old mice. We found extensive genome-wide shifts in H3K4me3 and H3K27me3 occurring with age in both kidney and lung. The number of genes with concordant changes in the two organs was far greater than expected by chance. Temporal changes in H3K4me3 showed a strong, positive association with changes in gene expression, assessed by microarray, whereas changes in H3K27me3 showed a negative association. Gene ontology analysis indicated that shifts in specific histone methylation marks were associated with specific developmental functions. Of particular interest, genes with decreases in H3K4me3 with age in both organs were strongly implicated in cell cycle and cell proliferation functions. Taken together, the findings suggest that the common core developmental program of gene expression which occurs in multiple organs during juvenile life is associated with a common core developmental program of histone methylation. In particular, declining H3K4me3 is strongly associated with gene downregulation and occurs in the promoter regions of many growth-regulating genes, suggesting that this change in histone methylation may contribute to the component of the genetic program that drives juvenile body growth deceleration.

## Introduction

Posttranslational modifications of histone protein are thought to be important epigenetic marks closely associated with transcriptional regulation [1]. In particular, methylation of H3K4 is associated with transcriptional activation, and methylation of H3K27 with transcriptional repression [1]–[4]. Both histone methylation marks are proposed to be instrumental in gene regulation during mammalian development [4]–[6]. For example, in mouse embryonic stem cells (ESCs), genes involved in the maintenance of pluripotency like Sox2 are actively transcribed, and its promoter is marked strongly by trimethylation of H3K4 (H3K4me3) [7]. When ESCs lose pluripotency and differentiate into mouse embryonic fibroblasts (MEFs), Sox2 becomes downregulated and H3K4me3 at the Sox2 promoter is replaced by trimethylation of H3K27 (H3K27me3) [7]. Interestingly, many key developmental transcription factors with complex expression patterns are bivalently marked with both H3K4me3 and H3K27me3 in ESCs. The combination of “activation” and “repression” histone mark is usually associated with temporary silencing of the gene locus, with the gene remaining “poised” for expression once the repressive H3K27me3 mark is removed [7]–[9].

Widespread temporal changes in gene expression are not restricted to the differentiation of ESCs and embryogenesis; during early postnatal life, extensive changes in gene expression occur in most major organs. Some of these changes are organ-specific, but other shifts in gene expression occur simultaneously in multiple organs. In rodents, hundreds of growth-promoting genes are downregulated from 1- to 4-wks of age simultaneously in liver, kidney, lung, and heart. There is evidence that this multi-organ genetic program helps drive the widespread decline in cell proliferation that occurs in juvenile life, as organs approach adult size [10], [11].

We previously showed that, in a number of these growth-promoting genes, mRNA downregulation is associated with declining promoter H3K4me3 [10], suggesting a possible causal relationship. However, the genome-wide epigenetic changes during this dynamic period of early postnatal life remain largely unknown. In the current study, we combined chromatin immunoprecipitation and promoter tiling array (ChIP-on-chip) to investigate, the temporal changes of H3K4me3 and H3K27me3 at promoter regions across the genome from 1- to 4-wks of age in mouse kidney and lung *in vivo*. Gene ontology analyses were then used to explore the biological functions associated with these epigenetic changes. In addition, we combined the histone methylation analysis with our previous expression microarray analysis performed in the same organs and ages [11] to study the correlations between these epigenetic changes and regulation of gene expression.

## Materials and Methods

### Animal Procedures

All animal procedures were approved by the National Institute of Child Health and Human Development Animal Care and Use Committee. C57BL/6 male mice at 1- and 4-wk of age were purchased from Charles River and sacrificed to obtain kidney and lung tissue.

### Chromatin Immunoprecipitation (ChIP)

Cell nuclei from animal tissues were isolated as previously described [12]. Briefly, kidney and lung (50–150 mg) of C57BL/6 mice at 1 and 4 wk of age were homogenized with a Blaessig glass homogenizer on ice and then crosslinked with 1% formaldehyde for 10 min at room temperature. Pellets of cell nuclei were resuspended in SDS lysis buffer (350 µl per pellet from 50 mg of tissue) from a commercial kit (Upstate ChIP Assay Kit, Millipore, Billerica, MA) and chromatin was fragmented to approximately 500 bp by sonicating on ice for 14 cycles (10 seconds on/20 seconds off, output level 2.0) using Misonix sonicator 3000 (Thermo Fisher Scientific, Waltham, MA). 100 ng of DNA was then used for each chromatin immunoprecipitation using a ChIP Assay Kit (Millipore, Catalog number 17–295) following the manufacturer’s protocol. Rabbit polyclonal antibody against trimethyl-H3K27 (Millipore, catalog number 07–449) or trimethyl-H3K4 (Abcam Inc, catalog number ab8580) was used for immunoprecipitation. After chromatin immunoprecipitation, the chromatin crosslinking was reversed and DNA was recovered by phenol/chloroform extraction and ethanol precipitation. The DNA samples were reconstituted in deionized H_2_O. For each corresponding sample, a portion of chromatin was analyzed without chromatin immunoprecipitation (Input) to represent the unselected DNA content.

### Sequential ChIP (ChIP-reChIP)

To confirm bivalency of histone marks H3K4me3 and H3K27me3 on the same promoter, we performed sequential ChIP, using essentially the same methods as individual ChIP. Importantly, the elution after the first ChIP was performed by incubating the Protein A agarose in TE buffer with 10 mM DTT at 37°C for 30 mins, followed by a 20-fold dilution in ChIP dilution buffer (Millipore, Catalog number 20–153) [13].

### ChIP-quantitative Real-time PCR (ChIP-qPCR)

Before hybridization, the quality of the immunoprecipitated DNA was tested by real-time PCR using custom primers that amplify promoter regions previously known to show presence or absence of histone modifications being studied [10] (**Fig. S1**). Primer sequences are provided in **Table S1** in **File S1**. We also performed ChIP-qPCR validation for genes that showed changes (increases or decreases) in H3K4me3 or H3K27me3 with age, genes that showed bivalent histone modifications and genes that showed concordant declines in H3K4me3 with age. Primer sequences for these three different sets of genes are provided in **Table S2, S3, and S4** in **File S1**. The amount of histone modification at a particular position was quantified with a standard curve generated by serially diluted mouse genomic DNA. Chromatin enrichment was expressed as percent input = 100×(amount of target DNA by real-time PCR after ChIP/amount of target DNA by real-time PCR in input)/(volume of DNA solution used for ChIP/volume of DNA solution set aside as input).

### Promoter Tiling Array

DNA recovered from chromatin immunoprecipitation was amplified as previously described [14], purified, fragmented, labeled, and hybridized to Affymetrix Gene Chip Promoter 1.0R tiling array using the Affymetrix stardard ChIP-on-chip protocol. Raw data were deposited in the GEO database (accession number: GSE49785). Kidney or lung DNA from 1- or 4-wk old mice (n = 4 animals per age group) were used for chromatin immunoprecipitation using either antibodies to H3K4me3 or to H3K27me3, or IgG for input. Each microarray chip was hybridized to labeled DNA derived from a single animal.

### Bioinformatics and Statistical Analysis

Microarray signals were analyzed using the default Affymetrix RMA algorithm for tiling array. ANOVA was performed and false discovery rate (FDR) reports were generated using Partek Genome Suite 6.6 (Partek, St. Charles, MO). The Model-based Analysis of Tiling-arrays (MAT) algorithm was used to detect promoter regions enriched by immunoprecipitation with antibody against H3K4me3 or H3K27me3 [15] compared to input, using a FDR cut-off of 0.01, mean chromatin fragment size of 600 bases, and promoter definition of minus two thousand bases to plus five hundred bases of transcription start sites, based on mouse genome build 36. Venn diagrams were generated by Partek Genome Suite. Comparison of microarray signals between genes marked with H3K4me3, H3K27me3, both (bivalent), or neither (unmarked) was performed by ANOVA on Ranks. Gene ontology analysis was performed by DAVID bioinformatic resource 6.7 [16], [17]. Correlation analyses (Spearman’s Rank Correlation) and chi-square tests were performed by SigmaPlot 11.0 (Systat Software, San Jose, CA). Comparison of mRNA expression and histone H3K4me3 with age using real-time PCR was performed by one-way-ANOVA. For ChIP-reChIP, two-way-ANOVA was used with two independent variables: genomic position, and the antibody used in the reChIP (H3K27me3 versus IgG). Known and predicted protein-protein interactions were analyzed by STRING9.0 (string-db.org).

### Defining Temporal changes in Histone Modifications from 1- to 4-wk

We combined the tiling microarray data for the two age groups to compare the histone modifications between 1- and 4-wk of age in the same organ. We considered genes that showed significant H3K4me3 signals at 1-wk (versus Input DNA) but not at 4-wk to have “lost” H3K4me3 with age. On the contrary, genes that showed significant H3K4me3 signals only at 4-wk old were considered to have “acquired” the mark with age. For genes with significant histone methylation at both 1- and 4-wk compared to input DNA, the quantitative level of that histone methylation may have changed substantially with age, which might lead to altered gene expression. To detect these quantitative temporal changes in histone methylation, all genes in the microarray that showed histone signal at both 1- and 4-wk were ranked by the change in MAT score [15] with age for a particular histone mark, and then divided into three groups: lower quartile (0–25^th^), middle quartiles (25^th^–75^th^), and upper quartile (75^th^–100^th^). The lower quartile consisted of genes that showed decreasing MAT score, which represents decreasing histone methylation with age. On the contrary, the upper quartile consisted of genes that showed increasing MAT score and therefore increasing histone methylation with age. The middle quartiles comprised genes that showed little change in histone methylation with age. This approach of categorizing genes into three groups (decreased, increased, and unchanged) was used because there is currently no clear understanding about what cut-off value for change in MAT score should be used identify biologically meaningful changes in histone methylation [15].

### Quantitative Real-time PCR

Real-time PCR was used to assess specific mRNA levels in mouse kidney and lung. Organs were dissected from 1- and 4-wk old C57BL/6 mice (n = 5 animals per time point). Total RNA (100–200 ng) was reverse transcribed using SuperScript III Reverse Transcriptase (Invitrogen). Quantitative real-time PCR was performed for 18S, Aurkb, Ccnd2, Sox11, Rrm2, and Zwilch using commercially available FAM or VIC-labeled Taqman assays (Applied Biosystems, Foster City, CA). Reactions were performed in triplicate on cDNA derived from each animal using the ABI prism 7900 Sequence Detection System instrument (Applied Biosystems). The relative quantity of each mRNA was calculated using the formula: Relative Expression = 2^–ΔCt^×10^6^, where Ct represents the threshold cycle and ΔCt = (Ct of gene of interest)–(Ct of 18S rRNA). Values were multiplied by 10^6^ for convenience of comparison.

## Results

### H3K4me3 is Associated with Higher Expression and H3K27me3 is Associated with Lower Expression Levels

We performed chromatin immunoprecipitation (ChIP) with genomic DNA isolated from normal mouse kidney and lung at 1- and 4-wk of age using antibodies that recognize specific histone modifications H3K4me3 or H3K27me3. The ChIP-enriched DNA and total genomic DNA (Input) were hybridized to tiling microarrays that interrogate over 25,500 mouse promoters (Affymetrix Gene Chip Promoter 1.0R). Promoter regions with histone binding was identified by significant enrichment in the immunoprecipitated DNA compared to the Input DNA (ANOVA, FDR<0.01). Since we were primarily interested in the correlation between histone modifications and gene expression, we combined the promoter analysis with our previous expression microarray [11] (1- and 4-wk old C57BL/6 mice, kidney and lung, n = 5 in each group), and all subsequent analysis presented in this study focused on the 21,679 genes for which we had both promoter histone modification and gene expression levels.

**Figure 1:**
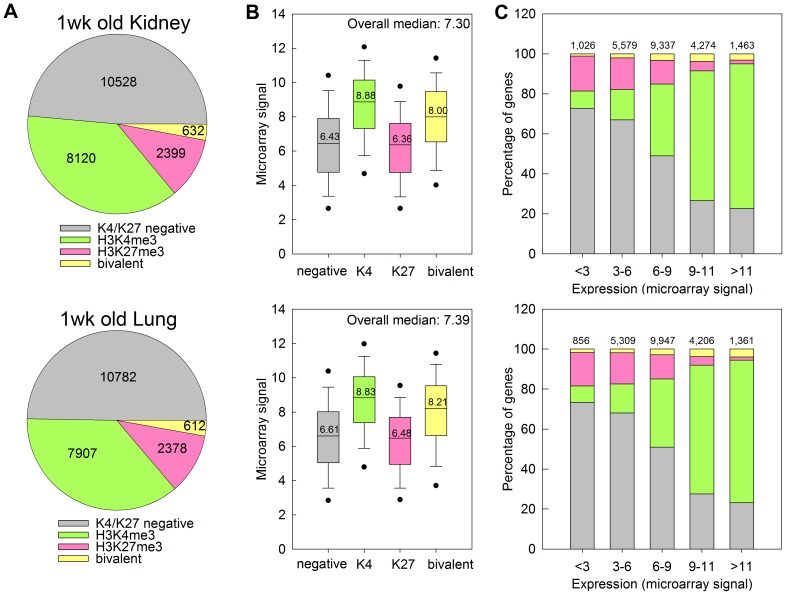
H3K4me3 is associated with high levels of gene expression and H3K27me3 is associated with low levels of gene expression in kidney and lung of 1-wk old mice. (A) Pie chart depicting the distribution of H3K4me3 and H3K27me3 marks at promoter regions of genes across the genome, in kidney (top) and lung (bottom) of 1-wk old mice. The number of genes within each category are indicated. (B) Box and whisker plot showing the expression microarray signals of genes with different types of histone methylation. The line within each box represents the median microarray signal of the genes in the designated gene set, with the median value displayed over the line. The upper and lower boundaries of the box indicate the 75^th^ and 25^th^ percentiles, respectively. Whiskers (error bars) above and below the box indicate the 90^th^ and 10^th^ percentiles and outlying dots indicate the 95^th^ and 5^th^ percentiles. The overall median of the microarray signal from the whole genome is displayed on the upper right corner of each graph. (C) Bar graphs showing the distribution of H3K4me3 and H3K27me3 marks in gene sets with different levels of expression. H3K4me3 marks occurred more frequently in genes with higher expression levels, and H3K27me3 marks occurred more frequently in genes with lower expression levels. The color code for all three panels is shown in panel A. Numbers at the top of bars represent total number of genes in each group.

Tiling array analysis of 1-wk old kidney and lung suggested that the distribution of H3K4me3 and H3K27me3 marks across the genome is very similar between the two organs (**Fig. 1A**). About half of all promoters did not show a significant H3K4me3 or H3K27me3 mark, 35–40% of all promoters were H3K4me3 positive, 10–13% were H3K27me3 positive, and about 2% were positive for both marks. In a subset of genes that showed both histone marks, we confirmed bivalency using ChIP-reChIP (**Fig. S2**).

Expression analysis of genes associated with these promoters suggested that H3K4me3 is generally associated with higher levels of expression (**Fig. 1B**). For example in 1-wk old kidney, genes marked with H3K4me3 showed a significantly higher average level of expression than genes unmarked (8.88 versus 6.43, *P*<0.05, ANOVA on ranks). H3K27 marked genes showed a modest, yet statistically significant association with lower expression levels compared to unmarked (6.36 versus 6.43, *P*<0.05). Genes marked by both H3K4me3 and H3K27me3 showed an elevated level of expression compared to unmarked genes (8.00 versus 6.43, *P*<0.05), which agreed with the observation that H3K4me3 has a stronger association with gene expression than H3K27me3. Similarly, when we divide the genes into groups with different expression levels (**Fig. 1C**), genes with the lowest expression (microarray signal <3) were predominantly negative for both H3K4me3 and H3K27me3, or only H3K27me3 positive. In groups with progressively higher expression levels, the proportion of genes with negative for both marks or positive only for H3K27me3 decreased, and genes with H3K4me3 or bivalent marks progressively increased. In the group with the highest level of expression (microarray signal >11), the large majority of genes were H3K4me3 positive (**Fig. 1C**), supporting the idea that H3K4me3 is associated with active transcription.

### Bivalently Marked Genes are Enriched for General Developmental Functions

We used the DAVID 2008 web-server [16] to detect enriched gene ontology (GO) terms for genes marked with different histone methylation marks. In 1wk old kidney and lung, genes with bivalent marks were significantly enriched for developmental functions (**Table S5–6** in **File S1**). For example, the three most significant biological functions in genes with bivalent marks in 1-wk old kidney were tube development (GO:0035295, FDR = 1.89e^−13^), urogenital system development (GO:0001655, FDR = 9.53e^−11^), and kidney development (GO:0001822, FDR = 1.17e^−8^). Similarly, in 1-wk old lung, epithelium development (GO:0060429, FDR = 7.67e^−11^), lung development (GO:0030324, FDR = 8.40e^−10^) and respiratory tube development (GO:0030323, FDR = 1.25e^−9^) ranked among the most significant GO terms.

### H3K4me3 Marked Genes are Enriched for Cell Cycle Function

Gene ontology analysis also indicated that H3K4me3-positive genes at 1-wk of age showed a wide range of biological functions, which were generally similar between kidney and lung (**Table S7–8** in **File S1**). Some of the major categories that showed the strongest enrichment included protein localization/transportation (GO:0045184, GO:0008104, GO:0015031, GO:0046907) and catabolic processes (GO:0009057, GO:0044265, GO:0030163). Interestingly, cell division functions contributed to more than 10 of the 50 most significant GO terms, perhaps reflecting the active transcription of cell cycle-related genes during this juvenile period, when the organs are rapidly growing and many cells are still undergoing active proliferation.

### H3K27me3 Marked Genes are Enriched for Neuronal Differentiation and Development

Similar to H3K4me3, H3K27me3 positive genes are implicated in a variety of biological functions, among which neuron differentiation constitute an overwhelming proportion (**Table S9–10** in **File S1**). The expression of these genes is important for neurodevelopment, and therefore should presumably be actively transcribed in neuronal cells, but remain silenced in other cell types. It is therefore not surprising that in kidney and lung, H3K27me3 mark is present at the promoter regions for silencing of these genes.

### Gene Ontology Analysis of Histone Methylation Patterns at 4wk of Age Implicates Functions Similar to those at 1 wk

We next analyzed the tiling promoter microarray for kidney and lung at 4 wk of age. The overall distribution of H3K4me3 and H3K27me3 marks across the genome at 4 wk of age (**Fig. S3**, left panel) was similar to that observed at 1-wk of age. As at 1 week, H3K4me3 at 4 weeks of age was associated with higher levels of expression and H3K27me3 was associated with lower levels of expression (**Fig. S3**, middle and right panel). Gene ontology analyses of genes marked with H3K4me3 and/or H3K27me3 at 4-wk of age yielded results (**Table S11–16** in **File S1**) similar to those of 1-wk old mice, except that in the 4-wk old kidney, bivalent genes were not strongly implicated in developmental functions, but rather involved in tissue homeostasis (**Table S9** in **File S1**).

### Temporal changes in Histone Modifications from 1- to 4-wk Kidney and Lung

**Figure 2:**
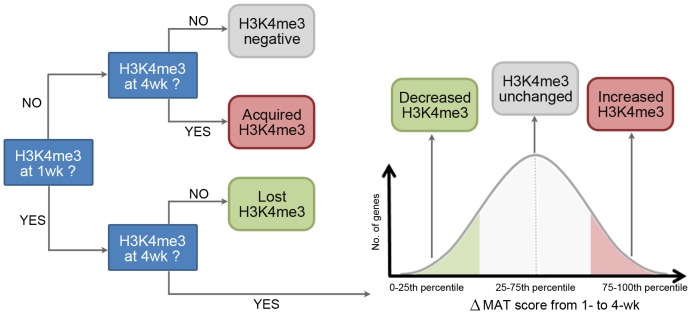
Schematic diagram showing the categorization of genes based on change in histone methylation with age. Compared to Input DNA, genes with no significant H3K4me3 signal at both 1- and 4-wk were considered H3K4me3 negative. Genes with signal at only 1-wk but not 4-wk were considered to have “lost” H3K4me3 with age, and genes with signal at only 4-wk but not 1-wk were considered to have “acquired” H3K4me3. Genes with significant H3K4me3 signals at both 1- and 4-wk were ranked by the change in MAT score with age and divided into lower quartile (0–25^th^), middle quartiles (25^th^–75^th^), and upper quartile (75^th^–100^th^). Genes in the lower, middle, and upper quartile were considered as showing decreased H3K4me3, unchanged H3K4me3, and increased H3K4me3 with age, respectively. H3K27me3 was analyzed analogously.

To gain insight into how changes in histone modifications regulate biological functions from 1- to 4-wk, we combined the tiling microarray data for the two age groups and focused on genes that showed changes in histone methylation with age. As described in the materials and methods section and summarized in **Fig. 2**, genes that showed significant histone mark only at 1- or only at 4-wk were considered to have “lost” or “acquired” the mark with age, respectively. Based on this definition, the vast majority of genes retained their histone marks with age (**Table 1**), except for bivalent genes, of which only 50–60% remained bivalent from 1- to 4-wk. We also categorized all genes that had significant histone methylation at both 1- and 4-wk based on their change in MAT score [15] with age for a particular histone mark into three groups: lower quartile (0–25^th^), middle quartiles (25^th^–75^th^), and upper quartile (75^th^–100^th^). The lower quartile consisted of genes that showed decreasing MAT score and therefore decreasing histone methylation with age, while the upper quartile consisted of genes with increasing MAT score and increasing histone methylation with age. The middle quartiles comprised genes that showed little change in histone methylation with age (**Fig. S4**). We used ChIP-qPCR in a subset of genes to confirm the validity of our approach for identifying changes in histone modifications from 1- to 4-wk organs (**Fig. S5**).

**Table 1:** Genome wide changes of histone H3K4me3 and H3K27me3 marks from 1 to 4 wk kidney and lung.

		Histone Marks at 4 wk								
	Kidney	H3K4me3	H3K27me3			Bivalent	Negative for both		Total	
**Histone Marks at 1 wk**	**H3K4me3**	7759	49			84	228		8120	
	**H3K27me3**	65	2109			89	193		2399	
	**Bivalent**	190	131			303	8		632	
	**Negative for both**	677	437			14	9400		10528	
		**Histone Marks at 4 wk**								
	**Lung**	**H3K4me3**		**H3K27me3**	**Bivalent**			**Negative for both**		**Total**
**Histone Marks at 1 wk**	**H3K4me3**	6995		61	155			696		7907
	**H3K27me3**	10		2203	57			108		2378
	**Bivalent**	66		158	376			12		612
	**Negative for both**	241		467	14			10060		10782

Genes were categorized by their histone marks at 1- and 4-wk of age. The vast majority of genes retained their histone marks with age except for bivalent genes, of which only 50–60% remained bivalent from 1-wk to 4-wk.

### Temporal changes in Histone Methylation during Early Postnatal Life are Associated with changes in Gene Expression

We next compared the temporal changes in histone methylation with the temporal changes in gene expression from 1 to 4 wk of age. Changes in gene expression from 1 to 4-wk were positively correlated with changes in H3K4me3 (**Fig. S6**, Spearman’s p = 0.137, kidney; p = 0.111, lung; both P<2e-08) and negatively correlated with changes in H3K27me3 (p = −0.060, kidney; p = −0.041, lung; both P<2e-08). In a similar analysis, the set of age-downregulated genes (≥2-fold, FDR<0.05) was significantly enriched with genes showing loss of or decrease in H3K4me3, and with genes showing acquisition of or increase in H3K27me3 (**Table 2**, **3**, P<0.001 in both kidney and lung, Pearson’s chi-square test). Conversely, the set of age-upregulated genes (≥2-fold, FDR<0.05) was significantly enriched with genes showing acquisition of or increase in H3K4me3, and with genes showing loss of or decrease in H3K27me3 (**Table 2**, **3**, P<0.001 in both organs).

**Table 2:** Temporal changes of H3K4me3 is associated with changes of gene expression in kidney and lung.

	No. of genes			
Kidney	Lost/decreasedH3K4me3	H3K4me3unchanged	Acquired/increasedH3K4me3	Chi-square P-value
**Whole Genome**	2500 (26.0%)	4168 (43.4%)	2929 (30.5%)	
**Age-downregulated (≥2-fold, FDR<5%)**	381 (50.8%)	278 (37.1%)	91 (12.1%)	P<0.001
**Age-upregulated (≥2-fold, FDR<5%)**	86 (11.8%)	216 (29.5%)	429 (58.7%)	P<0.001
**Expression not significantly changed**	2033 (25.0%)	3674 (45.3%)	2409 (29.7%)	P = 0.047
	**No. of genes**			
**Lung**	**Lost/decreased H3K4me3**	**H3K4me3 unchanged**	**Acquired/increased H3K4me3**	**Chi-square P-value**
**Whole Genome**	2825 (32.0%)	3796 (42.9%)	2220 (25.1%)	
**Age-downregulated (≥2-fold, FDR<5%)**	314 (57.0%)	177 (32.1%)	60 (10.9%)	P<0.001
**Age-upregulated (≥2-fold, FDR<5%)**	88 (17.9%)	164 (33.3%)	240 (48.8%)	P<0.001
**Expression not significantly changed**	2423 (31.1%)	3455 (44.3%)	1920 (24.6%)	P = 0.202

Genes were categorized by changes in gene expression with age (rows) and changes in histone H3K4me3 with age (columns). In both kidney and lung, age-downregulated genes (≥2-fold, FDR<0.05) were significantly enriched with genes showing loss of or decrease in H3K4me3, Conversely, the set of age-upregulated genes (≥2-fold, FDR<0.05) was significantly enriched with genes showing acquisition of or increase in H3K4me3.

**Table 3:** Temporal changes of H3K27me3 is associated with changes of gene expression in kidney and lung.

	No. of genes			
Kidney	Lost/decreasedH3K27me3	H3K27me3unchanged	Acquired/increasedH3K27me3	Chi-square P-value
**Whole Genome**	1100 (30.4%)	1287 (35.6%)	1228 (34.0%)	
**Age-downregulated (≥2-fold, FDR<5%)**	65 (18.3%)	127 (35.7%)	164 (46.1%)	P<0.001
**Age-upregulated (≥2-fold, FDR<5%)**	117 (57.1%)	58 (28.3%)	30 (14.6%)	P<0.001
**Expression not significantly changed**	918 (30.1%)	1102 (36.1%)	1034 (33.9%)	P = 0.910
	**No. of genes**			
**Lung**	**Lost/decreased H3K27me3**	**H3K27me3 unchanged**	**Acquired/increased H3K27me3**	**Chi-square P-value**
**Whole Genome**	895 (24.3%)	1397 (37.9%)	1395 (37.8%)	
**Age-downregulated (≥2-fold, FDR<5%)**	38 (13.7%)	87 (31.4%)	152 (54.9%)	P<0.001
**Age-upregulated (≥2-fold, FDR<5%)**	94 (34.3%)	96 (35.0%)	84 (30.7%)	P<0.001
**Expression not significantly changed**	763 (24.3%)	1214 (38.7%)	1159 (37.0%)	P = 0.723

Genes were categorized by changes in gene expression with age (rows) and changes in histone H3K27me3 with age (columns). In both kidney and lung, age-downregulated genes (≥2-fold, FDR<0.05) was significantly enriched with genes showing acquisition of or increase in H3K27me3. Conversely, the set of age-upregulated genes (≥2-fold, FDR<0.05) was significantly enriched with genes showing loss of or decrease in H3K27me3.

### Gene Ontology Analyses of Genes that showed Temporal changes in Histone Methylation

**Figure 3:**
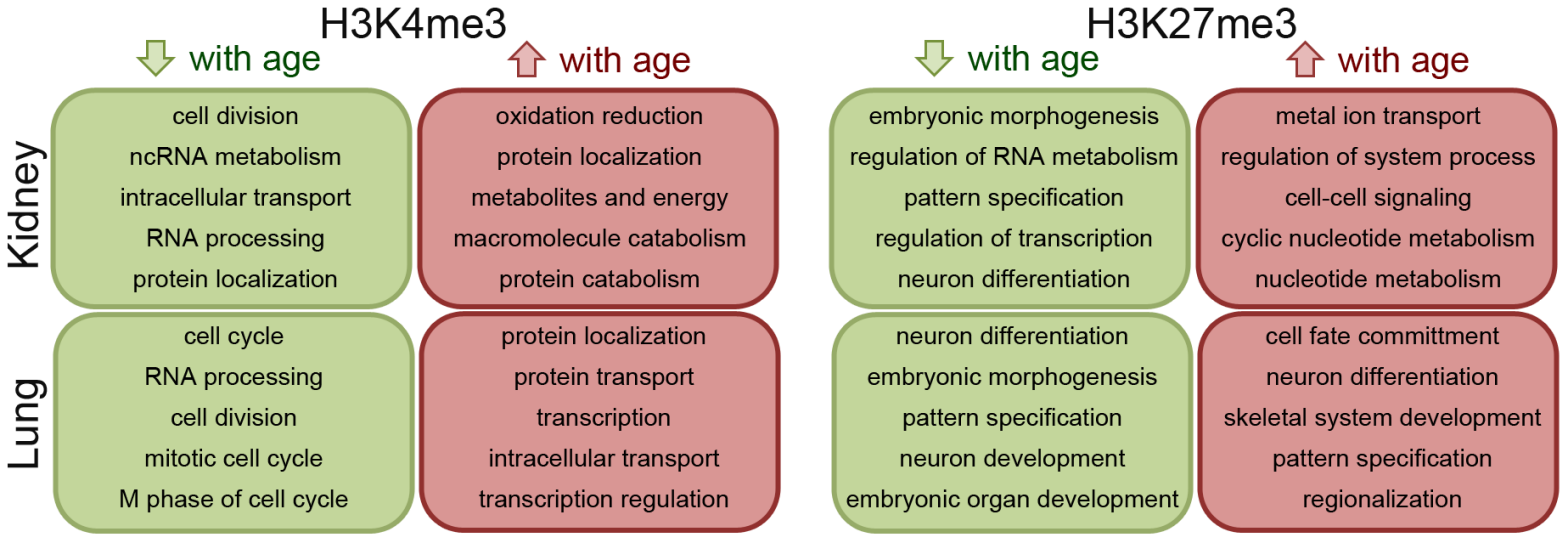
Schematic diagram summarizing the gene ontology analysis results for the genes that showed increasing and decreasing H3K4me3 and H3K27me3 with age in kidney and lung. Only the top 5 gene ontology terms were shown in each organ and histone mark.

Gene ontology analysis (**Fig. 3**) suggested that cell division and cell cycle-related functions were strongly enriched in the genes that showed loss of or decrease in H3K4me3 marks with age, in both kidney and lung (**Table S17–18** in **File S1**). For genes that acquired or showed increased H3K4me3 with age, major implicated biological functions included protein localization/transport (both organs) and catabolism and apoptosis (in kidney) (**Table S19–20** in **File S1**). Genes that lost or showed decreasing H3K27me3 with age in both kidney and lung were predominantly implicated in morphogenesis and developmental functions (**Table S21–22** in **File S1**). Genes that acquired or showed increasing H3K27me3 with age in kidney and lung were also enriched with developmental functions, along with ion transportation and cell motion/migration (**Table S23–24** in **File S1**).

### Concordant changes in Histone Methylation with Age between Kidney and Lung

**Figure 4:**
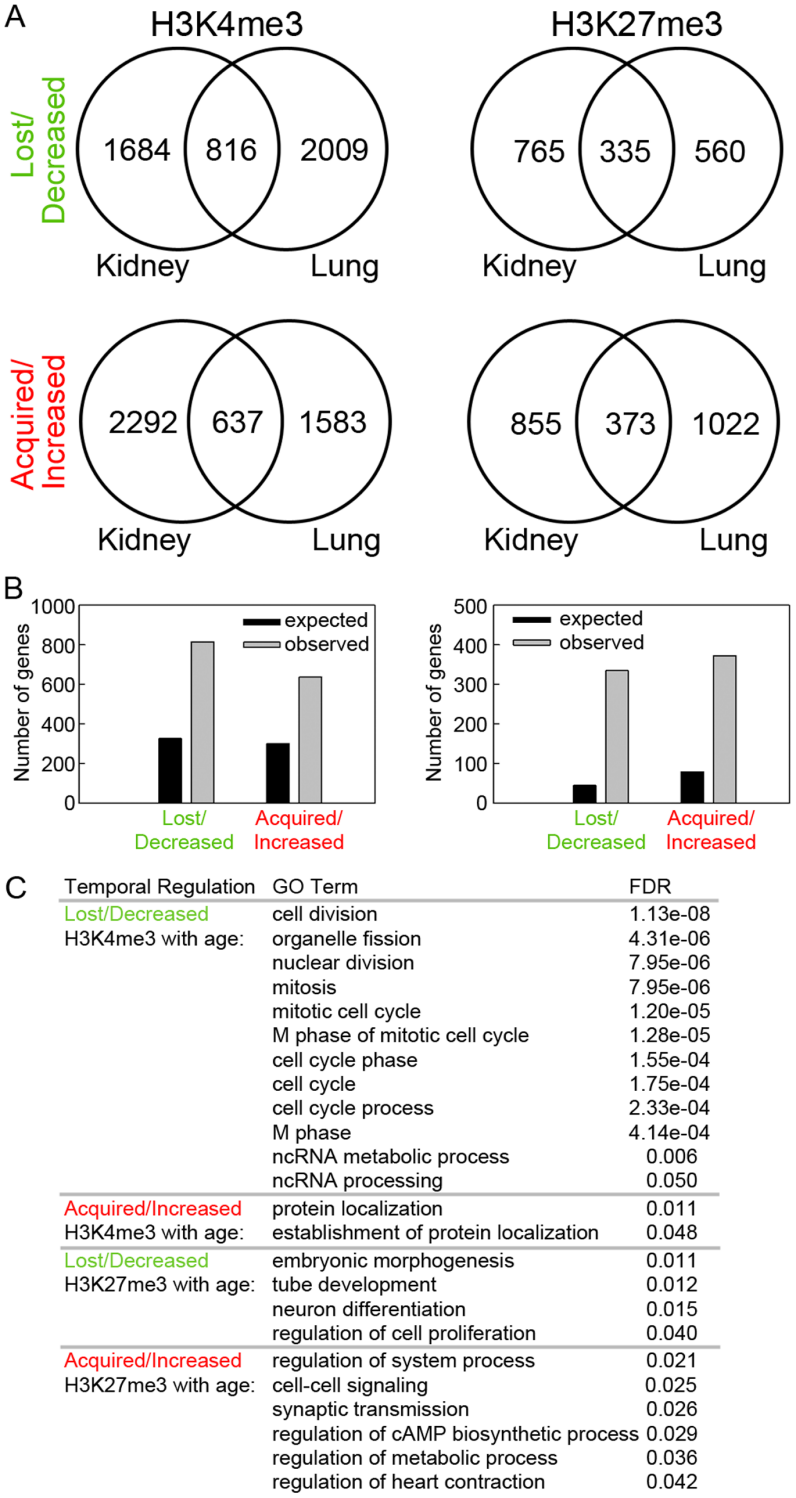
Changes in histone methylation with age show significant overlap in kidney and lung. (**A**) Venn diagrams depicting the number of genes that showed loss of or decrease in H3K4me3 (upper left) or H3K27me4 (upper right) with age overlapped significantly between kidney and lung. Similarly, the number of genes that showed acquisition of or increase in H3K4me3 (bottom left) or H3K27me3 (bottom right) with age overlapped significantly between kidney and lung. (**B**) Observed and expected overlap of genes that showed changes in H3K4me3 (left) or H3K27me3 (right) in the same direction in both kidney and lung. Black bars, expected; grey bars, observed. The observed overlap between organs was significantly greater than the overlap expected by chance (Pearson’s Chi-square test, all P<0.001). (**C**) Gene ontology analyses (DAVID bioinformatic resource 6.7) of genes that showed concordant changes in histone methylation in kidney and lung. All GO Terms with a false discover rate (FDR) of <0.05 were listed. Genes with a concordant decrease in H3K4me3 with age were significantly enriched with cell division and cell cycle functions.

**Figure 5:**
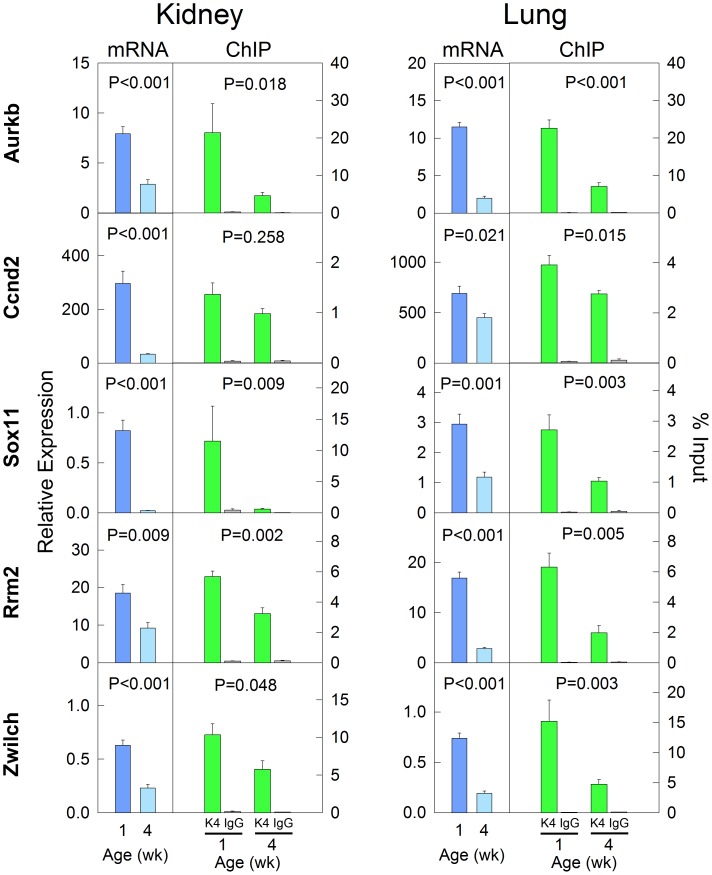
Validation of decreased expression and H3K4me3 using RT-qPCR and ChIP-qPCR. A subset of genes showing concordant mRNA downregulation and decreasing H3K4me3 with age in both kidney and lung was studied. The relative expression of each mRNA was measured by real-time PCR and normalized to 18S RNA. For ChIP, chromatin was immunoprecipitated with antibodies to H3K4me3. Real-time PCR was then used to measure content of indicated genomic regions in the immunoprecipitated DNA compared to input DNA. P values (ANOVA) are for change in mRNA level or H3K4me3 level with age.

**Figure 6:**
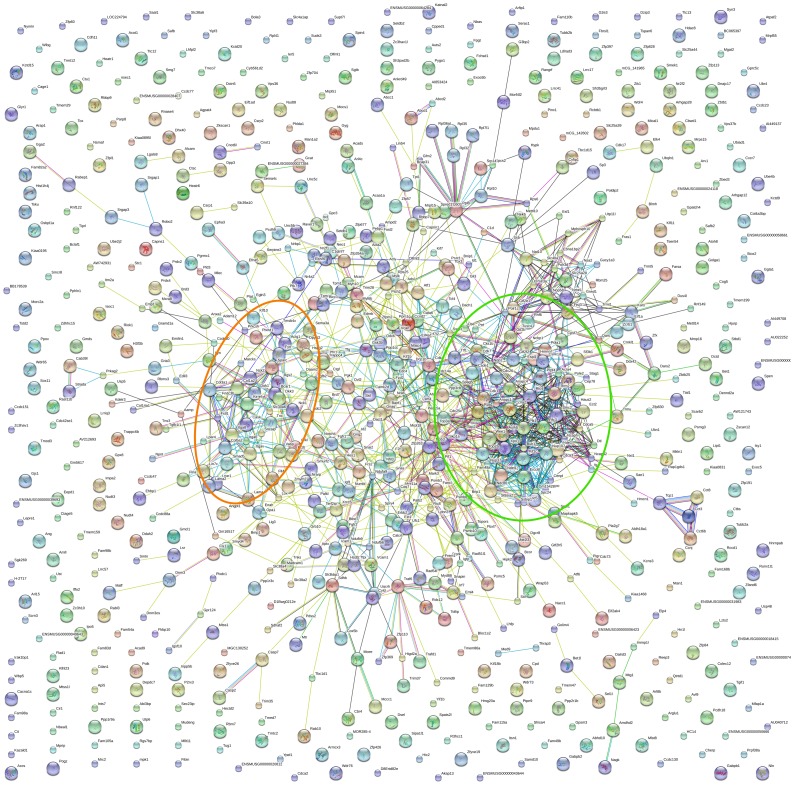
Protein interactions of genes that showed concordant loss of or decrease in H3K4me3 with age in both kidney and lung. STRING9.0 was used for analysis of protein interactions. The two biggest clusters consist of many proteins that are involved in cell cycle functions (circled in green), and extracellular matrix proteins (circled in orange).

We next analyzed the overlap in histone methylation changes with age between different organs. We found that the number of genes that showed concordant changes in histone methylation in both kidney and lung was much greater than would be expected by chance if regulation in the two organs were independent events (**Fig. 4A–B**, P<0.001, chi-square test). Gene ontology analysis showed that the genes with concordant lost/decreased H3K4me3 with age are strongly implicated in cell division and cell cycle functions (**Fig. 4C**). This is consistent with the gene ontology analysis performed in individual organs, and also with our previous findings that genes concordantly downregulated with age in multiple organs are primarily involved in cell growth and proliferation [10], [11]. The downregulation of mRNA and H3K4me3 with age in a subset of genes was confirmed using real-time PCR and ChIP-qPCR, respectively (**Fig. 5**) On the other hand, genes with concordant increases in H3K4me3 or concordant changes (increase or decrease) of H3K27me3 did not point strongly to a particular group of biological functions. This finding is supported by a different bioinformatic analysis based on protein-protein interactions (STRING 9.0). Proteins encoded by the genes that showed a concordant decrease in H3K4me3 in both organs formed several clusters of extensive protein-protein interactions (**Fig. 6**). The biggest cluster consisted of many proteins that are involved in cell cycle functions, including cyclins (*Ccna2, Ccnd2*), cyclin-dependent kinases (*Cdk1, Cdk4, Cdk7, Cks1b*) and condensins (*Smc2, Ncapd2, Ncaph*). The second biggest protein cluster, which consists of multiple ECM proteins (*Col1a2, Col3a1, Col5a2*) and integrins (*Itga6, Itga8, Itgav*), involves cell adhesion and cell signaling. In contrast, proteins encoded by genes with concordant increases in H3K4me3 or concordant changes (increases or decreases) in H3K27me3 only showed sporadic interactions, rather than forming an extensive network (**Fig. S7**). Taken together, these analyses suggest that the concordant downregulation of H3K4me3 in cell-cycle genes may be implicated in the decline of cell proliferation with age in multiple organs during early postnatal life.

## Discussion

Broad shifts in gene expression have been identified during differentiation of specific isolated cell types and during embryogenesis [18]–[21]. Far less, however, is known about the changes in gene expression that occur during postnatal development. We recently demonstrated in mice and rats that, during juvenile life, extensive temporal changes in gene expression occur in most major organs [10], [11]. Interestingly these postnatal developmental shifts in gene expression are strikingly similar in different organs, indicating the existence of a common core developmental genetic program that occurs during early postnatal life. This program includes hundreds of growth-promoting genes that are downregulated from 1- to 4-wks of age simultaneously in liver, kidney, lung, and heart, and there is evidence that this component of the program helps drive the widespread decline in cell proliferation that occurs in juvenile life, as organs approach adult size [10], [11]. The molecular mechanisms that orchestrate these extensive changes in gene expression occurring in multiple postnatal organs are not known. Here we found that this multi-organ juvenile genetic program is associated with global shifts in histone methylation.

To explore postnatal changes in histone methylation patterns, we assessed the patterns of H3K4me3 and H3K27me3 at promoter regions across the genome in kidney and lung of 1- and 4-wk-old mice and compared these genome-wide patterns of histone methylation to genome-wide patterns of gene expression. We found that, in both organs and at both ages, the histone mark H3K4me3 was associated with high levels of gene expression, and H3K27me3 was associated with low levels of expression. These associations are similar to associations identified in other biological systems, including human embryonic stem cells differentiation [22]–[24], erythroid cells development [8], thyroid hormone (T3)-dependent metamorphosis [25], and dendritic cells activation [26]. Our findings suggest that this general concept – that H3K4me3 is associated with active transcription and H3K27me3 is associated with gene silencing – applies also to postnatal tissues *in vivo*. Similarly, we found that temporal changes in histone methylation in juvenile animals were associated with temporal changes in gene expression. In both mouse kidney and lung, from 1- to 4-wks of age, loss of H3K4me3 and acquisition of H3K27me3 were strongly associated with downregulation of gene expression, and conversely, acquisition of H3K4me3 and loss of H3K27me3 were associated with upregulation of gene expression. Although these strong temporal associations are consistent with the hypothesis that histone methylation helps coordinate the widespread changes in gene expression occurring during early postnatal life, the actual causal relationship between histone methylation marks and gene expression remain unclear, not only in this setting but in general [26]–[29]. The observed changes in histone H3K4 and H3K27 methylation could arise from a variety of possible mechanisms. The level of methylation depends on the opposing activities of histone methyltransferases, (such as Set1/MLL complexes for H3K4 and EZH1/2 for H3K27) and histone demethylases (such as KDM family members) [30]. In addition, chromatin remodelers can affect histone methylation levels, for example, by nucleosome ejection [31].

Interestingly, the genome-wide pattern of H3K4 and H3K27 trimethylation showed strong similarity in kidney and lung, at both ages studied. Furthermore, the shifts in both K4 and K27 methylation patterns between 1- and 4-wks of age showed marked similarity in the two organs studied, suggesting that there might be a common core developmental program of histone methylation occurring simultaneously in multiple organs, which accompanies the common core developmental program of gene expression that occurs in multiple organs. Studies of additional organs are needed to determine how widespread this histone methylation program is among other tissues.

Gene ontology analysis was used to explore the possibility that specific changes in histone methylation might be associated with discrete biological functions. An interesting pattern was observed for the genes marked with both H3K4me3 and H3K27me3. At 1-wk of age, this apparent bivalent histone mark was strongly enriched for genes with developmental functions, which is consistent with previous reports showing that many key developmental genes are bivalently marked in embryonic stem cells [7], [9]. At 4-wks of age, this apparent bivalent mark was predominantly found in genes involved in tissue homeostasis, suggesting that, as postnatal development reaches completion in the adult, the bivalent mark shifts from genes actively expressed for developmental functions to genes actively expressed for tissue maintenance functions. The presence of both marks does not prove that individual promoters are associated with both histone modifications, but could arise from the fact that we studied whole organs which comprise multiple cells types. Thus, a promoter region might be associated with H3K4me3 in some cell types but with H3K27me3 in other cell types. We therefore confirmed bivalency in a subset of genes with both histone marks using ChIP-reChIP (**Fig. S2**). Other genes that were not evaluated by ChIP-reChIP may not be truly bivalent.

Our findings also point to the potential involvement of H3K4me3 in the regulation of cell proliferation. Gene ontology analyses of genes marked with H3K4me3 were enriched for cell-cycle-related functions in both 1- and 4-wk-old kidney and lung. Similarly, in both kidney and lung, genes that showed decreasing H3K4me3 marks from 1- to 4-wk were strongly enriched for cell division and cell cycle-related functions. Furthermore, when the analysis was focused on genes that showed concordant changes in histone methylation in both kidney and lung, those genes with declining H3K4me3 in both organs were particularly enriched for cell division and cell cycle-related functions. Thus, the findings suggest that, during this early postnatal period, decreasing H3K4me3 is associated with downregulation of genes involved in cell-cycle functions. Because the downregulation of growth-regulating genes appears to be involved in the physiological postnatal slowing of body growth, the combined evidence suggests declining H3K4me3 is also involved in postnatal growth deceleration.

In summary, our combined ChIP-on-chip and expression array analysis in the mouse suggests that extensive genome-wide shifts in H3K4me3 and H3K27me3 are occurring during early postnatal life, and that these changes in histone methylation are strongly associated with genome-wide changes in gene expression occurring concordantly in multiple organs. First, in postnatal tissues, we observed a strong association between histone methylation marks and gene expression and also an association between temporal changes in histone methylation marks and changes in gene expression during juvenile life. Second, we found substantial similarities between histone methylation patterns in two different organs and similarities between temporal changes in histone methylation in these organs, raising the possibility that there may exist a common developmental program of histone methylation occurring simultaneously in multiple organs. Third, using gene ontology analysis, we found that shifts in specific histone methylation marks were associated with specific developmental functions. Of particular interest, genes showing declining H3K4me3 were strongly enriched for functions related to cell proliferation.

Taken together, the findings indicate that the common core developmental program of gene expression which occurs in multiple organs during juvenile life is associated with a common core developmental program of histone methylation. The findings also suggest that one component of this program, declining H3K4me3, is associated with the declining expression of multiple growth-regulating genes. Prior evidence suggests that this declining expression contributes to the physiological slowing of proliferation that occurs in multiple tissues during juvenile life. Therefore, the current findings suggest that declining H3K4me3 may contribute to the developmental program that limits somatic growth in mammals.

## Supporting Information

Figure S1 **Assessment of DNA quality after chromatin immunoprecipitation.** Chromatin from kidney of 1-wk and 4-wk old mice was immunoprecipitated with antibodies to H3K4me3 **(A)** or H3K27me3 **(B).** Real-time PCR was used to measure content of indicated genomic regions in the immunoprecipitated DNA compared to input DNA. *Igf2* and *Inmt* served as positive controls for H3K4me3 at 1- and 4-wk, respectively. Chr19∶23.12 Mb was a negative control for H3K4me3. *Mdk* intronic region was a positive control for H3K27me3, and Chr10∶79.15 Mb was a negative control for H3K27me3.
(TIF)

Figure S2 **Validation of H3K4me3 and H3K27me3 bivalency using ChIP-reChIP.** Chromatin from kidney and lung of 1-wk old mice was immunoprecipitated first with antibodies to H3K4me3, followed by DNA purification (green bar), or a second immunoprecipitation using antibodies to H3K27me3 (red bar) or IgG (yellow bar). Real-time PCR was then used to measure content of indicated genomic regions in the immunoprecipitated DNA compared to input DNA. Chr19∶23.12 Mb and Chr10∶79.15 Mb was used as negative control for both H3K4me3 and H3K27me3. *Ezh2* was a positive control for H3K4me3 but negative control for H3K27me3. Two-way-ANOVA was performed using genomic position and effect of antibody in the re-ChIP (H3K27me3 versus IgG) as the two independent variables. P values represent the effect of antibody in the re-ChIP. Number of animals = 5 in each data point.
(TIF)

Figure S3 **H3K4me3 is associated with high level of gene expression and H3K27me3 is associated with low level of gene expression in kidney and lung of 4-wk-old mice.** Pie chart (left) depict the distribution of H3K4me3 and H3K27me3 marks at promoter regions of genes across the genome, in kidney (top) and lung (bottom) of 4-wk old mice. The number of genes within each category are indicated. Box and whisker plots (center) show the expression microarray signals of genes with different types of histone methylation. The line within each box represents the median microarray signal of the genes in the designated gene set, with the median value displayed over the line. The upper and lower boundaries of the box indicate the 75^th^ and 25^th^ percentiles, respectively. Whiskers (error bars) above and below the box indicate the 90^th^ and 10^th^ percentiles and outlying dots indicate the 95^th^ and 5^th^ percentiles. The overall median of the microarray signal from the whole genome is displayed on the upper right corner of each graph. Bar graphs (right) show the distribution of H3K4me3 and H3K27me3 marks in gene sets with different levels of expression. H3K4me3 marks occurred more frequently in genes with higher expression levels, and H3K27me3 marks occurred more frequently in genes with lower expression levels. The values above bars indicate number of genes in each group. The color code for all graphs is shown in below pie charts.
(TIF)

Figure S4 **Box and whisker plot showing the change in MAT scores of gene sets as categorized in** **Fig. 2** **.** The line within each box represents the median MAT score of the genes in the designated gene set. The upper and lower boundary of the box indicates the 75^th^ and 25^th^ percentiles, respectively. Whiskers (error bars) above and below the box indicate the 90^th^ and 10^th^ percentiles and outlying dots indicate the 95^th^ and 5^th^ percentiles.
(TIF)

Figure S5 **Validation of changes of H3K4me3 and H3K27me3 with age using ChIP-qPCR.** Chromatin from lung (top panels) or kidney (bottom panels) of 1-wk and 4-wk old mice was immunoprecipitated with antibodies to H3K4me3 (light green and dark green bars) or H3K27me3 (pink and red bars). Real-time PCR was used to amplify promoter regions of of the indicated genes and measure the concentrations in immunoprecipitated DNA compared to input DNA. The results confirmed the tiling array analysis: In lung, *Tnc* and *Inmt* showed increased H3K4me3 with age, *Peg3* and *Bub1* showed decreased H3K4me3 with age; *E2f7* and *Ptch2* showed increased H3K27me3 with age, *Cdk4* and *Gpc3* showed decreased H3K27me3 with age; in kidney, *Fgf9* and *Fndc4* showed increased H3K4me3 with age, *Mdk* and *Mmp14* showed decreased H3K4me3 with age; *Fat4* and *Lrrc17* showed increased H3K27me3 with age, *Pcdh1* and *Hoxa1* showed decreased H3K27me3 with age. P values (ANOVA) are for change in histone level with age. Number of animals = 5 in each data point.
(TIF)

Figure S6 **Temporal changes in H3K4me3 and H3K27me3 were associated with temporal changes in gene expression in kidney and lung.** Dot plots show that changes in mRNA from 1- to 4-wk was positively correlated (Spearman’s correlation) with changes in H3K4me3 (left panels) and negatively correlated with changes of H3K27me3 (right panels) from 1- to 4-wk in kidney (upper panels) and lung (lower panels). Spearman’s ρ and P-values are indicated at an upper corner of each graph.
(TIF)

Figure S7 **Protein interactions of different sets of genes with concordant changes of histone methylation with age between kidney and lung.** STRING9.0 was used to analyze protein interactions. (**A**) Genes with concordant acquisition of or increase in H3K4me3. (**B**) Genes with concordant loss of or decrease in H3K27me3. (**C**) Genes with concordant acquisition of or increase in H3K27me3. None of these gene sets form an extensive network of protein-protein interactions as compared to genes with concordant loss of or decrease in H3K4me3 with age (Fig. 5).
(TIF)

File S1 **Combined file that contains Table S1–S24.**
(DOCX)
