# Supplementary figures and images for: Broad Shifts in Gene Expression during Early Postnatal Life Are Associated with Shifts in Histone Methylation Patterns
Source: PLoS One. 2014 Jan 28;9(1):e86957. doi: 10.1371/journal.pone.0086957 (PMC3904965; doi:10.1371/journal.pone.0086957)

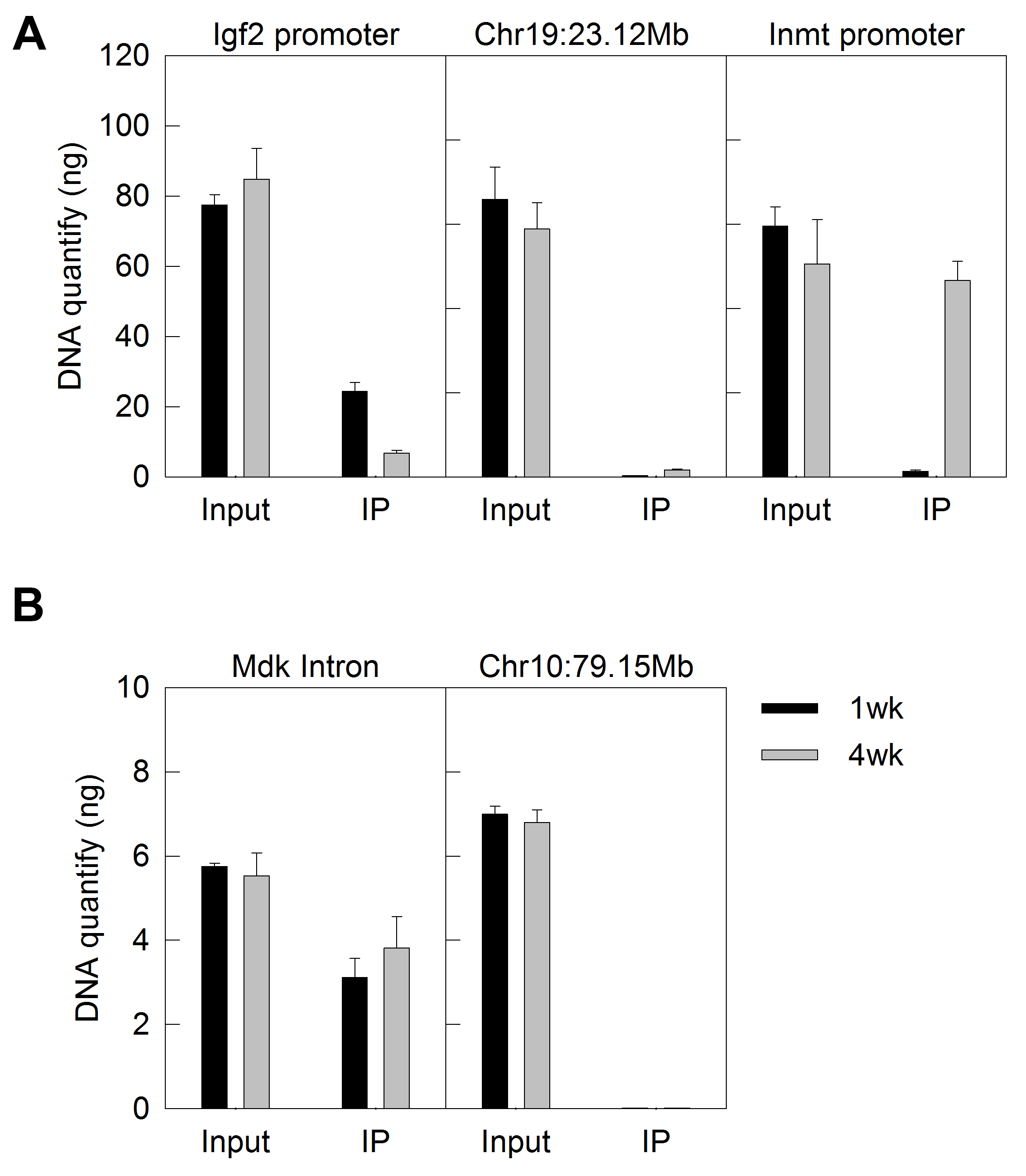

Supplement: Figure S1 — Assessment of DNA quality after chromatin immunoprecipitation. Chromatin from kidney of 1-wk and 4-wk old mice was immunoprecipitated with antibodies to H3K4me3 (A) or H3K27me3 (B). Real-time PCR was used to measure content of indicated genomic regions in the immunoprecipitated DNA compared to input DNA. Igf2 and Inmt served as positive controls for H3K4me3 at 1- and 4-wk, respectively. Chr19∶23.12 Mb was a negative control for H3K4me3. Mdk intronic region was a positive control for H3K27me3, and Chr10∶79.15 Mb was a negative control for H3K27me3. (TIF) [file pone.0086957.s001.tif]

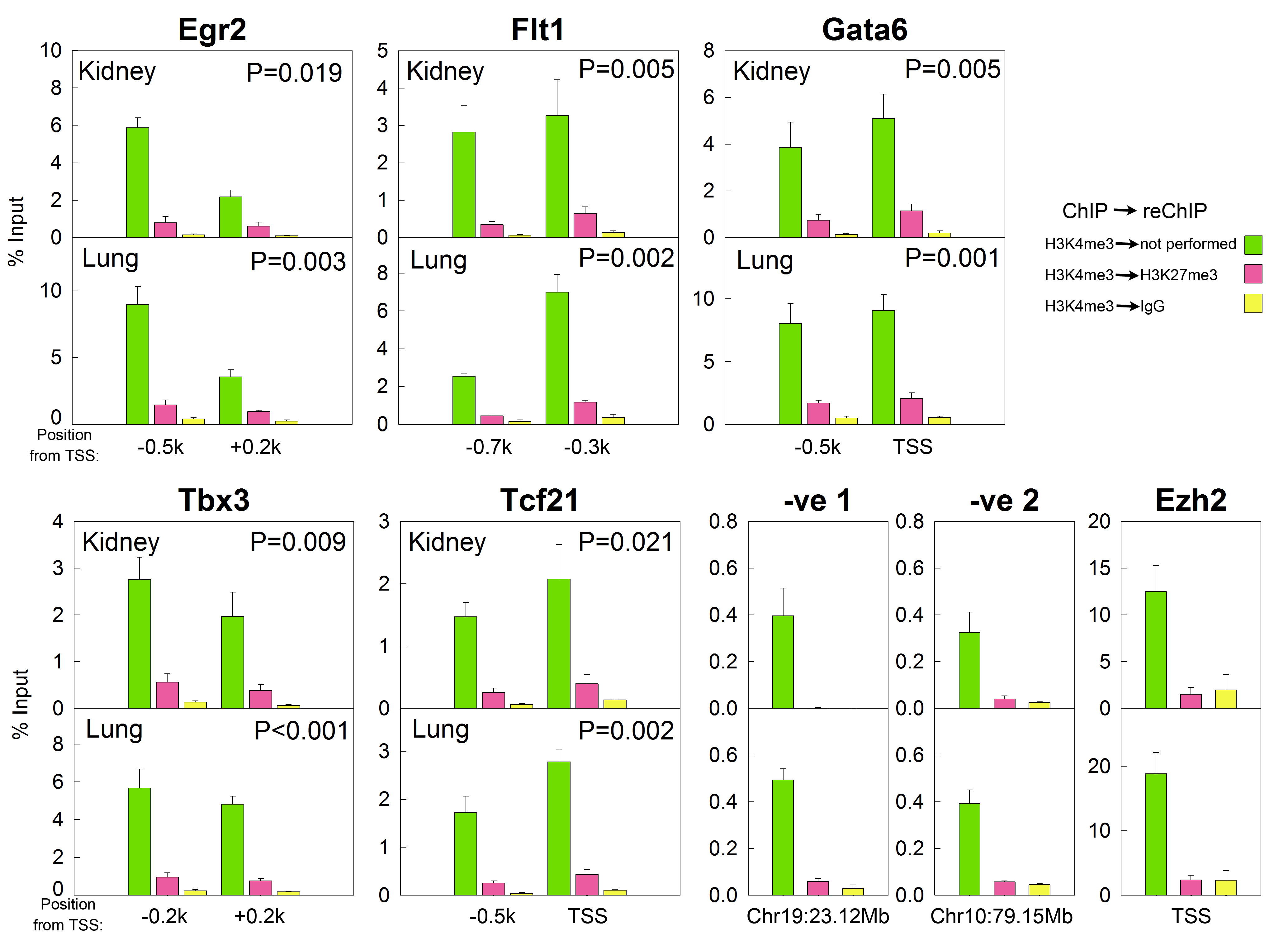

Supplement: Figure S2 — Validation of H3K4me3 and H3K27me3 bivalency using ChIP-reChIP. Chromatin from kidney and lung of 1-wk old mice was immunoprecipitated first with antibodies to H3K4me3, followed by DNA purification (green bar), or a second immunoprecipitation using antibodies to H3K27me3 (red bar) or IgG (yellow bar). Real-time PCR was then used to measure content of indicated genomic regions in the immunoprecipitated DNA compared to input DNA. Chr19∶23.12 Mb and Chr10∶79.15 Mb was used as negative control for both H3K4me3 and H3K27me3. Ezh2 was a positive control for H3K4me3 but negative control for H3K27me3. Two-way-ANOVA was performed using genomic position and effect of antibody in the re-ChIP (H3K27me3 versus IgG) as the two independent variables. P values represent the effect of antibody in the re-ChIP. Number of animals = 5 in each data point. (TIF) [file pone.0086957.s002.tif]

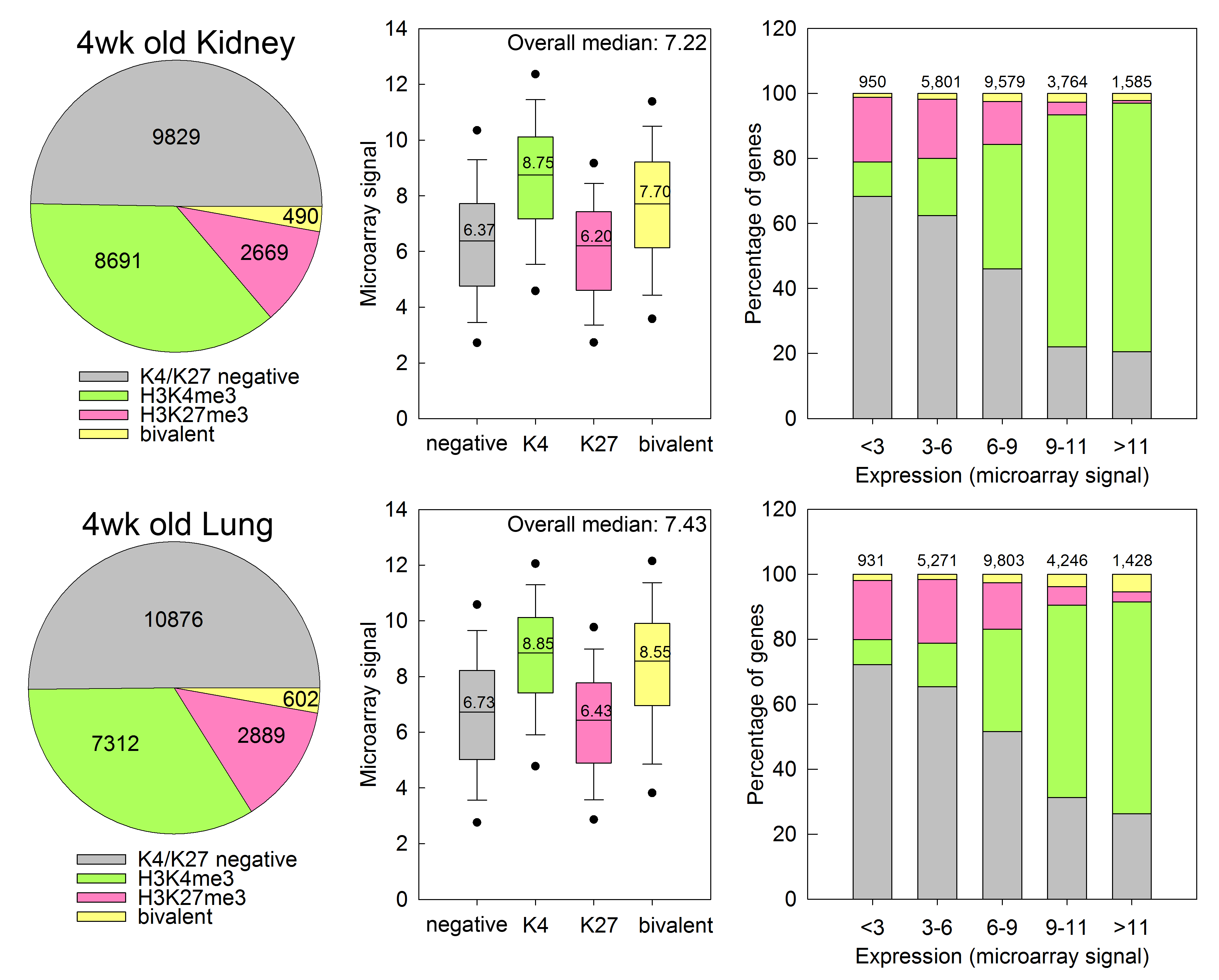

Supplement: Figure S3 — H3K4me3 is associated with high level of gene expression and H3K27me3 is associated with low level of gene expression in kidney and lung of 4-wk-old mice. Pie chart (left) depict the distribution of H3K4me3 and H3K27me3 marks at promoter regions of genes across the genome, in kidney (top) and lung (bottom) of 4-wk old mice. The number of genes within each category are indicated. Box and whisker plots (center) show the expression microarray signals of genes with different types of histone methylation. The line within each box represents the median microarray signal of the genes in the designated gene set, with the median value displayed over the line. The upper and lower boundaries of the box indicate the 75th and 25th percentiles, respectively. Whiskers (error bars) above and below the box indicate the 90th and 10th percentiles and outlying dots indicate the 95th and 5th percentiles. The overall median of the microarray signal from the whole genome is displayed on the upper right corner of each graph. Bar graphs (right) show the distribution of H3K4me3 and H3K27me3 marks in gene sets with different levels of expression. H3K4me3 marks occurred more frequently in genes with higher expression levels, and H3K27me3 marks occurred more frequently in genes with lower expression levels. The values above bars indicate number of genes in each group. The color code for all graphs is shown in below pie charts. (TIF) [file pone.0086957.s003.tif]

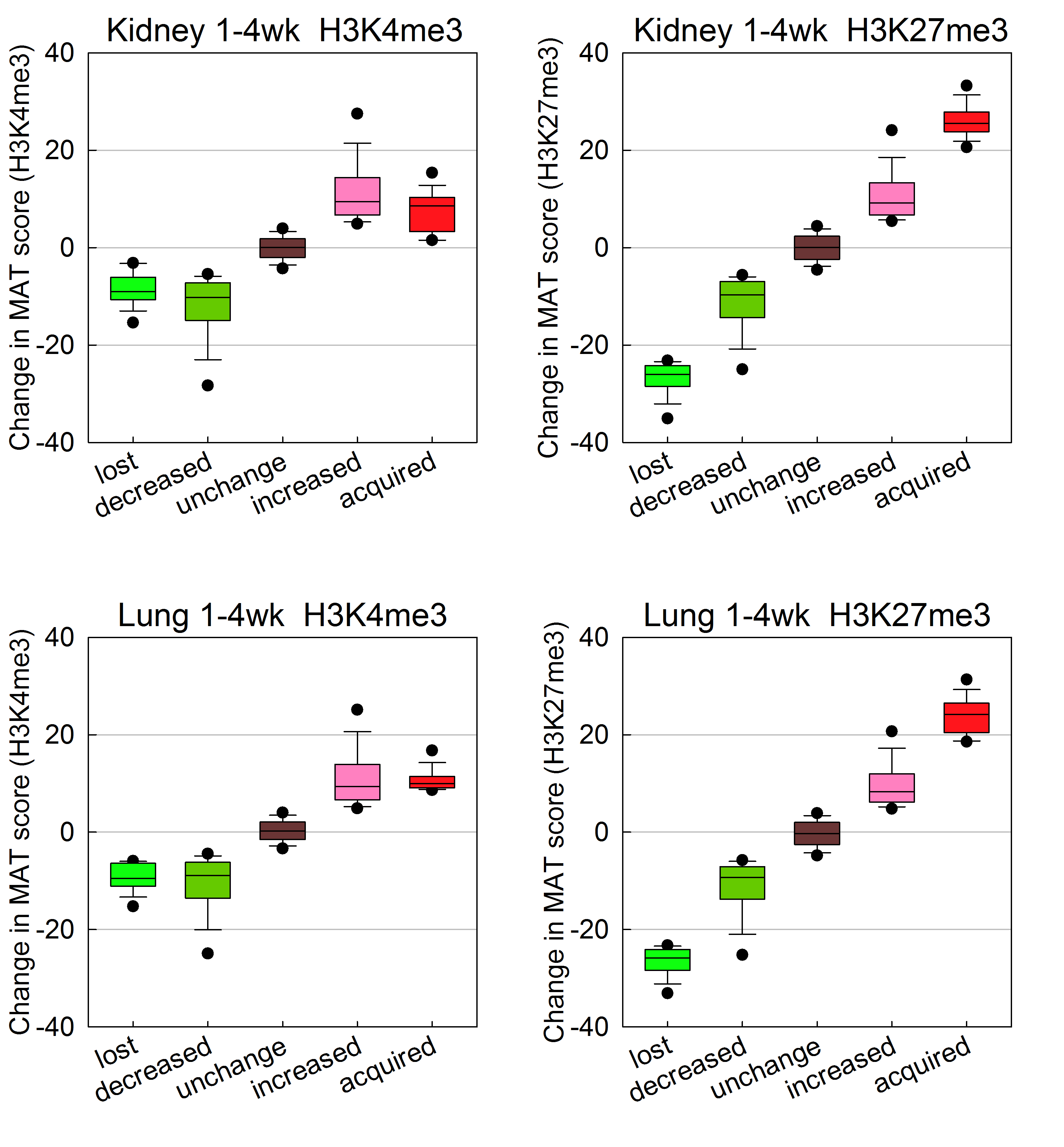

Supplement: Figure S4 — Box and whisker plot showing the change in MAT scores of gene sets as categorized in Fig. 2 . The line within each box represents the median MAT score of the genes in the designated gene set. The upper and lower boundary of the box indicates the 75th and 25th percentiles, respectively. Whiskers (error bars) above and below the box indicate the 90th and 10th percentiles and outlying dots indicate the 95th and 5th percentiles. (TIF) [file pone.0086957.s004.tif]

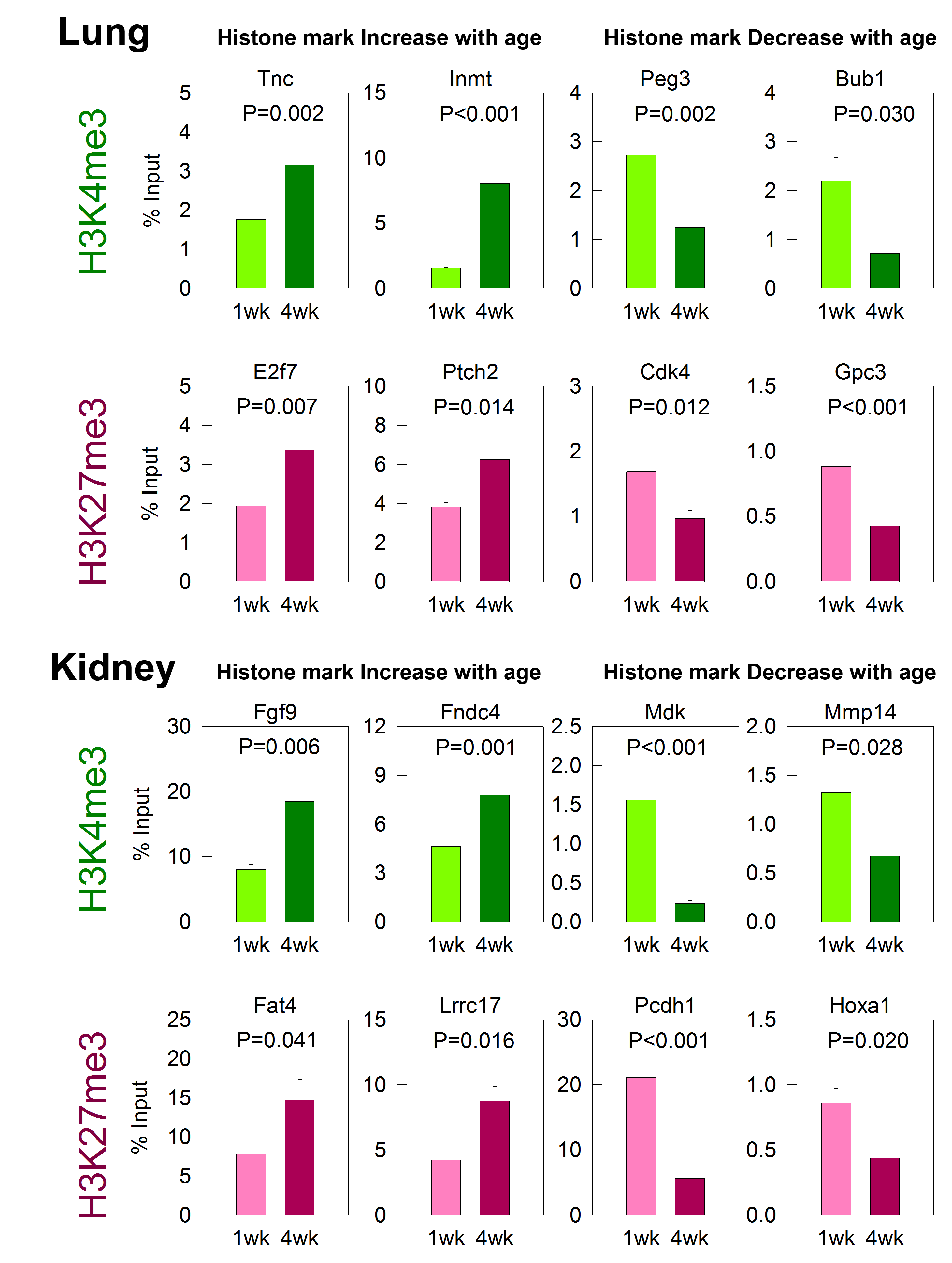

Supplement: Figure S5 — Validation of changes of H3K4me3 and H3K27me3 with age using ChIP-qPCR. Chromatin from lung (top panels) or kidney (bottom panels) of 1-wk and 4-wk old mice was immunoprecipitated with antibodies to H3K4me3 (light green and dark green bars) or H3K27me3 (pink and red bars). Real-time PCR was used to amplify promoter regions of of the indicated genes and measure the concentrations in immunoprecipitated DNA compared to input DNA. The results confirmed the tiling array analysis: In lung, Tnc and Inmt showed increased H3K4me3 with age, Peg3 and Bub1 showed decreased H3K4me3 with age; E2f7 and Ptch2 showed increased H3K27me3 with age, Cdk4 and Gpc3 showed decreased H3K27me3 with age; in kidney, Fgf9 and Fndc4 showed increased H3K4me3 with age, Mdk and Mmp14 showed decreased H3K4me3 with age; Fat4 and Lrrc17 showed increased H3K27me3 with age, Pcdh1 and Hoxa1 showed decreased H3K27me3 with age. P values (ANOVA) are for change in histone level with age. Number of animals = 5 in each data point. (TIF) [file pone.0086957.s005.tif]

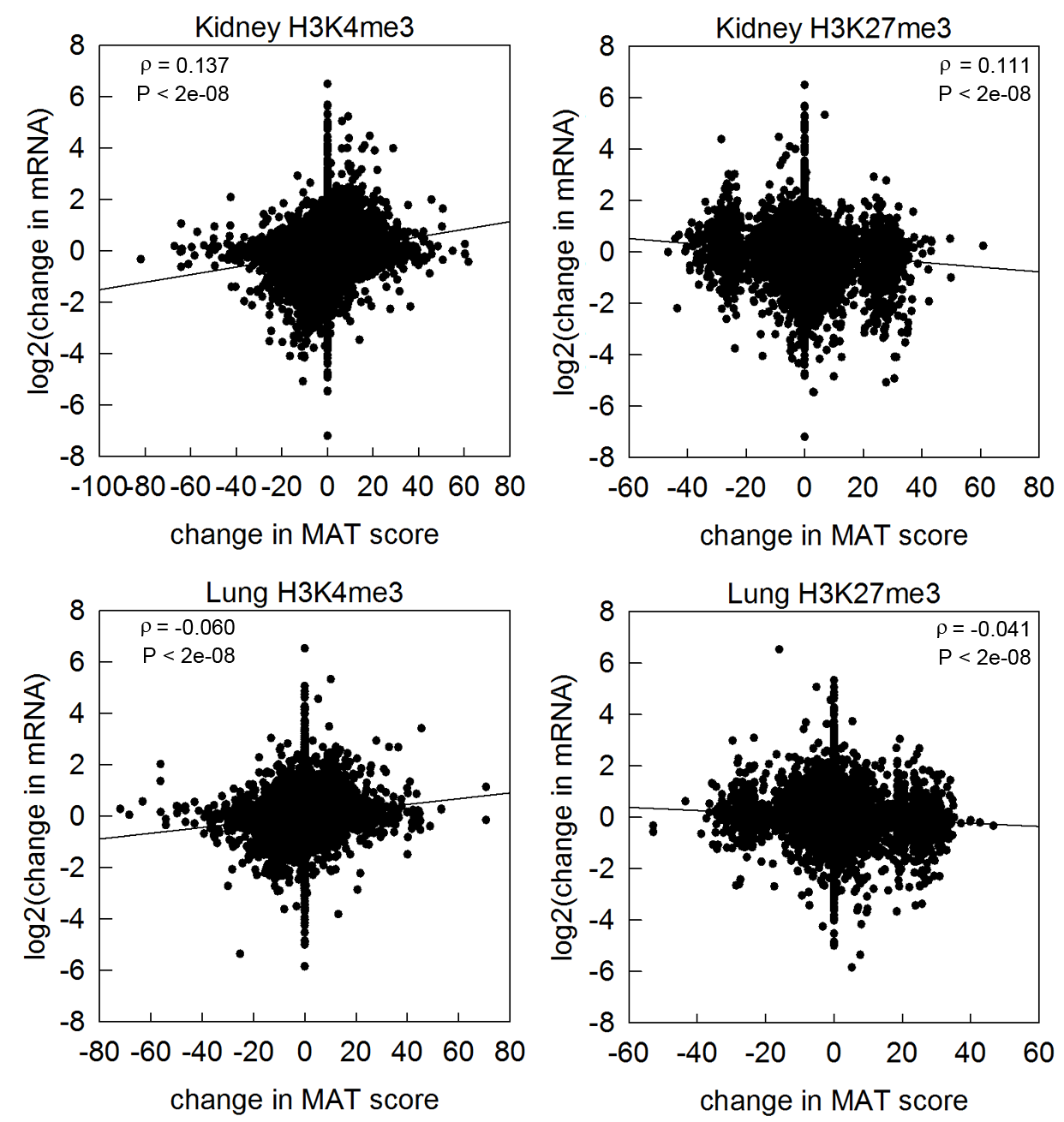

Supplement: Figure S6 — Temporal changes in H3K4me3 and H3K27me3 were associated with temporal changes in gene expression in kidney and lung. Dot plots show that changes in mRNA from 1- to 4-wk was positively correlated (Spearman’s correlation) with changes in H3K4me3 (left panels) and negatively correlated with changes of H3K27me3 (right panels) from 1- to 4-wk in kidney (upper panels) and lung (lower panels). Spearman’s ρ and P-values are indicated at an upper corner of each graph. (TIF) [file pone.0086957.s006.tif]

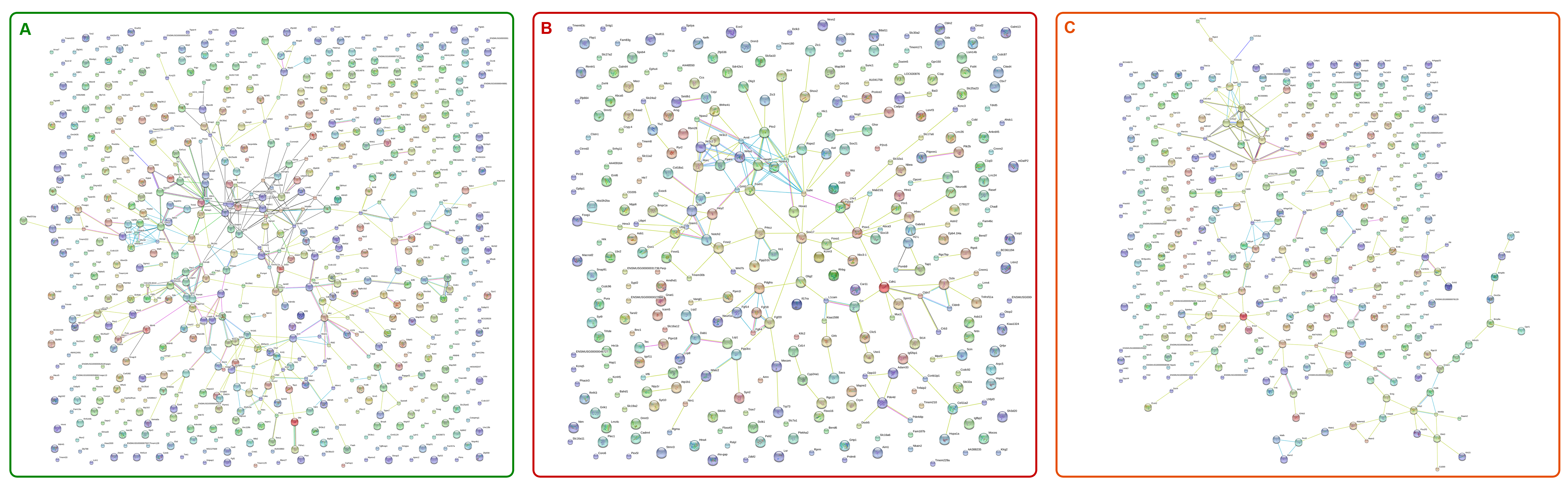

Supplement: Figure S7 — Protein interactions of different sets of genes with concordant changes of histone methylation with age between kidney and lung. STRING9.0 was used to analyze protein interactions. (A) Genes with concordant acquisition of or increase in H3K4me3. (B) Genes with concordant loss of or decrease in H3K27me3. (C) Genes with concordant acquisition of or increase in H3K27me3. None of these gene sets form an extensive network of protein-protein interactions as compared to genes with concordant loss of or decrease in H3K4me3 with age (Fig. 5). (TIF) [file pone.0086957.s007.tif]
