# Supplementary material for: Broad Shifts in Gene Expression during Early Postnatal Life Are Associated with Shifts in Histone Methylation Patterns
Source: PLoS One. 2014 Jan 28;9(1):e86957. doi: 10.1371/journal.pone.0086957 (PMC3904965; doi:10.1371/journal.pone.0086957)
Supplement: File S1 — Combined file that contains Table S1–S24. (DOCX) [file pone.0086957.s008.docx]

**Table S1. Primers for quality control of chromatin immunoprecipitated DNA**

| Genomic regions with positive H3K4me3 signal: | | |
| --- | --- | --- |
| Igf2 promoter | Forward | 5’ CAGCTGACCTCATTTCCCGAT 3’ |
|  | Reverse | 5’ TATGCAAACCGAACAGCGG 3’ |
| Inmt promoter | Forward | 5’ TGGCAATGTGTGCAGATCCAG 3’ |
|  | Reverse | 5’ TTGGTGCCCAGTCCTATGTCA 3’ |
|  |  |  |
| Genomic regions with negative H3K4me3 signal: | | |
| Chr19:23.12Mb | Forward | 5’ TGGACTGCCAGAAAACAGAGC 3’ |
|  | Reverse | 5’ AGCCACGGTGGAAGACATTT 3’ |
|  |  |  |
| Genomic regions with positive H3K27me3 signal: | | |
| Mdk intron 1 | Forward | 5’ CAGCTGACCTCATTTCCCGAT 3’ |
|  | Reverse | 5’ TATGCAAACCGAACAGCGG 3’ |
|  |  |  |
| Genomic regions with negative H3K27me3 signal: | | |
| Chr10:79.15Mb | Forward | 5’ CCCCCAAGCATCATATTCTCA 3’ |
|  | Reverse | 5’ GGCATTGCACTCACCCTTACA 3’ |

**Table S2. Primers for validation of genes that showed bivalency (presence of both H3K4me3 and H3K27me3) in tiling array analysis**

| Genes that showed bivalent histone mark at 1wk of age: | | |  |  |
| --- | --- | --- | --- | --- |
| Egr2 | Forward 1 | 5’ AAAAAGGCAGTCAGCTTCCG 3’ |  |  |
|  | Reverse 1 | 5’ CCCTCCTTCACTTTCTTGGCA 3’ |  |  |
|  | Forward 2 | 5’ TGTGTGGATGTGTTGTGGTGG 3’ |  |  |
|  | Reverse 2 | 5’ CGCCAGAGAAGCCGTTTTTT 3’ |  |  |
| Flt1 | Forward 1 | 5’ ACAAGTTGCAGGAGCGTGTCT 3’ |  |  |
|  | Reverse 1 | 5’ ATTAATGCACCCGCCCAAG 3’ |  |  |
|  | Forward 2 | 5’ TTCCCTAGGATAAGACTTCCCG 3’ |  |  |
|  | Reverse 2 | 5’ AAATCCTTACCCAGCTCCCTC 3’ |  |  |
| Gata6 | Forward 1 | 5’ TTGAATTCCAAAGGACCCCAC 3’ |  |  |
|  | Reverse 1 | 5’ CAGCCCCTCCTTCCAAATTAA 3’ |  |  |
|  | Forward 2 | 5’ CCACCCGCTTTTTTTCTCC 3’ |  |  |
|  | Reverse 2 | 5’ CACCGAGCCCTAAACAAACAG 3’ |  |  |
| Tbx3 | Forward 1 | 5’ GCGAGCCTGGAGCTTTTGTTA 3’ |  |  |
|  | Reverse 1 | 5’ TGCGGATCTGCAGCTTCTT 3’ |  |  |
|  | Forward 2 | 5’ CCTCCCCTCTGATCATGTTGA 3’ |  |  |
|  | Reverse 2 | 5’ TTTTCACAGGTCGAGTGCTGG 3’ |  |  |
| Tcf21 | Forward 1 | 5’ TCCCAGCGATCAGCATTTTC 3’ |  |  |
|  | Reverse 1 | 5’ TACCGTGCACTGGCTAACACA 3’ |  |  |
|  | Forward 2 | 5’ GCGCTCTTTCACTCTCCGTTA 3’ |  |  |
|  | Reverse 2 | 5’ TCCCCCAGCTTTCCCTTTA 3’ |  |  |
|  |  |  |  |  |
|  | | |  |  |
|  |  |  |  |  |
|  |  |  |  |  |
|  |  |  |  |  |
|  |  |  |  |  |
|  |  |  |  |  |
|  |  |  |  |  |
|  |  |  |  |  |
|  |  |  |  |  |
|  |  |  |  |  |
|  |  |  |  |  |
|  |  |  |  |  |
|  |  |  |  |  |
|  |  |  |  |  |

**Table S3. Primers for validation of genes that showed changes in histone modification with age in tiling array analysis**

| *Genes that showed increased H3K4me3 with age in Lung:* | | |
| --- | --- | --- |
| Tnc | Forward | 5’ CCCAAGGCTTCTGTTTCACAA 3’ |
|  | Reverse | 5’ TAGGAATTTCAGCTCCCTCCG 3’ |
| Inmt | Forward | 5’ TGGCAATGTGTGCAGATCCAG 3’ |
|  | Reverse | 5’ TTGGTGCCCAGTCCTATGTCA 3’ |
| *Genes that showed decreased H3K4me3 with age in Lung:* | | |
| Peg3 | Forward | 5’ GATTTTGTACGCCTGCCCGTT 3’ |
|  | Reverse | 5’ CAGTGGATAATGCCTTCCCCA 3’ |
| Bub1 | Forward | 5’ CACGTTCGACATCACAAGTGC 3’ |
|  | Reverse | 5’ CCTGACCGACACTTACCGAAA 3’ |
| *Genes that showed increased H3K27me3 with age in Lung:* | | |
| E2f7 | Forward | 5’ ACTACCACCTACCCCCACGATT 3’ |
|  | Reverse | 5’ ATCTAGAGACCAACCCATGCGG 3’ |
| Ptch2 | Forward | 5’ TGCTCCTGTGCATCTTTACGC 3’ |
|  | Reverse | 5’ CGTTATCTGAGCGCTGCCATA 3’ |
| *Genes that showed decreased H3K27me3 with age in Lung:* | | |
| Cdk4 | Forward | 5’ ATCGCATACCAACTGGAAGGA 3’ |
|  | Reverse | 5’ TGGTGCCAGAAATGGTGCT 3’ |
| Gpc3 | Forward | 5’ CTGCGAGGAAACTTTTGCG 3’ |
|  | Reverse | 5’ AGAGTGGCAATAAGAGCCGGA 3’ |
| *Genes that showed increased H3K4me3 with age in Kidney:* | | |
| Fgf9 | Forward | 5’ GCCCGAGCATCTTAAAAATCC 3’ |
|  | Reverse | 5’ CATCCCATCCGACCGTAATAA 3’ |
| Fndc4 | Forward | 5’ CGCAGTCTCCAAGAGGTTGTT 3’ |
|  | Reverse | 5’ TCCCCGCATGAAATTAGCC 3’ |
| *Genes that showed decreased H3K4me3 with age in Kidney:* | | |
| Mdk | Forward | 5’ GCTGAGACATCGGTTCCAAGT 3’ |
|  | Reverse | 5’ TCGCCTAGTCTCTTCTCCGCTA 3’ |
| Mmp14 | Forward | 5’ GCCTGCACCACAGAAAAGACA 3’ |
|  | Reverse | 5’ TCTGCTTAGTCGGCGAACTGA 3’ |
| *Genes that showed increased H3K27me3 with age in Kidney:* | | |
| Fat4 | Forward | 5’ AAACTGGCTTTTTACTGGCGC 3’ |
|  | Reverse | 5’ GTTTGCGGCCAAGTTCATG 3’ |
| Lrrc17 | Forward | 5’ TGGTCCGTTTCATCATCCAA 3’ |
|  | Reverse | 5’ TTACATGGCCACAGCTGCTTC 3’ |
| *Genes that showed decreased H3K27me3 with age in Kidney:* | | |
| Pcdh1 | Forward | 5’ AGCGACCAAAACCTTAGGAGC 3’ |
|  | Reverse | 5’ TTAACCAGCAAGAGCAGCCCT 3’ |
| Hoxa1 | Forward | 5’ AGCCATTGGCTGGTAGAGTCA 3’ |
|  | Reverse | 5’ CCGTGCCAACTTTCTCACTTC 3’ |
|  |  |  |
|  |  |  |
|  |  |  |

**Table S4. Primers for validation of genes that showed concordant decreased H3K4me3 with age in tiling array analysis and decreased gene expression with age in expression microarray analysis**

| Genes that showed concordant decline in H3K4me3 from 1- to 4-wk: | | |
| --- | --- | --- |
| Aurkb | Forward | 5’ AGGCCACACCTTCGTTCTGTT 3’ |
|  | Reverse | 5’ GCTTTCGAATCTCCCGCTCTA 3’ |
| Ccnd2 | Forward | 5’ TATGCCCCCATGGTATGCCTA 3’ |
|  | Reverse | 5’ TTCAAACACGATCCCCGCT 3’ |
| Sox11 | Forward | 5’ ATTTCTAGACAACTCCCCCCG 3’ |
|  | Reverse | 5’ TCACTACTCCCACCAGCCAAT 3’ |
| Rrm2 | Forward | 5’ TTAATCCCACAAGGCCGGT 3’ |
|  | Reverse | 5’ TTAGGATTGCGAGAGCCTTGG 3’ |
| Zwilch | Forward | 5’ CGGAGAAAACACCCGAGCTTA 3’ |
|  | Reverse | 5’ TGTCCGTTTCTGAGCTTGAGG 3’ |
|  |  |  |
|  |  |  |
|  |  |  |

**Table S5. 50 most significant biological functions implicated by DAVID gene ontology analysis in genes showing bivalent methylation (H3K4me3 and H3K27me3) in** **kidney of 1-wk old mice**

| **GO number** | **GO Term** | **Count** | **P-Value** | **Fold Enrichment** | **FDR** |
| --- | --- | --- | --- | --- | --- |
| GO:0035295 | tube development | 42 | 1.40E-16 | 4.71 | 1.89E-13 |
| GO:0001655 | urogenital system development | 29 | 5.50E-14 | 5.88 | 9.53E-11 |
| GO:0001822 | kidney development | 23 | 6.76E-12 | 6.36 | 1.17E-08 |
| GO:0001501 | skeletal system development | 37 | 7.24E-12 | 3.84 | 1.25E-08 |
| GO:0060429 | epithelium development | 36 | 7.55E-12 | 3.93 | 1.31E-08 |
| GO:0001944 | vasculature development | 34 | 1.69E-11 | 4.03 | 2.92E-08 |
| GO:0035239 | tube morphogenesis | 28 | 1.91E-11 | 4.85 | 3.31E-08 |
| GO:0002009 | morphogenesis of an epithelium | 28 | 2.53E-11 | 4.79 | 4.38E-08 |
| GO:0007389 | pattern specification process | 36 | 2.88E-11 | 3.75 | 4.98E-08 |
| GO:0001568 | blood vessel development | 33 | 4.10E-11 | 4.00 | 7.10E-08 |
| GO:0001656 | metanephros development | 17 | 4.65E-11 | 8.68 | 8.05E-08 |
| GO:0048729 | tissue morphogenesis | 32 | 9.97E-11 | 3.98 | 1.73E-07 |
| GO:0048568 | embryonic organ development | 32 | 1.37E-10 | 3.93 | 2.38E-07 |
| GO:0003002 | regionalization | 30 | 1.56E-10 | 4.15 | 2.70E-07 |
| GO:0001763 | morphogenesis of a branching structure | 23 | 1.68E-10 | 5.45 | 2.91E-07 |
| GO:0009952 | anterior/posterior pattern formation | 25 | 2.96E-10 | 4.84 | 5.12E-07 |
| GO:0048598 | embryonic morphogenesis | 39 | 3.76E-10 | 3.22 | 6.52E-07 |
| GO:0048754 | branching morphogenesis of a tube | 19 | 1.60E-09 | 6.05 | 2.77E-06 |
| GO:0007507 | heart development | 29 | 1.91E-09 | 3.85 | 3.30E-06 |
| GO:0010628 | positive regulation of gene expression | 45 | 2.51E-09 | 2.73 | 4.34E-06 |
| GO:0048732 | gland development | 27 | 2.51E-09 | 4.06 | 4.35E-06 |
| GO:0048736 | appendage development | 21 | 2.66E-09 | 5.22 | 4.61E-06 |
| GO:0060173 | limb development | 21 | 2.66E-09 | 5.22 | 4.61E-06 |
| GO:0048514 | blood vessel morphogenesis | 27 | 2.80E-09 | 4.04 | 4.85E-06 |
| GO:0043009 | chordate embryonic development | 41 | 3.13E-09 | 2.88 | 5.42E-06 |
| GO:0009792 | embryonic development ending in birth or egg hatching | 41 | 4.09E-09 | 2.86 | 7.07E-06 |
| GO:0051216 | cartilage development | 17 | 5.38E-09 | 6.45 | 9.31E-06 |
| GO:0045893 | positive regulation of transcription, DNA-dependent | 40 | 7.21E-09 | 2.85 | 1.25E-05 |
| GO:0051254 | positive regulation of RNA metabolic process | 40 | 8.80E-09 | 2.83 | 1.52E-05 |
| GO:0035108 | limb morphogenesis | 20 | 8.95E-09 | 5.15 | 1.55E-05 |
| GO:0035107 | appendage morphogenesis | 20 | 8.95E-09 | 5.15 | 1.55E-05 |
| GO:0045941 | positive regulation of transcription | 43 | 1.05E-08 | 2.68 | 1.82E-05 |
| GO:0048705 | skeletal system morphogenesis | 21 | 1.29E-08 | 4.78 | 2.23E-05 |
| GO:0045944 | positive regulation of transcription from RNA polymerase II promoter | 36 | 1.53E-08 | 2.98 | 2.64E-05 |
| GO:0045165 | cell fate commitment | 22 | 2.15E-08 | 4.43 | 3.72E-05 |
| GO:0030326 | embryonic limb morphogenesis | 18 | 2.19E-08 | 5.49 | 3.79E-05 |
| GO:0035113 | embryonic appendage morphogenesis | 18 | 2.19E-08 | 5.49 | 3.79E-05 |
| GO:0048562 | embryonic organ morphogenesis | 23 | 2.27E-08 | 4.23 | 3.93E-05 |
| GO:0009891 | positive regulation of biosynthetic process | 46 | 4.68E-08 | 2.44 | 8.09E-05 |
| GO:0051173 | positive regulation of nitrogen compound metabolic process | 44 | 6.84E-08 | 2.48 | 1.18E-04 |
| GO:0045935 | positive regulation of nucleobase, nucleoside, nucleotide and nucleic acid metabolic process | 43 | 7.99E-08 | 2.50 | 1.38E-04 |
| GO:0010557 | positive regulation of macromolecule biosynthetic process | 44 | 8.45E-08 | 2.46 | 1.46E-04 |
| GO:0001657 | ureteric bud development | 12 | 9.42E-08 | 8.46 | 1.63E-04 |
| GO:0031328 | positive regulation of cellular biosynthetic process | 45 | 9.91E-08 | 2.41 | 1.71E-04 |
| GO:0008284 | positive regulation of cell proliferation | 30 | 1.06E-07 | 3.13 | 1.83E-04 |
| GO:0042127 | regulation of cell proliferation | 44 | 1.29E-07 | 2.42 | 2.23E-04 |
| GO:0006350 | transcription | 100 | 1.64E-07 | 1.67 | 2.84E-04 |
| GO:0048704 | embryonic skeletal system morphogenesis | 14 | 1.67E-07 | 6.48 | 2.90E-04 |
| GO:0060562 | epithelial tube morphogenesis | 18 | 1.71E-07 | 4.80 | 2.96E-04 |
| GO:0003006 | reproductive developmental process | 28 | 2.86E-07 | 3.14 | 4.95E-04 |

**Table S6. 50 most significant biological functions implicated by DAVID gene ontology analysis genes showing bivalent methylation (H3K4me3 and H3K27me3) in lung of 1-wk old mice**

| **GO number** | **GO Term** | **Count** | **P-Value** | **Fold Enrichment** | **FDR** |
| --- | --- | --- | --- | --- | --- |
| GO:0035295 | tube development | 42 | 1.38E-17 | 5.03 | 2.40E-14 |
| GO:0048568 | embryonic organ development | 39 | 1.32E-16 | 5.11 | 1.89E-13 |
| GO:0002009 | morphogenesis of an epithelium | 32 | 2.76E-15 | 5.85 | 4.83E-12 |
| GO:0048729 | tissue morphogenesis | 36 | 2.29E-14 | 4.78 | 3.98E-11 |
| GO:0060429 | epithelium development | 38 | 4.40E-14 | 4.43 | 7.67E-11 |
| GO:0001501 | skeletal system development | 38 | 2.16E-13 | 4.21 | 3.76E-10 |
| GO:0030324 | lung development | 24 | 4.83E-13 | 6.83 | 8.40E-10 |
| GO:0030323 | respiratory tube development | 24 | 7.20E-13 | 6.71 | 1.25E-09 |
| GO:0048598 | embryonic morphogenesis | 41 | 3.35E-12 | 3.61 | 5.82E-09 |
| GO:0060541 | respiratory system development | 24 | 5.56E-12 | 6.12 | 9.67E-09 |
| GO:0048562 | embryonic organ morphogenesis | 27 | 6.25E-12 | 5.30 | 1.09E-08 |
| GO:0043009 | chordate embryonic development | 44 | 9.24E-12 | 3.30 | 1.61E-08 |
| GO:0009792 | embryonic development ending in birth or egg hatching | 44 | 1.26E-11 | 3.27 | 2.20E-08 |
| GO:0001763 | morphogenesis of a branching structure | 23 | 4.78E-11 | 5.81 | 8.31E-08 |
| GO:0001944 | vasculature development | 32 | 6.81E-11 | 4.04 | 1.19E-07 |
| GO:0048754 | branching morphogenesis of a tube | 20 | 6.91E-11 | 6.80 | 1.20E-07 |
| GO:0048704 | embryonic skeletal system morphogenesis | 17 | 8.98E-11 | 8.39 | 1.56E-07 |
| GO:0048705 | skeletal system morphogenesis | 23 | 1.06E-10 | 5.59 | 1.85E-07 |
| GO:0001568 | blood vessel development | 31 | 1.73E-10 | 4.01 | 3.00E-07 |
| GO:0048706 | embryonic skeletal system development | 18 | 6.75E-10 | 6.85 | 1.17E-06 |
| GO:0006357 | regulation of transcription from RNA polymerase II promoter | 51 | 7.32E-10 | 2.62 | 1.27E-06 |
| GO:0035239 | tube morphogenesis | 25 | 8.17E-10 | 4.62 | 1.42E-06 |
| GO:0060562 | epithelial tube morphogenesis | 20 | 1.66E-09 | 5.69 | 2.89E-06 |
| GO:0007389 | pattern specification process | 32 | 1.68E-09 | 3.56 | 2.93E-06 |
| GO:0042127 | regulation of cell proliferation | 46 | 2.19E-09 | 2.70 | 3.82E-06 |
| GO:0051252 | regulation of RNA metabolic process | 89 | 3.15E-09 | 1.89 | 5.48E-06 |
| GO:0048514 | blood vessel morphogenesis | 26 | 3.42E-09 | 4.15 | 5.95E-06 |
| GO:0006355 | regulation of transcription, DNA-dependent | 87 | 7.20E-09 | 1.88 | 1.25E-05 |
| GO:0051216 | cartilage development | 16 | 1.71E-08 | 6.48 | 2.98E-05 |
| GO:0045165 | cell fate commitment | 21 | 3.66E-08 | 4.51 | 6.36E-05 |
| GO:0045449 | regulation of transcription | 115 | 3.72E-08 | 1.63 | 6.48E-05 |
| GO:0045892 | negative regulation of transcription, DNA-dependent | 31 | 4.32E-08 | 3.18 | 7.52E-05 |
| GO:0051253 | negative regulation of RNA metabolic process | 31 | 5.00E-08 | 3.16 | 8.70E-05 |
| GO:0003002 | regionalization | 25 | 7.24E-08 | 3.69 | 1.26E-04 |
| GO:0008284 | positive regulation of cell proliferation | 29 | 9.56E-08 | 3.23 | 1.66E-04 |
| GO:0007507 | heart development | 25 | 1.58E-07 | 3.54 | 2.75E-04 |
| GO:0001655 | urogenital system development | 20 | 1.65E-07 | 4.33 | 2.87E-04 |
| GO:0048736 | appendage development | 18 | 1.90E-07 | 4.78 | 3.30E-04 |
| GO:0060173 | limb development | 18 | 1.90E-07 | 4.78 | 3.30E-04 |
| GO:0016481 | negative regulation of transcription | 33 | 2.74E-07 | 2.80 | 4.76E-04 |
| GO:0048732 | gland development | 23 | 2.75E-07 | 3.69 | 4.78E-04 |
| GO:0000122 | negative regulation of transcription from RNA polymerase II promoter | 25 | 3.06E-07 | 3.42 | 5.32E-04 |
| GO:0045934 | negative regulation of nucleobase, nucleoside, nucleotide and nucleic acid metabolic process | 34 | 3.91E-07 | 2.71 | 6.79E-04 |
| GO:0051172 | negative regulation of nitrogen compound metabolic process | 34 | 4.91E-07 | 2.68 | 8.53E-04 |
| GO:0006350 | transcription | 93 | 6.51E-07 | 1.66 | 0.0011331 |
| GO:0010629 | negative regulation of gene expression | 34 | 8.04E-07 | 2.62 | 0.0013977 |
| GO:0031327 | negative regulation of cellular biosynthetic process | 35 | 8.22E-07 | 2.57 | 0.0014292 |
| GO:0009890 | negative regulation of biosynthetic process | 35 | 1.03E-06 | 2.55 | 0.0017925 |
| GO:0010558 | negative regulation of macromolecule biosynthetic process | 34 | 1.24E-06 | 2.57 | 0.0021625 |
| GO:0009952 | anterior/posterior pattern formation | 19 | 1.58E-06 | 3.92 | 0.0027408 |

**Table S7. 50 most significant biological functions implicated in 1wk kidney H3K4me3 marked genes by DAVID gene ontology analysis**

| **GO number** | **GO Term** | **Count** | **P-Value** | **Fold Enrichment** | **FDR** |
| --- | --- | --- | --- | --- | --- |
| GO:0006396 | RNA processing | 343 | 2.65E-65 | 2.02 | 5.04E-62 |
| GO:0045184 | establishment of protein localization | 466 | 5.46E-65 | 1.83 | 1.04E-61 |
| GO:0008104 | protein localization | 517 | 2.23E-64 | 1.76 | 4.23E-61 |
| GO:0015031 | protein transport | 462 | 3.32E-64 | 1.82 | 6.30E-61 |
| GO:0046907 | intracellular transport | 323 | 3.11E-53 | 1.93 | 5.91E-50 |
| GO:0009057 | macromolecule catabolic process | 424 | 1.24E-42 | 1.67 | 2.35E-39 |
| GO:0044265 | cellular macromolecule catabolic process | 399 | 1.06E-41 | 1.68 | 2.01E-38 |
| GO:0030163 | protein catabolic process | 368 | 6.65E-40 | 1.70 | 1.26E-36 |
| GO:0007049 | cell cycle | 395 | 2.91E-39 | 1.66 | 5.53E-36 |
| GO:0006974 | response to DNA damage stimulus | 219 | 3.68E-38 | 1.96 | 6.99E-35 |
| GO:0006259 | DNA metabolic process | 293 | 6.66E-38 | 1.79 | 1.26E-34 |
| GO:0016071 | mRNA metabolic process | 225 | 2.14E-36 | 1.91 | 4.07E-33 |
| GO:0044257 | cellular protein catabolic process | 351 | 2.43E-36 | 1.68 | 4.61E-33 |
| GO:0033554 | cellular response to stress | 281 | 2.77E-36 | 1.79 | 5.26E-33 |
| GO:0051603 | proteolysis involved in cellular protein catabolic process | 349 | 4.05E-36 | 1.68 | 7.69E-33 |
| GO:0006412 | translation | 233 | 2.18E-35 | 1.88 | 4.14E-32 |
| GO:0019941 | modification-dependent protein catabolic process | 334 | 2.43E-35 | 1.69 | 4.61E-32 |
| GO:0043632 | modification-dependent macromolecule catabolic process | 334 | 2.43E-35 | 1.69 | 4.61E-32 |
| GO:0034660 | ncRNA metabolic process | 165 | 2.51E-35 | 2.10 | 4.77E-32 |
| GO:0006281 | DNA repair | 175 | 1.03E-33 | 2.03 | 1.95E-30 |
| GO:0006886 | intracellular protein transport | 206 | 1.53E-33 | 1.92 | 2.90E-30 |
| GO:0070727 | cellular macromolecule localization | 219 | 7.10E-33 | 1.87 | 1.35E-29 |
| GO:0034613 | cellular protein localization | 217 | 2.56E-32 | 1.87 | 4.85E-29 |
| GO:0006397 | mRNA processing | 196 | 3.64E-32 | 1.92 | 6.92E-29 |
| GO:0034470 | ncRNA processing | 133 | 3.90E-31 | 2.16 | 7.41E-28 |
| GO:0006350 | transcription | 911 | 1.25E-30 | 1.32 | 2.37E-27 |
| GO:0008380 | RNA splicing | 158 | 3.14E-30 | 2.02 | 5.96E-27 |
| GO:0000278 | mitotic cell cycle | 181 | 5.64E-29 | 1.91 | 1.07E-25 |
| GO:0051301 | cell division | 201 | 1.31E-28 | 1.84 | 2.48E-25 |
| GO:0007067 | mitosis | 148 | 1.35E-27 | 2.00 | 2.57E-24 |
| GO:0000280 | nuclear division | 148 | 1.35E-27 | 2.00 | 2.57E-24 |
| GO:0048285 | organelle fission | 152 | 1.56E-27 | 1.98 | 2.96E-24 |
| GO:0000087 | M phase of mitotic cell cycle | 149 | 1.24E-26 | 1.97 | 2.35E-23 |
| GO:0022613 | ribonucleoprotein complex biogenesis | 113 | 5.13E-25 | 2.12 | 9.75E-22 |
| GO:0022402 | cell cycle process | 253 | 7.16E-25 | 1.65 | 1.36E-21 |
| GO:0022403 | cell cycle phase | 218 | 3.62E-24 | 1.71 | 6.89E-21 |
| GO:0000279 | M phase | 190 | 6.32E-22 | 1.73 | 1.20E-18 |
| GO:0006399 | tRNA metabolic process | 94 | 2.12E-21 | 2.14 | 4.03E-18 |
| GO:0042254 | ribosome biogenesis | 92 | 3.16E-20 | 2.11 | 6.01E-17 |
| GO:0051276 | chromosome organization | 247 | 6.52E-20 | 1.57 | 1.24E-16 |
| GO:0016568 | chromatin modification | 161 | 9.34E-20 | 1.75 | 1.77E-16 |
| GO:0045449 | regulation of transcription | 1056 | 1.25E-19 | 1.22 | 2.37E-16 |
| GO:0048193 | Golgi vesicle transport | 50 | 3.60E-17 | 2.47 | 6.84E-14 |
| GO:0006364 | rRNA processing | 64 | 2.28E-16 | 2.22 | 4.22E-13 |
| GO:0006091 | generation of precursor metabolites and energy | 167 | 3.40E-16 | 1.64 | 6.33E-13 |
| GO:0016072 | rRNA metabolic process | 64 | 9.86E-16 | 2.19 | 1.90E-12 |
| GO:0016192 | vesicle-mediated transport | 266 | 1.17E-15 | 1.47 | 2.32E-12 |
| GO:0006605 | protein targeting | 98 | 1.35E-15 | 1.89 | 2.53E-12 |
| GO:0006325 | chromatin organization | 191 | 6.58E-15 | 1.56 | 1.24E-11 |
| GO:0006457 | protein folding | 93 | 1.42E-14 | 1.88 | 2.70E-11 |

**Table S8. 50 most significant biological functions implicated in 1wk lung H3K4me3 marked genes by DAVID gene ontology analysis**

| **GO number** | **GO Term** | **Count** | **P-Value** | **Fold Enrichment** | **FDR** |
| --- | --- | --- | --- | --- | --- |
| GO:0006396 | RNA processing | 336 | 1.77E-62 | 2.02 | 3.35E-59 |
| GO:0045184 | establishment of protein localization | 450 | 1.43E-58 | 1.80 | 2.72E-55 |
| GO:0015031 | protein transport | 446 | 8.73E-58 | 1.80 | 1.66E-54 |
| GO:0008104 | protein localization | 498 | 2.88E-57 | 1.73 | 5.46E-54 |
| GO:0046907 | intracellular transport | 310 | 7.35E-47 | 1.89 | 1.39E-43 |
| GO:0006974 | response to DNA damage stimulus | 226 | 5.43E-45 | 2.06 | 1.03E-41 |
| GO:0009057 | macromolecule catabolic process | 423 | 1.06E-44 | 1.70 | 2.01E-41 |
| GO:0044265 | cellular macromolecule catabolic process | 399 | 3.84E-44 | 1.72 | 7.29E-41 |
| GO:0007049 | cell cycle | 399 | 1.27E-43 | 1.71 | 2.40E-40 |
| GO:0006259 | DNA metabolic process | 299 | 2.14E-43 | 1.86 | 4.05E-40 |
| GO:0030163 | protein catabolic process | 368 | 3.56E-42 | 1.74 | 6.75E-39 |
| GO:0033554 | cellular response to stress | 286 | 4.45E-41 | 1.86 | 8.45E-38 |
| GO:0006281 | DNA repair | 181 | 7.00E-40 | 2.14 | 1.33E-36 |
| GO:0016071 | mRNA metabolic process | 227 | 2.43E-39 | 1.97 | 4.60E-36 |
| GO:0044257 | cellular protein catabolic process | 352 | 5.72E-39 | 1.72 | 1.08E-35 |
| GO:0051603 | proteolysis involved in cellular protein catabolic process | 350 | 9.83E-39 | 1.72 | 1.87E-35 |
| GO:0019941 | modification-dependent protein catabolic process | 333 | 7.24E-37 | 1.72 | 1.37E-33 |
| GO:0043632 | modification-dependent macromolecule catabolic process | 333 | 7.24E-37 | 1.72 | 1.37E-33 |
| GO:0006397 | mRNA processing | 199 | 1.26E-35 | 1.99 | 2.40E-32 |
| GO:0006412 | translation | 228 | 8.17E-34 | 1.87 | 1.55E-30 |
| GO:0008380 | RNA splicing | 159 | 3.73E-32 | 2.07 | 7.09E-29 |
| GO:0034660 | ncRNA metabolic process | 159 | 1.07E-31 | 2.06 | 2.03E-28 |
| GO:0070727 | cellular macromolecule localization | 213 | 1.23E-30 | 1.86 | 2.34E-27 |
| GO:0006350 | transcription | 896 | 1.92E-30 | 1.33 | 3.65E-27 |
| GO:0000278 | mitotic cell cycle | 181 | 3.21E-30 | 1.95 | 6.08E-27 |
| GO:0034613 | cellular protein localization | 211 | 4.36E-30 | 1.85 | 8.27E-27 |
| GO:0006886 | intracellular protein transport | 198 | 8.93E-30 | 1.88 | 1.69E-26 |
| GO:0000087 | M phase of mitotic cell cycle | 149 | 1.09E-27 | 2.01 | 2.07E-24 |
| GO:0022402 | cell cycle process | 255 | 2.62E-27 | 1.70 | 4.98E-24 |
| GO:0000280 | nuclear division | 146 | 3.50E-27 | 2.01 | 6.63E-24 |
| GO:0007067 | mitosis | 146 | 3.50E-27 | 2.01 | 6.63E-24 |
| GO:0048285 | organelle fission | 150 | 3.57E-27 | 2.00 | 6.78E-24 |
| GO:0051301 | cell division | 196 | 5.75E-27 | 1.83 | 1.09E-23 |
| GO:0034470 | ncRNA processing | 126 | 3.52E-26 | 2.09 | 6.68E-23 |
| GO:0022613 | ribonucleoprotein complex biogenesis | 113 | 7.25E-26 | 2.16 | 1.38E-22 |
| GO:0022403 | cell cycle phase | 218 | 1.73E-25 | 1.74 | 3.28E-22 |
| GO:0000279 | M phase | 190 | 4.36E-23 | 1.76 | 8.27E-20 |
| GO:0045449 | regulation of transcription | 1039 | 9.04E-20 | 1.22 | 1.71E-16 |
| GO:0042254 | ribosome biogenesis | 90 | 2.87E-19 | 2.11 | 5.44E-16 |
| GO:0006399 | tRNA metabolic process | 90 | 8.40E-19 | 2.09 | 1.59E-15 |
| GO:0051276 | chromosome organization | 239 | 4.58E-18 | 1.55 | 8.69E-15 |
| GO:0016568 | chromatin modification | 153 | 1.09E-16 | 1.70 | 2.11E-13 |
| GO:0051726 | regulation of cell cycle | 141 | 3.63E-16 | 1.73 | 6.33E-13 |
| GO:0006605 | protein targeting | 97 | 1.30E-15 | 1.91 | 2.53E-12 |
| GO:0006364 | rRNA processing | 62 | 5.61E-15 | 2.20 | 1.07E-11 |
| GO:0070647 | protein modification by small protein conjugation or removal | 79 | 1.85E-14 | 1.99 | 3.52E-11 |
| GO:0016072 | rRNA metabolic process | 62 | 1.90E-14 | 2.17 | 3.60E-11 |
| GO:0006457 | protein folding | 91 | 5.92E-14 | 1.88 | 1.12E-10 |
| GO:0048193 | Golgi vesicle transport | 47 | 6.09E-14 | 2.37 | 1.16E-10 |
| GO:0006796 | phosphate metabolic process | 435 | 8.40E-14 | 1.32 | 1.59E-10 |

**Table S9. 50 most significant biological functions implicated in 1wk kidney H3K27me3 marked genes by DAVID gene ontology analysis**

| **GO number** | **GO Term** | **Count** | **P-Value** | **Fold Enrichment** | **FDR** |
| --- | --- | --- | --- | --- | --- |
| GO:0030182 | neuron differentiation | 162 | 7.45E-48 | 3.33 | 1.37E-44 |
| GO:0007267 | cell-cell signaling | 117 | 3.70E-34 | 3.31 | 6.80E-31 |
| GO:0007389 | pattern specification process | 111 | 5.75E-31 | 3.21 | 1.06E-27 |
| GO:0048666 | neuron development | 112 | 2.15E-30 | 3.15 | 3.96E-27 |
| GO:0006811 | ion transport | 193 | 1.36E-28 | 2.22 | 2.49E-25 |
| GO:0048598 | embryonic morphogenesis | 123 | 5.21E-28 | 2.81 | 9.58E-25 |
| GO:0045165 | cell fate commitment | 71 | 1.75E-26 | 3.96 | 3.22E-23 |
| GO:0007409 | axonogenesis | 75 | 2.78E-26 | 3.78 | 5.11E-23 |
| GO:0000904 | cell morphogenesis involved in differentiation | 87 | 4.25E-26 | 3.37 | 7.82E-23 |
| GO:0031175 | neuron projection development | 88 | 8.62E-26 | 3.31 | 1.59E-22 |
| GO:0003002 | regionalization | 87 | 9.29E-26 | 3.34 | 1.71E-22 |
| GO:0048812 | neuron projection morphogenesis | 77 | 2.97E-25 | 3.59 | 5.47E-22 |
| GO:0006355 | regulation of transcription, DNA-dependent | 310 | 3.57E-25 | 1.74 | 6.57E-22 |
| GO:0051252 | regulation of RNA metabolic process | 313 | 5.59E-25 | 1.73 | 1.03E-21 |
| GO:0048667 | cell morphogenesis involved in neuron differentiation | 78 | 7.02E-25 | 3.52 | 1.29E-21 |
| GO:0030001 | metal ion transport | 134 | 1.05E-24 | 2.49 | 1.92E-21 |
| GO:0048568 | embryonic organ development | 88 | 2.77E-22 | 3.00 | 5.09E-19 |
| GO:0048858 | cell projection morphogenesis | 79 | 3.41E-22 | 3.21 | 6.27E-19 |
| GO:0006812 | cation transport | 142 | 8.22E-22 | 2.26 | 1.51E-18 |
| GO:0045935 | positive regulation of nucleobase, nucleoside, nucleotide and nucleic acid metabolic process | 141 | 8.87E-22 | 2.27 | 1.63E-18 |
| GO:0045893 | positive regulation of transcription, DNA-dependent | 123 | 1.08E-21 | 2.43 | 1.98E-18 |
| GO:0045941 | positive regulation of transcription | 134 | 1.45E-21 | 2.31 | 2.66E-18 |
| GO:0051254 | positive regulation of RNA metabolic process | 123 | 2.09E-21 | 2.41 | 3.84E-18 |
| GO:0045944 | positive regulation of transcription from RNA polymerase II promoter | 111 | 2.16E-21 | 2.54 | 3.96E-18 |
| GO:0010628 | positive regulation of gene expression | 136 | 2.32E-21 | 2.29 | 4.27E-18 |
| GO:0007423 | sensory organ development | 90 | 2.33E-21 | 2.87 | 4.29E-18 |
| GO:0051173 | positive regulation of nitrogen compound metabolic process | 143 | 2.43E-21 | 2.23 | 4.46E-18 |
| GO:0007268 | synaptic transmission | 72 | 2.88E-21 | 3.32 | 5.29E-18 |
| GO:0009891 | positive regulation of biosynthetic process | 148 | 4.78E-21 | 2.18 | 8.78E-18 |
| GO:0032990 | cell part morphogenesis | 79 | 1.09E-20 | 3.06 | 2.00E-17 |
| GO:0010557 | positive regulation of macromolecule biosynthetic process | 142 | 1.44E-20 | 2.20 | 2.65E-17 |
| GO:0031328 | positive regulation of cellular biosynthetic process | 145 | 3.89E-20 | 2.16 | 7.16E-17 |
| GO:0007411 | axon guidance | 50 | 4.33E-20 | 4.19 | 7.96E-17 |
| GO:0006357 | regulation of transcription from RNA polymerase II promoter | 156 | 5.73E-20 | 2.08 | 1.05E-16 |
| GO:0006928 | cell motion | 110 | 5.85E-20 | 2.46 | 1.08E-16 |
| GO:0048562 | embryonic organ morphogenesis | 66 | 6.50E-20 | 3.36 | 1.20E-16 |
| GO:0030900 | forebrain development | 67 | 1.30E-19 | 3.29 | 2.38E-16 |
| GO:0019226 | transmission of nerve impulse | 80 | 2.20E-19 | 2.90 | 4.05E-16 |
| GO:0000902 | cell morphogenesis | 97 | 3.45E-19 | 2.58 | 6.34E-16 |
| GO:0030030 | cell projection organization | 99 | 3.48E-19 | 2.55 | 6.40E-16 |
| GO:0048663 | neuron fate commitment | 33 | 4.68E-19 | 5.76 | 8.61E-16 |
| GO:0007610 | behavior | 115 | 7.85E-19 | 2.33 | 1.44E-15 |
| GO:0010604 | positive regulation of macromolecule metabolic process | 155 | 2.22E-18 | 2.01 | 4.09E-15 |
| GO:0051960 | regulation of nervous system development | 60 | 6.80E-18 | 3.33 | 1.25E-14 |
| GO:0032989 | cellular component morphogenesis | 102 | 1.54E-17 | 2.38 | 2.84E-14 |
| GO:0006813 | potassium ion transport | 62 | 2.41E-17 | 3.18 | 4.44E-14 |
| GO:0050767 | regulation of neurogenesis | 54 | 2.23E-16 | 3.36 | 4.11E-13 |
| GO:0043583 | ear development | 46 | 1.80E-15 | 3.63 | 3.26E-12 |
| GO:0045664 | regulation of neuron differentiation | 45 | 4.23E-15 | 3.62 | 7.76E-12 |
| GO:0015672 | monovalent inorganic cation transport | 88 | 4.66E-15 | 2.38 | 8.58E-12 |

**Table S10. 50 most significant biological functions implicated in 1wk lung H3K27me3 marked genes by DAVID gene ontology analysis**

| **GO number** | **GO Term** | **Count** | **P-Value** | **Fold Enrichment** | **FDR** |
| --- | --- | --- | --- | --- | --- |
| GO:0030182 | neuron differentiation | 173 | 2.39E-56 | 3.57 | 4.39E-53 |
| GO:0007389 | pattern specification process | 120 | 1.53E-37 | 3.48 | 2.81E-34 |
| GO:0007267 | cell-cell signaling | 118 | 5.06E-35 | 3.35 | 9.28E-32 |
| GO:0003002 | regionalization | 98 | 3.68E-34 | 3.77 | 6.76E-31 |
| GO:0048666 | neuron development | 117 | 5.63E-34 | 3.30 | 1.03E-30 |
| GO:0048598 | embryonic morphogenesis | 128 | 3.27E-31 | 2.94 | 6.01E-28 |
| GO:0000904 | cell morphogenesis involved in differentiation | 90 | 2.18E-28 | 3.50 | 4.00E-25 |
| GO:0007409 | axonogenesis | 77 | 5.35E-28 | 3.89 | 9.82E-25 |
| GO:0048812 | neuron projection morphogenesis | 80 | 1.13E-27 | 3.74 | 2.07E-24 |
| GO:0031175 | neuron projection development | 90 | 2.53E-27 | 3.40 | 4.65E-24 |
| GO:0045165 | cell fate commitment | 71 | 1.40E-26 | 3.98 | 2.58E-23 |
| GO:0007423 | sensory organ development | 98 | 1.56E-26 | 3.14 | 2.87E-23 |
| GO:0048667 | cell morphogenesis involved in neuron differentiation | 80 | 1.74E-26 | 3.62 | 3.19E-23 |
| GO:0048858 | cell projection morphogenesis | 84 | 8.68E-26 | 3.42 | 1.59E-22 |
| GO:0006355 | regulation of transcription, DNA-dependent | 310 | 1.90E-25 | 1.74 | 3.49E-22 |
| GO:0048568 | embryonic organ development | 92 | 5.60E-25 | 3.14 | 1.03E-21 |
| GO:0051252 | regulation of RNA metabolic process | 312 | 6.32E-25 | 1.73 | 1.16E-21 |
| GO:0006811 | ion transport | 184 | 8.81E-25 | 2.13 | 1.62E-21 |
| GO:0009952 | anterior/posterior pattern formation | 70 | 1.77E-24 | 3.77 | 3.26E-21 |
| GO:0032990 | cell part morphogenesis | 84 | 4.14E-24 | 3.26 | 7.60E-21 |
| GO:0048562 | embryonic organ morphogenesis | 71 | 1.15E-23 | 3.63 | 2.11E-20 |
| GO:0007268 | synaptic transmission | 74 | 8.95E-23 | 3.42 | 1.64E-19 |
| GO:0000902 | cell morphogenesis | 103 | 1.28E-22 | 2.75 | 2.36E-19 |
| GO:0045944 | positive regulation of transcription from RNA polymerase II promoter | 113 | 1.38E-22 | 2.60 | 2.53E-19 |
| GO:0030030 | cell projection organization | 104 | 5.21E-22 | 2.68 | 9.57E-19 |
| GO:0045893 | positive regulation of transcription, DNA-dependent | 123 | 7.88E-22 | 2.43 | 1.45E-18 |
| GO:0048663 | neuron fate commitment | 35 | 1.44E-21 | 6.13 | 2.65E-18 |
| GO:0051254 | positive regulation of RNA metabolic process | 123 | 1.53E-21 | 2.42 | 2.82E-18 |
| GO:0019226 | transmission of nerve impulse | 83 | 2.44E-21 | 3.02 | 4.48E-18 |
| GO:0045941 | positive regulation of transcription | 133 | 3.09E-21 | 2.31 | 5.67E-18 |
| GO:0001501 | skeletal system development | 95 | 6.55E-21 | 2.75 | 1.20E-17 |
| GO:0030001 | metal ion transport | 126 | 7.32E-21 | 2.35 | 1.34E-17 |
| GO:0010628 | positive regulation of gene expression | 134 | 1.40E-20 | 2.26 | 2.57E-17 |
| GO:0030900 | forebrain development | 68 | 2.15E-20 | 3.35 | 3.95E-17 |
| GO:0032989 | cellular component morphogenesis | 107 | 3.74E-20 | 2.51 | 6.87E-17 |
| GO:0045935 | positive regulation of nucleobase, nucleoside, nucleotide and nucleic acid metabolic process | 137 | 4.13E-20 | 2.21 | 7.58E-17 |
| GO:0051960 | regulation of nervous system development | 63 | 4.14E-20 | 3.51 | 7.60E-17 |
| GO:0006928 | cell motion | 110 | 4.43E-20 | 2.47 | 8.13E-17 |
| GO:0009891 | positive regulation of biosynthetic process | 145 | 6.75E-20 | 2.14 | 1.24E-16 |
| GO:0051173 | positive regulation of nitrogen compound metabolic process | 139 | 1.03E-19 | 2.18 | 1.89E-16 |
| GO:0006357 | regulation of transcription from RNA polymerase II promoter | 155 | 1.04E-19 | 2.07 | 1.90E-16 |
| GO:0060284 | regulation of cell development | 65 | 1.25E-19 | 3.37 | 2.29E-16 |
| GO:0050767 | regulation of neurogenesis | 58 | 2.37E-19 | 3.62 | 4.35E-16 |
| GO:0007411 | axon guidance | 49 | 2.70E-19 | 4.12 | 4.95E-16 |
| GO:0031328 | positive regulation of cellular biosynthetic process | 142 | 5.27E-19 | 2.12 | 9.68E-16 |
| GO:0010557 | positive regulation of macromolecule biosynthetic process | 138 | 5.68E-19 | 2.14 | 1.04E-15 |
| GO:0007610 | behavior | 114 | 1.76E-18 | 2.32 | 3.22E-15 |
| GO:0006812 | cation transport | 133 | 5.44E-18 | 2.13 | 9.99E-15 |
| GO:0045664 | regulation of neuron differentiation | 48 | 1.42E-17 | 3.88 | 2.60E-14 |
| GO:0043583 | ear development | 47 | 2.22E-16 | 3.72 | 4.11E-13 |

**Table S11. 50 most significant biological functions implicated in 4wk kidney bivalent genes by DAVID gene ontology analysis**

| **GO number** | **GO Term** | **Count** | **P-Value** | **Fold Enrichment** | **FDR** |
| --- | --- | --- | --- | --- | --- |
| GO:0055065 | metal ion homeostasis | 18 | 1.46E-09 | 6.61 | 2.53E-06 |
| GO:0055080 | cation homeostasis | 23 | 1.85E-09 | 4.86 | 3.20E-06 |
| GO:0006875 | cellular metal ion homeostasis | 17 | 4.64E-09 | 6.61 | 8.04E-06 |
| GO:0050801 | ion homeostasis | 28 | 8.69E-09 | 3.72 | 1.51E-05 |
| GO:0055074 | calcium ion homeostasis | 16 | 1.71E-08 | 6.55 | 2.96E-05 |
| GO:0048878 | chemical homeostasis | 31 | 1.81E-08 | 3.30 | 3.13E-05 |
| GO:0001655 | urogenital system development | 19 | 3.50E-08 | 5.06 | 6.07E-05 |
| GO:0006874 | cellular calcium ion homeostasis | 15 | 7.22E-08 | 6.41 | 1.25E-04 |
| GO:0007389 | pattern specification process | 26 | 7.77E-08 | 3.56 | 1.35E-04 |
| GO:0003002 | regionalization | 22 | 1.42E-07 | 4.00 | 2.45E-04 |
| GO:0030003 | cellular cation homeostasis | 18 | 3.52E-07 | 4.61 | 6.10E-04 |
| GO:0055082 | cellular chemical homeostasis | 24 | 4.09E-07 | 3.48 | 7.09E-04 |
| GO:0001822 | kidney development | 15 | 5.68E-07 | 5.45 | 9.84E-04 |
| GO:0006873 | cellular ion homeostasis | 23 | 9.95E-07 | 3.43 | 0.0017236 |
| GO:0055066 | di-, tri-valent inorganic cation homeostasis | 17 | 1.03E-06 | 4.53 | 0.0017822 |
| GO:0030005 | cellular di-, tri-valent inorganic cation homeostasis | 16 | 1.71E-06 | 4.64 | 0.0029664 |
| GO:0009952 | anterior/posterior pattern formation | 17 | 1.93E-06 | 4.32 | 0.0033374 |
| GO:0007200 | activation of phospholipase C activity by G-protein coupled receptor protein signaling pathway coupled to IP3 second messenger | 8 | 2.20E-06 | 12.4 | 0.003808 |
| GO:0042592 | homeostatic process | 36 | 2.59E-06 | 2.40 | 0.0044871 |
| GO:0010863 | positive regulation of phospholipase C activity | 8 | 2.94E-06 | 11.97 | 0.005097 |
| GO:0007202 | activation of phospholipase C activity | 8 | 2.94E-06 | 11.97 | 0.005097 |
| GO:0010518 | positive regulation of phospholipase activity | 8 | 2.94E-06 | 11.97 | 0.005097 |
| GO:0042127 | regulation of cell proliferation | 34 | 3.13E-06 | 2.46 | 0.0054224 |
| GO:0048514 | blood vessel morphogenesis | 19 | 3.37E-06 | 3.73 | 0.0058359 |
| GO:0043085 | positive regulation of catalytic activity | 22 | 3.69E-06 | 3.28 | 0.0063933 |
| GO:0060193 | positive regulation of lipase activity | 8 | 3.88E-06 | 11.53 | 0.0067306 |
| GO:0044093 | positive regulation of molecular function | 24 | 4.03E-06 | 3.05 | 0.0069782 |
| GO:0001501 | skeletal system development | 23 | 4.25E-06 | 3.14 | 0.007359 |
| GO:0048754 | branching morphogenesis of a tube | 13 | 4.26E-06 | 5.44 | 0.0073811 |
| GO:0035295 | tube development | 22 | 4.42E-06 | 3.24 | 0.0076512 |
| GO:0051216 | cartilage development | 12 | 4.45E-06 | 5.98 | 0.0077169 |
| GO:0010517 | regulation of phospholipase activity | 8 | 5.07E-06 | 11.12 | 0.0087781 |
| GO:0007267 | cell-cell signaling | 23 | 5.63E-06 | 3.08 | 0.0097643 |
| GO:0048015 | phosphoinositide-mediated signaling | 9 | 6.42E-06 | 8.76 | 0.0111209 |
| GO:0001944 | vasculature development | 21 | 6.86E-06 | 3.27 | 0.0118894 |
| GO:0006928 | cell motion | 26 | 8.77E-06 | 2.75 | 0.0151888 |
| GO:0060191 | regulation of lipase activity | 8 | 1.32E-05 | 9.73 | 0.0228443 |
| GO:0016477 | cell migration | 20 | 1.35E-05 | 3.24 | 0.023432 |
| GO:0001656 | metanephros development | 10 | 1.50E-05 | 6.71 | 0.026074 |
| GO:0001568 | blood vessel development | 20 | 1.71E-05 | 3.19 | 0.0296409 |
| GO:0001763 | morphogenesis of a branching structure | 14 | 1.88E-05 | 4.36 | 0.0326471 |
| GO:0019725 | cellular homeostasis | 24 | 2.57E-05 | 2.72 | 0.0445064 |
| GO:0035239 | tube morphogenesis | 16 | 3.38E-05 | 3.64 | 0.0585565 |
| GO:0030182 | neuron differentiation | 26 | 3.59E-05 | 2.53 | 0.0622686 |
| GO:0001525 | angiogenesis | 14 | 3.66E-05 | 4.09 | 0.0633332 |
| GO:0048732 | gland development | 17 | 4.76E-05 | 3.35 | 0.0824232 |
| GO:0003006 | reproductive developmental process | 20 | 5.06E-05 | 2.94 | 0.0877283 |
| GO:0022612 | gland morphogenesis | 11 | 5.47E-05 | 5.09 | 0.0948197 |
| GO:0048870 | cell motility | 20 | 1.34E-04 | 2.74 | 0.2323825 |
| GO:0051674 | localization of cell | 20 | 1.34E-04 | 2.74 | 0.2323825 |

**Table S12. 50 most significant biological functions implicated in 4wk lung bivalent genes by DAVID gene ontology analysis**

| **GO number** | **GO Term** | **Count** | **P-Value** | **Fold Enrichment** | **FDR** |
| --- | --- | --- | --- | --- | --- |
| GO:0035295 | tube development | 43 | 2.20E-18 | 5.14 | 3.84E-15 |
| GO:0001944 | vasculature development | 37 | 1.86E-14 | 4.67 | 3.26E-11 |
| GO:0001568 | blood vessel development | 36 | 4.92E-14 | 4.66 | 8.61E-11 |
| GO:0048514 | blood vessel morphogenesis | 32 | 1.31E-13 | 5.10 | 2.29E-10 |
| GO:0030324 | lung development | 23 | 4.04E-12 | 6.54 | 7.06E-09 |
| GO:0030323 | respiratory tube development | 23 | 5.88E-12 | 6.43 | 1.03E-08 |
| GO:0035239 | tube morphogenesis | 27 | 2.55E-11 | 4.98 | 4.47E-08 |
| GO:0060541 | respiratory system development | 23 | 4.05E-11 | 5.86 | 7.09E-08 |
| GO:0048754 | branching morphogenesis of a tube | 20 | 6.91E-11 | 6.79 | 1.21E-07 |
| GO:0060429 | epithelium development | 33 | 1.21E-10 | 3.84 | 2.11E-07 |
| GO:0001763 | morphogenesis of a branching structure | 22 | 3.26E-10 | 5.56 | 5.71E-07 |
| GO:0006928 | cell motion | 37 | 1.51E-09 | 3.18 | 2.64E-06 |
| GO:0048568 | embryonic organ development | 29 | 2.61E-09 | 3.80 | 4.57E-06 |
| GO:0043009 | chordate embryonic development | 39 | 5.37E-09 | 2.92 | 9.40E-06 |
| GO:0002009 | morphogenesis of an epithelium | 24 | 5.39E-09 | 4.38 | 9.43E-06 |
| GO:0042127 | regulation of cell proliferation | 45 | 6.66E-09 | 2.64 | 1.16E-05 |
| GO:0007389 | pattern specification process | 31 | 6.72E-09 | 3.44 | 1.18E-05 |
| GO:0009792 | embryonic development ending in birth or egg hatching | 39 | 6.97E-09 | 2.89 | 1.22E-05 |
| GO:0006357 | regulation of transcription from RNA polymerase II promoter | 48 | 1.76E-08 | 2.46 | 3.08E-05 |
| GO:0008284 | positive regulation of cell proliferation | 30 | 2.58E-08 | 3.33 | 4.52E-05 |
| GO:0006355 | regulation of transcription, DNA-dependent | 85 | 3.32E-08 | 1.83 | 5.80E-05 |
| GO:0048729 | tissue morphogenesis | 27 | 3.53E-08 | 3.58 | 6.17E-05 |
| GO:0007507 | heart development | 26 | 3.88E-08 | 3.68 | 6.79E-05 |
| GO:0051252 | regulation of RNA metabolic process | 85 | 6.59E-08 | 1.80 | 1.15E-04 |
| GO:0060562 | epithelial tube morphogenesis | 18 | 6.70E-08 | 5.12 | 1.17E-04 |
| GO:0045449 | regulation of transcription | 114 | 7.11E-08 | 1.61 | 1.24E-04 |
| GO:0010628 | positive regulation of gene expression | 40 | 9.35E-08 | 2.59 | 1.64E-04 |
| GO:0001501 | skeletal system development | 29 | 1.03E-07 | 3.21 | 1.80E-04 |
| GO:0048598 | embryonic morphogenesis | 33 | 1.22E-07 | 2.90 | 2.13E-04 |
| GO:0007167 | enzyme linked receptor protein signaling pathway | 28 | 1.52E-07 | 3.24 | 2.67E-04 |
| GO:0016477 | cell migration | 26 | 1.64E-07 | 3.42 | 2.88E-04 |
| GO:0006350 | transcription | 95 | 1.75E-07 | 1.69 | 3.07E-04 |
| GO:0001525 | angiogenesis | 19 | 1.92E-07 | 4.51 | 3.36E-04 |
| GO:0003006 | reproductive developmental process | 27 | 2.81E-07 | 3.23 | 4.92E-04 |
| GO:0010604 | positive regulation of macromolecule metabolic process | 45 | 7.20E-07 | 2.24 | 0.0012607 |
| GO:0009891 | positive regulation of biosynthetic process | 41 | 1.05E-06 | 2.32 | 0.0018344 |
| GO:0045941 | positive regulation of transcription | 37 | 1.06E-06 | 2.46 | 0.0018533 |
| GO:0048732 | gland development | 22 | 1.11E-06 | 3.52 | 0.0019435 |
| GO:0003002 | regionalization | 23 | 1.14E-06 | 3.39 | 0.0020017 |
| GO:0010557 | positive regulation of macromolecule biosynthetic process | 39 | 2.05E-06 | 2.32 | 0.0035823 |
| GO:0045935 | positive regulation of nucleobase, nucleoside, nucleotide and nucleic acid metabolic process | 38 | 2.13E-06 | 2.35 | 0.0037285 |
| GO:0007411 | axon guidance | 15 | 2.30E-06 | 4.83 | 0.0040167 |
| GO:0016481 | negative regulation of transcription | 31 | 2.50E-06 | 2.63 | 0.004366 |
| GO:0045893 | positive regulation of transcription, DNA-dependent | 33 | 3.15E-06 | 2.50 | 0.0055159 |
| GO:0045934 | negative regulation of nucleobase, nucleoside, nucleotide and nucleic acid metabolic process | 32 | 3.31E-06 | 2.54 | 0.0057959 |
| GO:0000904 | cell morphogenesis involved in differentiation | 22 | 3.64E-06 | 3.27 | 0.0063624 |
| GO:0051254 | positive regulation of RNA metabolic process | 33 | 3.67E-06 | 2.48 | 0.0064205 |
| GO:0030182 | neuron differentiation | 32 | 3.67E-06 | 2.53 | 0.0064274 |
| GO:0048870 | cell motility | 26 | 3.78E-06 | 2.89 | 0.0066074 |
| GO:0051674 | localization of cell | 26 | 3.78E-06 | 2.89 | 0.0066074 |

**Table S13. 50 most significant biological functions implicated in 4wk kidney H3K4me3 genes by DAVID gene ontology analysis**

| **GO number** | **GO Term** | **Count** | **P-Value** | **Fold Enrichment** | **FDR** |
| --- | --- | --- | --- | --- | --- |
| GO:0006396 | RNA processing | 358 | 2.86E-68 | 1.97 | 5.43E-65 |
| GO:0045184 | establishment of protein localization | 485 | 1.16E-65 | 1.77 | 2.20E-62 |
| GO:0008104 | protein localization | 540 | 1.23E-65 | 1.72 | 2.33E-62 |
| GO:0015031 | protein transport | 482 | 1.39E-65 | 1.78 | 2.64E-62 |
| GO:0009057 | macromolecule catabolic process | 452 | 1.70E-47 | 1.66 | 3.23E-44 |
| GO:0044265 | cellular macromolecule catabolic process | 426 | 7.46E-47 | 1.68 | 1.42E-43 |
| GO:0046907 | apoptosis | 322 | 6.03E-45 | 1.79 | 1.15E-41 |
| GO:0030163 | protein catabolic process | 393 | 6.35E-45 | 1.70 | 1.21E-41 |
| GO:0006974 | response to DNA damage stimulus | 232 | 3.17E-42 | 1.94 | 6.02E-39 |
| GO:0019941 | modification-dependent protein catabolic process | 360 | 1.59E-41 | 1.70 | 3.01E-38 |
| GO:0043632 | modification-dependent macromolecule catabolic process | 360 | 1.59E-41 | 1.70 | 3.01E-38 |
| GO:0044257 | cellular protein catabolic process | 376 | 1.87E-41 | 1.68 | 3.56E-38 |
| GO:0051603 | proteolysis involved in cellular protein catabolic process | 374 | 2.83E-41 | 1.68 | 5.37E-38 |
| GO:0033554 | cellular response to stress | 297 | 3.68E-39 | 1.76 | 7.00E-36 |
| GO:0016071 | mRNA metabolic process | 237 | 3.92E-39 | 1.88 | 7.46E-36 |
| GO:0034660 | ncRNA metabolic process | 172 | 5.07E-37 | 2.04 | 9.64E-34 |
| GO:0006259 | DNA metabolic process | 302 | 1.91E-36 | 1.72 | 3.62E-33 |
| GO:0006281 | DNA repair | 184 | 2.43E-36 | 1.99 | 4.61E-33 |
| GO:0007049 | cell cycle | 402 | 1.42E-34 | 1.58 | 2.69E-31 |
| GO:0006397 | mRNA processing | 206 | 2.87E-34 | 1.89 | 5.45E-31 |
| GO:0006412 | translation | 239 | 9.85E-34 | 1.80 | 1.87E-30 |
| GO:0008380 | RNA splicing | 167 | 2.87E-33 | 1.99 | 5.45E-30 |
| GO:0034470 | ncRNA processing | 139 | 3.40E-33 | 2.11 | 6.46E-30 |
| GO:0006350 | transcription | 966 | 8.04E-32 | 1.31 | 1.53E-28 |
| GO:0006886 | intracellular protein transport | 204 | 1.50E-27 | 1.77 | 2.85E-24 |
| GO:0070727 | cellular macromolecule localization | 218 | 2.28E-27 | 1.74 | 4.34E-24 |
| GO:0022613 | ribonucleoprotein complex biogenesis | 119 | 2.37E-27 | 2.08 | 4.50E-24 |
| GO:0034613 | cellular protein localization | 216 | 7.06E-27 | 1.73 | 1.34E-23 |
| GO:0000278 | mitotic cell cycle | 180 | 3.14E-24 | 1.77 | 5.98E-21 |
| GO:0048285 | organelle fission | 151 | 3.01E-23 | 1.84 | 5.71E-20 |
| GO:0016568 | chromatin modification | 173 | 8.20E-23 | 1.76 | 1.56E-19 |
| GO:0000280 | nuclear division | 146 | 1.09E-22 | 1.84 | 2.07E-19 |
| GO:0007067 | mitosis | 146 | 1.09E-22 | 1.84 | 2.07E-19 |
| GO:0051276 | chromosome organization | 264 | 3.06E-22 | 1.57 | 5.82E-19 |
| GO:0000087 | M phase of mitotic cell cycle | 147 | 8.29E-22 | 1.82 | 1.58E-18 |
| GO:0022402 | cell cycle process | 257 | 9.71E-22 | 1.57 | 1.85E-18 |
| GO:0006399 | tRNA metabolic process | 97 | 1.43E-21 | 2.06 | 2.71E-18 |
| GO:0051301 | cell division | 196 | 1.52E-21 | 1.67 | 2.90E-18 |
| GO:0042254 | ribosome biogenesis | 96 | 2.93E-21 | 2.06 | 5.58E-18 |
| GO:0022403 | cell cycle phase | 219 | 2.79E-20 | 1.60 | 5.30E-17 |
| GO:0045449 | regulation of transcription | 1114 | 9.25E-19 | 1.20 | 1.76E-15 |
| GO:0006325 | chromatin organization | 208 | 1.76E-18 | 1.58 | 3.34E-15 |
| GO:0000279 | M phase | 190 | 4.15E-18 | 1.61 | 7.89E-15 |
| GO:0006364 | rRNA processing | 67 | 1.33E-17 | 2.17 | 2.53E-14 |
| GO:0016072 | rRNA metabolic process | 67 | 6.49E-17 | 2.14 | 2.11E-13 |
| GO:0051726 | regulation of cell cycle | 148 | 7.56E-16 | 1.66 | 1.48E-12 |
| GO:0048193 | Golgi vesicle transport | 49 | 1.67E-14 | 2.26 | 3.17E-11 |
| GO:0006915 | apoptosis | 275 | 1.85E-14 | 1.42 | 3.52E-11 |
| GO:0006457 | protein folding | 96 | 2.65E-14 | 1.81 | 5.04E-11 |
| GO:0016192 | vesicle-mediated transport | 275 | 2.65E-14 | 1.42 | 5.04E-11 |

**Table S14. 50 most significant biological functions implicated in 4wk lung H3K4me3 genes by DAVID gene ontology analysis**

| **GO number** | **GO Term** | **Count** | **P-Value** | **Fold Enrichment** | **FDR** |
| --- | --- | --- | --- | --- | --- |
| GO:0045184 | establishment of protein localization | 427 | 1.37E-56 | 1.84 | 2.60E-53 |
| GO:0015031 | protein transport | 424 | 2.74E-56 | 1.84 | 5.19E-53 |
| GO:0008104 | protein localization | 472 | 1.89E-55 | 1.78 | 3.58E-52 |
| GO:0006396 | RNA processing | 312 | 2.31E-54 | 2.02 | 4.37E-51 |
| GO:0009057 | macromolecule catabolic process | 399 | 1.48E-42 | 1.73 | 2.80E-39 |
| GO:0044265 | cellular macromolecule catabolic process | 377 | 3.04E-42 | 1.75 | 5.76E-39 |
| GO:0030163 | protein catabolic process | 348 | 1.95E-40 | 1.77 | 3.69E-37 |
| GO:0046907 | intracellular transport | 286 | 4.65E-40 | 1.88 | 8.80E-37 |
| GO:0044257 | cellular protein catabolic process | 333 | 1.70E-37 | 1.76 | 3.21E-34 |
| GO:0051603 | proteolysis involved in cellular protein catabolic process | 331 | 3.22E-37 | 1.76 | 6.10E-34 |
| GO:0033554 | cellular response to stress | 267 | 6.13E-37 | 1.87 | 1.16E-33 |
| GO:0016071 | mRNA metabolic process | 214 | 1.80E-36 | 2.01 | 3.41E-33 |
| GO:0006974 | response to DNA damage stimulus | 206 | 2.00E-36 | 2.03 | 3.79E-33 |
| GO:0019941 | modification-dependent protein catabolic process | 315 | 1.83E-35 | 1.76 | 3.47E-32 |
| GO:0043632 | modification-dependent macromolecule catabolic process | 315 | 1.83E-35 | 1.76 | 3.47E-32 |
| GO:0006259 | DNA metabolic process | 268 | 6.17E-33 | 1.80 | 1.17E-29 |
| GO:0006412 | translation | 216 | 3.10E-32 | 1.92 | 5.88E-29 |
| GO:0006350 | transcription | 850 | 3.31E-32 | 1.36 | 6.27E-29 |
| GO:0006397 | mRNA processing | 186 | 6.39E-32 | 2.01 | 1.21E-28 |
| GO:0007049 | cell cycle | 354 | 2.60E-31 | 1.64 | 4.92E-28 |
| GO:0006281 | DNA repair | 162 | 4.29E-30 | 2.07 | 8.12E-27 |
| GO:0008380 | RNA splicing | 149 | 6.11E-29 | 2.10 | 1.16E-25 |
| GO:0070727 | cellular macromolecule localization | 201 | 8.28E-29 | 1.89 | 1.57E-25 |
| GO:0034613 | cellular protein localization | 199 | 3.05E-28 | 1.89 | 5.77E-25 |
| GO:0006886 | intracellular protein transport | 186 | 1.90E-27 | 1.91 | 3.60E-24 |
| GO:0034660 | ncRNA metabolic process | 145 | 8.41E-26 | 2.03 | 1.59E-22 |
| GO:0022613 | ribonucleoprotein complex biogenesis | 106 | 3.04E-23 | 2.19 | 5.76E-20 |
| GO:0045449 | regulation of transcription | 985 | 5.71E-22 | 1.25 | 1.08E-18 |
| GO:0034470 | ncRNA processing | 115 | 2.10E-21 | 2.06 | 3.98E-18 |
| GO:0051276 | chromosome organization | 232 | 4.61E-20 | 1.63 | 8.72E-17 |
| GO:0000278 | mitotic cell cycle | 156 | 9.70E-20 | 1.81 | 1.84E-16 |
| GO:0016568 | chromatin modification | 152 | 1.07E-19 | 1.82 | 2.02E-16 |
| GO:0000280 | nuclear division | 128 | 4.06E-19 | 1.91 | 7.68E-16 |
| GO:0007067 | mitosis | 128 | 4.06E-19 | 1.91 | 7.68E-16 |
| GO:0048285 | organelle fission | 131 | 8.10E-19 | 1.88 | 1.53E-15 |
| GO:0000087 | M phase of mitotic cell cycle | 129 | 1.53E-18 | 1.88 | 2.89E-15 |
| GO:0051301 | cell division | 170 | 6.22E-18 | 1.71 | 1.18E-14 |
| GO:0022402 | cell cycle process | 221 | 1.02E-17 | 1.59 | 1.94E-14 |
| GO:0042254 | ribosome biogenesis | 84 | 3.98E-17 | 2.12 | 7.54E-14 |
| GO:0022403 | cell cycle phase | 188 | 3.56E-16 | 1.62 | 6.33E-13 |
| GO:0006325 | chromatin organization | 180 | 2.72E-15 | 1.62 | 5.25E-12 |
| GO:0000279 | M phase | 164 | 9.41E-15 | 1.64 | 1.79E-11 |
| GO:0006399 | tRNA metabolic process | 81 | 1.34E-14 | 2.03 | 2.54E-11 |
| GO:0006457 | protein folding | 87 | 8.54E-14 | 1.94 | 1.62E-10 |
| GO:0006091 | generation of precursor metabolites and energy | 150 | 3.56E-13 | 1.63 | 6.74E-10 |
| GO:0070647 | protein modification by small protein conjugation or removal | 74 | 3.88E-13 | 2.02 | 7.35E-10 |
| GO:0006260 | DNA replication | 98 | 6.10E-13 | 1.83 | 1.15E-09 |
| GO:0006605 | protein targeting | 88 | 1.24E-12 | 1.87 | 2.35E-09 |
| GO:0022900 | electron transport chain | 77 | 2.01E-12 | 1.95 | 3.80E-09 |
| GO:0006913 | nucleocytoplasmic transport | 68 | 5.48E-12 | 2.01 | 1.04E-08 |

**Table S15. 50 most significant biological functions implicated in 4wk kidney H3K27me3 genes by DAVID gene ontology analysis**

| **GO number** | **GO Term** | **Count** | **P-Value** | **Fold Enrichment** | **FDR** |
| --- | --- | --- | --- | --- | --- |
| GO:0030182 | neuron differentiation | 169 | 1.83E-46 | 3.12 | 3.37E-43 |
| GO:0007267 | cell-cell signaling | 126 | 6.33E-36 | 3.21 | 1.17E-32 |
| GO:0045165 | cell fate commitment | 78 | 1.91E-29 | 3.91 | 3.53E-26 |
| GO:0006811 | ion transport | 207 | 9.31E-29 | 2.14 | 1.72E-25 |
| GO:0048598 | embryonic morphogenesis | 131 | 2.80E-28 | 2.69 | 5.16E-25 |
| GO:0048666 | neuron development | 115 | 3.33E-28 | 2.91 | 6.13E-25 |
| GO:0007389 | pattern specification process | 112 | 1.54E-27 | 2.91 | 2.84E-24 |
| GO:0030001 | metal ion transport | 146 | 2.53E-26 | 2.44 | 4.66E-23 |
| GO:0000904 | cell morphogenesis involved in differentiation | 89 | 3.26E-24 | 3.10 | 6.02E-21 |
| GO:0007423 | sensory organ development | 100 | 4.03E-24 | 2.87 | 7.43E-21 |
| GO:0048568 | embryonic organ development | 95 | 2.01E-23 | 2.91 | 3.71E-20 |
| GO:0007268 | synaptic transmission | 79 | 2.19E-23 | 3.27 | 4.03E-20 |
| GO:0048667 | cell morphogenesis involved in neuron differentiation | 80 | 2.38E-23 | 3.24 | 4.40E-20 |
| GO:0007409 | axonogenesis | 74 | 1.16E-22 | 3.35 | 2.14E-19 |
| GO:0048812 | neuron projection morphogenesis | 77 | 2.41E-22 | 3.23 | 4.44E-19 |
| GO:0006812 | cation transport | 153 | 2.64E-22 | 2.19 | 4.87E-19 |
| GO:0007610 | behavior | 130 | 3.07E-22 | 2.37 | 5.66E-19 |
| GO:0031175 | neuron projection development | 87 | 6.36E-22 | 2.94 | 1.17E-18 |
| GO:0003002 | regionalization | 86 | 6.50E-22 | 2.96 | 1.20E-18 |
| GO:0048562 | embryonic organ morphogenesis | 71 | 6.83E-21 | 3.25 | 1.26E-17 |
| GO:0006355 | regulation of transcription, DNA-dependent | 321 | 8.25E-21 | 1.62 | 1.52E-17 |
| GO:0051252 | regulation of RNA metabolic process | 324 | 1.46E-20 | 1.61 | 2.70E-17 |
| GO:0006813 | potassium ion transport | 70 | 2.25E-20 | 3.23 | 4.15E-17 |
| GO:0045944 | positive regulation of transcription from RNA polymerase II promoter | 116 | 2.83E-20 | 2.39 | 5.22E-17 |
| GO:0044057 | regulation of system process | 80 | 3.93E-20 | 2.94 | 7.25E-17 |
| GO:0019226 | transmission of nerve impulse | 86 | 4.15E-20 | 2.81 | 7.65E-17 |
| GO:0048663 | neuron fate commitment | 35 | 5.12E-20 | 5.49 | 9.44E-17 |
| GO:0048858 | cell projection morphogenesis | 80 | 5.60E-20 | 2.92 | 1.03E-16 |
| GO:0030900 | forebrain development | 71 | 8.14E-20 | 3.14 | 1.50E-16 |
| GO:0006357 | regulation of transcription from RNA polymerase II promoter | 165 | 5.21E-19 | 1.98 | 9.60E-16 |
| GO:0045893 | positive regulation of transcription, DNA-dependent | 125 | 8.98E-19 | 2.22 | 1.66E-15 |
| GO:0032990 | cell part morphogenesis | 80 | 1.65E-18 | 2.78 | 3.04E-15 |
| GO:0051254 | positive regulation of RNA metabolic process | 125 | 1.70E-18 | 2.20 | 3.13E-15 |
| GO:0007155 | cell adhesion | 153 | 1.93E-18 | 2.01 | 3.55E-15 |
| GO:0022610 | biological adhesion | 153 | 2.30E-18 | 2.01 | 4.24E-15 |
| GO:0007411 | axon guidance | 50 | 3.98E-18 | 3.76 | 7.34E-15 |
| GO:0010557 | positive regulation of macromolecule biosynthetic process | 146 | 4.83E-18 | 2.03 | 8.91E-15 |
| GO:0045935 | positive regulation of nucleobase, nucleoside, nucleotide and nucleic acid metabolic process | 142 | 5.31E-18 | 2.05 | 9.80E-15 |
| GO:0009891 | positive regulation of biosynthetic process | 151 | 5.91E-18 | 2.00 | 1.09E-14 |
| GO:0006928 | cell motion | 113 | 6.60E-18 | 2.27 | 1.22E-14 |
| GO:0031328 | positive regulation of cellular biosynthetic process | 149 | 1.51E-17 | 1.99 | 2.78E-14 |
| GO:0045941 | positive regulation of transcription | 134 | 1.55E-17 | 2.08 | 2.86E-14 |
| GO:0010628 | positive regulation of gene expression | 136 | 2.62E-17 | 2.06 | 4.83E-14 |
| GO:0051173 | positive regulation of nitrogen compound metabolic process | 143 | 3.72E-17 | 2.01 | 6.86E-14 |
| GO:0000902 | cell morphogenesis | 99 | 4.71E-17 | 2.36 | 8.68E-14 |
| GO:0001501 | skeletal system development | 93 | 2.35E-16 | 2.41 | 4.11E-13 |
| GO:0048729 | tissue morphogenesis | 82 | 3.94E-16 | 2.54 | 8.22E-13 |
| GO:0015672 | monovalent inorganic cation transport | 96 | 4.34E-16 | 2.34 | 8.22E-13 |
| GO:0032989 | cellular component morphogenesis | 106 | 5.50E-16 | 2.23 | 1.02E-12 |
| GO:0051960 | regulation of nervous system development | 60 | 1.33E-15 | 2.99 | 2.45E-12 |

**Table S16. 50 most significant biological functions implicated in 4wk lung H3K27me3 genes by DAVID gene ontology analysis**

| **GO number** | **GO Term** | **Count** | **P-Value** | **Fold Enrichment** | **FDR** |
| --- | --- | --- | --- | --- | --- |
| GO:0030182 | neuron differentiation | 179 | 3.33E-48 | 3.04 | 6.17E-45 |
| GO:0007389 | pattern specification process | 129 | 2.36E-35 | 3.08 | 4.38E-32 |
| GO:0048598 | embryonic morphogenesis | 145 | 8.45E-33 | 2.74 | 1.57E-29 |
| GO:0048666 | neuron development | 127 | 1.68E-32 | 2.95 | 3.11E-29 |
| GO:0007267 | cell-cell signaling | 126 | 3.41E-32 | 2.95 | 6.32E-29 |
| GO:0045165 | cell fate commitment | 82 | 2.34E-30 | 3.78 | 4.34E-27 |
| GO:0003002 | regionalization | 102 | 3.86E-30 | 3.23 | 7.16E-27 |
| GO:0000904 | cell morphogenesis involved in differentiation | 100 | 4.22E-29 | 3.20 | 7.83E-26 |
| GO:0048568 | embryonic organ development | 108 | 4.98E-29 | 3.04 | 9.24E-26 |
| GO:0048812 | neuron projection morphogenesis | 88 | 5.98E-28 | 3.39 | 1.11E-24 |
| GO:0031175 | neuron projection development | 100 | 6.73E-28 | 3.11 | 1.25E-24 |
| GO:0006355 | regulation of transcription, DNA-dependent | 364 | 2.19E-27 | 1.68 | 4.07E-24 |
| GO:0048667 | cell morphogenesis involved in neuron differentiation | 89 | 2.28E-27 | 3.32 | 4.23E-24 |
| GO:0007409 | axonogenesis | 83 | 4.39E-27 | 3.45 | 8.15E-24 |
| GO:0051252 | regulation of RNA metabolic process | 367 | 5.70E-27 | 1.67 | 1.06E-23 |
| GO:0000902 | cell morphogenesis | 119 | 7.69E-25 | 2.61 | 1.43E-21 |
| GO:0048562 | embryonic organ morphogenesis | 79 | 1.79E-24 | 3.33 | 3.32E-21 |
| GO:0006357 | regulation of transcription from RNA polymerase II promoter | 188 | 2.18E-24 | 2.07 | 4.04E-21 |
| GO:0048858 | cell projection morphogenesis | 90 | 4.61E-24 | 3.02 | 8.55E-21 |
| GO:0045944 | positive regulation of transcription from RNA polymerase II promoter | 128 | 2.79E-23 | 2.42 | 5.18E-20 |
| GO:0009952 | anterior/posterior pattern formation | 75 | 3.13E-23 | 3.32 | 5.80E-20 |
| GO:0006811 | ion transport | 205 | 4.19E-23 | 1.95 | 7.78E-20 |
| GO:0045893 | positive regulation of transcription, DNA-dependent | 141 | 4.80E-23 | 2.30 | 8.91E-20 |
| GO:0051254 | positive regulation of RNA metabolic process | 141 | 1.03E-22 | 2.28 | 1.92E-19 |
| GO:0001501 | skeletal system development | 109 | 1.58E-22 | 2.59 | 2.93E-19 |
| GO:0010628 | positive regulation of gene expression | 156 | 1.64E-22 | 2.17 | 3.04E-19 |
| GO:0030030 | cell projection organization | 117 | 2.25E-22 | 2.49 | 4.17E-19 |
| GO:0032990 | cell part morphogenesis | 90 | 2.68E-22 | 2.88 | 4.96E-19 |
| GO:0006928 | cell motion | 128 | 3.40E-22 | 2.36 | 6.30E-19 |
| GO:0009891 | positive regulation of biosynthetic process | 170 | 4.35E-22 | 2.07 | 8.07E-19 |
| GO:0032989 | cellular component morphogenesis | 124 | 4.62E-22 | 2.40 | 8.57E-19 |
| GO:0045941 | positive regulation of transcription | 152 | 5.37E-22 | 2.17 | 9.96E-19 |
| GO:0007423 | sensory organ development | 101 | 6.14E-22 | 2.66 | 1.14E-18 |
| GO:0048732 | gland development | 85 | 1.19E-21 | 2.93 | 2.21E-18 |
| GO:0045935 | positive regulation of nucleobase, nucleoside, nucleotide and nucleic acid metabolic process | 158 | 2.84E-21 | 2.10 | 5.27E-18 |
| GO:0010557 | positive regulation of macromolecule biosynthetic process | 162 | 3.83E-21 | 2.07 | 7.10E-18 |
| GO:0031328 | positive regulation of cellular biosynthetic process | 166 | 7.45E-21 | 2.04 | 1.38E-17 |
| GO:0042127 | regulation of cell proliferation | 163 | 7.63E-21 | 2.05 | 1.41E-17 |
| GO:0007411 | axon guidance | 55 | 1.10E-20 | 3.81 | 2.05E-17 |
| GO:0051173 | positive regulation of nitrogen compound metabolic process | 160 | 1.17E-20 | 2.06 | 2.17E-17 |
| GO:0060284 | regulation of cell development | 72 | 7.17E-20 | 3.07 | 1.33E-16 |
| GO:0030001 | metal ion transport | 140 | 7.26E-20 | 2.15 | 1.35E-16 |
| GO:0035295 | tube development | 99 | 7.99E-20 | 2.54 | 1.48E-16 |
| GO:0008284 | positive regulation of cell proliferation | 103 | 2.22E-19 | 2.46 | 4.11E-16 |
| GO:0051960 | regulation of nervous system development | 68 | 3.21E-19 | 3.12 | 5.95E-16 |
| GO:0048729 | tissue morphogenesis | 91 | 6.36E-19 | 2.59 | 1.18E-15 |
| GO:0010604 | positive regulation of macromolecule metabolic process | 177 | 1.41E-18 | 1.90 | 2.62E-15 |
| GO:0009792 | embryonic development ending in birth or egg hatching | 133 | 2.03E-18 | 2.12 | 3.76E-15 |
| GO:0043009 | chordate embryonic development | 132 | 2.31E-18 | 2.13 | 4.28E-15 |
| GO:0001763 | morphogenesis of a branching structure | 60 | 3.65E-18 | 3.25 | 6.77E-15 |

**Table S17. Biological functions significantly (FDR<0.05) implicated in genes that lost or showed decreased H3K4me3 marks from 1- to 4-wk old kidney by DAVID gene ontology analysis**

| **GO number** | **GO Term** | **Count** | **P-Value** | **Fold Enrichment** | **FDR** |
| --- | --- | --- | --- | --- | --- |
| GO:0051301 | cell division | 76 | 4.31E-11 | 2.18 | 7.87E-08 |
| GO:0034660 | ncRNA metabolic process | 58 | 9.92E-10 | 2.32 | 1.81E-06 |
| GO:0046907 | intracellular transport | 98 | 1.97E-09 | 1.83 | 3.61E-06 |
| GO:0006396 | RNA processing | 97 | 8.87E-09 | 1.79 | 1.62E-05 |
| GO:0045184 | establishment of protein localization | 132 | 1.04E-08 | 1.62 | 1.90E-05 |
| GO:0015031 | protein transport | 130 | 2.22E-08 | 1.61 | 4.06E-05 |
| GO:0045449 | regulation of transcription | 354 | 7.42E-08 | 1.28 | 1.36E-04 |
| GO:0006350 | transcription | 291 | 8.21E-08 | 1.33 | 1.50E-04 |
| GO:0000280 | nuclear division | 51 | 1.22E-07 | 2.17 | 2.22E-04 |
| GO:0007067 | mitosis | 51 | 1.22E-07 | 2.17 | 2.22E-04 |
| GO:0007507 | heart development | 57 | 1.23E-07 | 2.06 | 2.25E-04 |
| GO:0048285 | organelle fission | 52 | 1.60E-07 | 2.13 | 2.93E-04 |
| GO:0000087 | M phase of mitotic cell cycle | 51 | 2.45E-07 | 2.12 | 4.48E-04 |
| GO:0008104 | protein localization | 141 | 3.13E-07 | 1.51 | 5.71E-04 |
| GO:0000278 | mitotic cell cycle | 59 | 5.44E-07 | 1.95 | 9.94E-04 |
| GO:0007049 | cell cycle | 118 | 6.59E-07 | 1.56 | 0.0012042 |
| GO:0034470 | ncRNA processing | 43 | 9.14E-07 | 2.20 | 0.0016688 |
| GO:0006399 | tRNA metabolic process | 34 | 1.40E-06 | 2.43 | 0.0025597 |
| GO:0006793 | phosphorus metabolic process | 153 | 3.31E-06 | 1.43 | 0.006048 |
| GO:0006796 | phosphate metabolic process | 153 | 3.31E-06 | 1.43 | 0.006048 |
| GO:0035295 | tube development | 60 | 3.71E-06 | 1.83 | 0.0067667 |
| GO:0001568 | blood vessel development | 56 | 5.98E-06 | 1.85 | 0.0109238 |
| GO:0001944 | vasculature development | 57 | 6.04E-06 | 1.84 | 0.0110197 |
| GO:0022613 | ribonucleoprotein complex biogenesis | 37 | 7.14E-06 | 2.18 | 0.0130352 |
| GO:0022402 | cell cycle process | 80 | 7.19E-06 | 1.64 | 0.0131286 |
| GO:0030029 | actin filament-based process | 44 | 7.42E-06 | 2.02 | 0.0135412 |
| GO:0000279 | M phase | 62 | 8.64E-06 | 1.77 | 0.0157846 |
| GO:0042254 | ribosome biogenesis | 32 | 9.79E-06 | 2.31 | 0.0178831 |
| GO:0022403 | cell cycle phase | 69 | 1.08E-05 | 1.70 | 0.0196989 |
| GO:0006915 | apoptosis | 90 | 1.49E-05 | 1.56 | 0.0271506 |
| GO:0006259 | DNA metabolic process | 83 | 1.66E-05 | 1.59 | 0.0302909 |
| GO:0006418 | tRNA aminoacylation for protein translation | 18 | 1.77E-05 | 3.16 | 0.0323776 |
| GO:0043039 | tRNA aminoacylation | 18 | 1.77E-05 | 3.16 | 0.0323776 |
| GO:0043038 | amino acid activation | 18 | 1.77E-05 | 3.16 | 0.0323776 |
| GO:0030031 | cell projection assembly | 23 | 2.21E-05 | 2.65 | 0.0403044 |
| GO:0016310 | phosphorylation | 127 | 2.36E-05 | 1.43 | 0.0430137 |

**Table S18. Biological functions significantly (FDR<0.05) implicated in genes that lost or showed decreased H3K4me3 marks from 1- to 4-wk old lung by DAVID gene ontology analysis**

| **GO number** | **GO Term** | **Count** | **P-Value** | **Fold Enrichment** | **FDR** |
| --- | --- | --- | --- | --- | --- |
| GO:0007049 | cell cycle | 166 | 8.10E-19 | 1.96 | 1.49E-15 |
| GO:0006396 | RNA processing | 122 | 9.66E-15 | 2.01 | 1.77E-11 |
| GO:0051301 | cell division | 88 | 6.98E-14 | 2.26 | 1.28E-10 |
| GO:0000278 | mitotic cell cycle | 77 | 1.94E-12 | 2.28 | 3.56E-09 |
| GO:0000087 | M phase of mitotic cell cycle | 64 | 2.11E-11 | 2.38 | 3.87E-08 |
| GO:0006259 | DNA metabolic process | 110 | 2.19E-11 | 1.89 | 4.02E-08 |
| GO:0022402 | cell cycle process | 104 | 3.67E-11 | 1.91 | 6.74E-08 |
| GO:0048285 | organelle fission | 63 | 1.32E-10 | 2.31 | 2.42E-07 |
| GO:0000280 | nuclear division | 61 | 2.25E-10 | 2.32 | 4.13E-07 |
| GO:0007067 | mitosis | 61 | 2.25E-10 | 2.32 | 4.13E-07 |
| GO:0022403 | cell cycle phase | 88 | 6.51E-10 | 1.94 | 1.20E-06 |
| GO:0046907 | intracellular transport | 107 | 1.02E-09 | 1.79 | 1.88E-06 |
| GO:0006281 | DNA repair | 66 | 1.38E-09 | 2.15 | 2.54E-06 |
| GO:0000279 | M phase | 78 | 1.82E-09 | 1.99 | 3.35E-06 |
| GO:0034660 | ncRNA metabolic process | 61 | 3.24E-09 | 2.18 | 5.94E-06 |
| GO:0006974 | response to DNA damage stimulus | 77 | 8.53E-09 | 1.94 | 1.57E-05 |
| GO:0034470 | ncRNA processing | 50 | 1.92E-08 | 2.28 | 3.53E-05 |
| GO:0006412 | translation | 81 | 4.75E-08 | 1.83 | 8.72E-05 |
| GO:0008104 | protein localization | 157 | 6.75E-08 | 1.50 | 1.24E-04 |
| GO:0033554 | cellular response to stress | 95 | 1.57E-07 | 1.70 | 2.88E-04 |
| GO:0045184 | establishment of protein localization | 139 | 1.60E-07 | 1.53 | 2.93E-04 |
| GO:0051726 | regulation of cell cycle | 59 | 2.09E-07 | 1.99 | 3.84E-04 |
| GO:0015031 | protein transport | 137 | 3.01E-07 | 1.52 | 5.52E-04 |
| GO:0044265 | cellular macromolecule catabolic process | 129 | 4.77E-07 | 1.53 | 8.75E-04 |
| GO:0009057 | macromolecule catabolic process | 136 | 6.71E-07 | 1.50 | 0.0012319 |
| GO:0006399 | tRNA metabolic process | 37 | 6.94E-07 | 2.36 | 0.0012742 |
| GO:0006350 | transcription | 314 | 7.86E-07 | 1.28 | 0.0014434 |
| GO:0016071 | mRNA metabolic process | 74 | 8.39E-07 | 1.77 | 0.0015416 |
| GO:0051603 | proteolysis involved in cellular protein catabolic process | 114 | 1.63E-06 | 1.54 | 0.002987 |
| GO:0044257 | cellular protein catabolic process | 114 | 2.15E-06 | 1.53 | 0.0039541 |
| GO:0019941 | modification-dependent protein catabolic process | 109 | 2.19E-06 | 1.55 | 0.0040175 |
| GO:0043632 | modification-dependent macromolecule catabolic process | 109 | 2.19E-06 | 1.55 | 0.0040175 |
| GO:0030163 | protein catabolic process | 117 | 2.44E-06 | 1.52 | 0.0044792 |
| GO:0006793 | phosphorus metabolic process | 167 | 4.50E-06 | 1.39 | 0.0082642 |
| GO:0006796 | phosphate metabolic process | 167 | 4.50E-06 | 1.39 | 0.0082642 |
| GO:0006397 | mRNA processing | 64 | 5.59E-06 | 1.76 | 0.0102723 |
| GO:0070727 | cellular macromolecule localization | 71 | 6.04E-06 | 1.70 | 0.0110865 |
| GO:0002009 | morphogenesis of an epithelium | 47 | 6.42E-06 | 1.96 | 0.0117921 |
| GO:0006913 | nucleocytoplasmic transport | 31 | 8.87E-06 | 2.33 | 0.016279 |
| GO:0034613 | cellular protein localization | 70 | 9.27E-06 | 1.69 | 0.0170189 |
| GO:0045449 | regulation of transcription | 374 | 1.22E-05 | 1.21 | 0.0224044 |
| GO:0051169 | nuclear transport | 31 | 1.40E-05 | 2.28 | 0.0256576 |
| GO:0006886 | intracellular protein transport | 65 | 1.64E-05 | 1.70 | 0.0301864 |
| GO:0043933 | macromolecular complex subunit organization | 81 | 1.79E-05 | 1.59 | 0.0327954 |
| GO:0007059 | chromosome segregation | 23 | 2.64E-05 | 2.59 | 0.0485127 |

**Table S19. Biological functions significantly (FDR<0.05) implicated in genes that have acquired or showed increased H3K4me3 marks from 1- to 4-wk old kidney by DAVID gene ontology analysis**

| **GO number** | **GO Term** | **Count** | **P-Value** | **Fold Enrichment** | **FDR** |
| --- | --- | --- | --- | --- | --- |
| GO:0055114 | oxidation reduction | 157 | 1.26E-11 | 1.69 | 2.32E-08 |
| GO:0008104 | protein localization | 164 | 1.29E-09 | 1.57 | 2.36E-06 |
| GO:0006091 | generation of precursor metabolites and energy | 73 | 3.15E-09 | 2.02 | 5.77E-06 |
| GO:0009057 | macromolecule catabolic process | 145 | 3.84E-09 | 1.60 | 7.03E-06 |
| GO:0030163 | protein catabolic process | 126 | 1.19E-08 | 1.64 | 2.18E-05 |
| GO:0045184 | establishment of protein localization | 143 | 1.57E-08 | 1.57 | 2.88E-05 |
| GO:0015031 | protein transport | 142 | 1.70E-08 | 1.57 | 3.12E-05 |
| GO:0044265 | cellular macromolecule catabolic process | 134 | 2.62E-08 | 1.59 | 4.80E-05 |
| GO:0019941 | modification-dependent protein catabolic process | 115 | 5.98E-08 | 1.63 | 1.10E-04 |
| GO:0043632 | modification-dependent macromolecule catabolic process | 115 | 5.98E-08 | 1.63 | 1.10E-04 |
| GO:0051603 | proteolysis involved in cellular protein catabolic process | 119 | 8.43E-08 | 1.61 | 1.54E-04 |
| GO:0044257 | cellular protein catabolic process | 119 | 1.16E-07 | 1.60 | 2.13E-04 |
| GO:0022900 | electron transport chain | 38 | 1.72E-07 | 2.45 | 3.15E-04 |
| GO:0046907 | intracellular transport | 99 | 2.67E-07 | 1.66 | 4.89E-04 |
| GO:0051186 | cofactor metabolic process | 52 | 3.62E-07 | 2.06 | 6.63E-04 |
| GO:0070727 | cellular macromolecule localization | 74 | 7.19E-07 | 1.78 | 0.0013177 |
| GO:0006732 | coenzyme metabolic process | 43 | 9.89E-07 | 2.17 | 0.0018118 |
| GO:0034613 | cellular protein localization | 73 | 1.14E-06 | 1.76 | 0.0020829 |
| GO:0042981 | regulation of apoptosis | 117 | 1.79E-06 | 1.53 | 0.0032817 |
| GO:0043067 | regulation of programmed cell death | 117 | 3.41E-06 | 1.51 | 0.006245 |
| GO:0010941 | regulation of cell death | 117 | 4.42E-06 | 1.50 | 0.0081075 |
| GO:0043068 | positive regulation of programmed cell death | 62 | 4.69E-06 | 1.79 | 0.0085952 |
| GO:0055085 | transmembrane transport | 99 | 5.68E-06 | 1.55 | 0.010403 |
| GO:0010942 | positive regulation of cell death | 62 | 6.15E-06 | 1.78 | 0.011277 |
| GO:0008610 | lipid biosynthetic process | 68 | 6.35E-06 | 1.72 | 0.0116421 |
| GO:0043065 | positive regulation of apoptosis | 61 | 7.44E-06 | 1.78 | 0.0136367 |
| GO:0006886 | intracellular protein transport | 66 | 8.17E-06 | 1.73 | 0.0149698 |
| GO:0016126 | sterol biosynthetic process | 15 | 1.49E-05 | 3.61 | 0.0272251 |

**Table S20. Biological functions significantly (FDR<0.05) implicated in genes that have acquired or showed increased H3K4me3 marks from 1- to 4-wk old lung by DAVID gene ontology analysis**

| **GO number** | **GO Term** | **Count** | **P-Value** | **Fold Enrichment** | **FDR** |
| --- | --- | --- | --- | --- | --- |
| GO:0008104 | protein localization | 153 | 1.77E-14 | 1.85 | 3.23E-11 |
| GO:0045184 | establishment of protein localization | 133 | 1.40E-12 | 1.85 | 2.56E-09 |
| GO:0015031 | protein transport | 131 | 3.63E-12 | 1.83 | 6.64E-09 |
| GO:0006350 | transcription | 271 | 1.96E-09 | 1.39 | 3.58E-06 |
| GO:0046907 | intracellular transport | 89 | 4.28E-09 | 1.88 | 7.82E-06 |
| GO:0045449 | regulation of transcription | 324 | 9.03E-09 | 1.33 | 1.65E-05 |
| GO:0006396 | RNA processing | 82 | 1.26E-06 | 1.71 | 0.002304 |
| GO:0070727 | cellular macromolecule localization | 62 | 1.34E-06 | 1.88 | 0.0024574 |
| GO:0006886 | intracellular protein transport | 58 | 1.59E-06 | 1.92 | 0.0029107 |
| GO:0034613 | cellular protein localization | 61 | 2.29E-06 | 1.86 | 0.0041919 |
| GO:0007005 | mitochondrion organization | 28 | 3.39E-06 | 2.63 | 0.0062047 |
| GO:0016071 | mRNA metabolic process | 59 | 1.37E-05 | 1.78 | 0.0249585 |
| GO:0017038 | protein import | 24 | 1.54E-05 | 2.67 | 0.0282032 |
| GO:0016568 | chromatin modification | 49 | 1.59E-05 | 1.89 | 0.0290834 |
| GO:0045637 | regulation of myeloid cell differentiation | 18 | 1.69E-05 | 3.22 | 0.030807 |
| GO:0006325 | chromatin organization | 60 | 2.51E-05 | 1.74 | 0.0458924 |

**Table S21. Biological functions significantly (FDR<0.05) implicated in genes that lost or showed decreased H3K27me3 marks from 1- to 4-wk old kidney by DAVID gene ontology analysis**

| **GO number** | **GO Term** | **Count** | **P-Value** | **Fold Enrichment** | **FDR** |
| --- | --- | --- | --- | --- | --- |
| GO:0048598 | embryonic morphogenesis | 69 | 1.28E-19 | 3.45 | 2.28E-16 |
| GO:0051252 | regulation of RNA metabolic process | 161 | 1.63E-17 | 1.94 | 2.90E-14 |
| GO:0007389 | pattern specification process | 57 | 3.63E-17 | 3.61 | 6.45E-14 |
| GO:0006355 | regulation of transcription, DNA-dependent | 158 | 4.83E-17 | 1.94 | 8.57E-14 |
| GO:0030182 | neuron differentiation | 68 | 2.64E-16 | 3.06 | 4.00E-13 |
| GO:0048568 | embryonic organ development | 51 | 3.25E-16 | 3.80 | 5.88E-13 |
| GO:0035295 | tube development | 53 | 6.34E-16 | 3.61 | 1.19E-12 |
| GO:0003002 | regionalization | 47 | 1.07E-15 | 3.95 | 1.98E-12 |
| GO:0045941 | positive regulation of transcription | 73 | 3.99E-15 | 2.76 | 7.09E-12 |
| GO:0010628 | positive regulation of gene expression | 73 | 1.65E-14 | 2.69 | 2.94E-11 |
| GO:0042127 | regulation of cell proliferation | 77 | 3.06E-14 | 2.57 | 5.42E-11 |
| GO:0045935 | positive regulation of nucleobase, nucleoside, nucleotide and nucleic acid metabolic process | 74 | 5.16E-14 | 2.61 | 9.16E-11 |
| GO:0008284 | positive regulation of cell proliferation | 52 | 6.30E-14 | 3.29 | 1.12E-10 |
| GO:0045893 | positive regulation of transcription, DNA-dependent | 65 | 7.31E-14 | 2.81 | 1.30E-10 |
| GO:0007423 | sensory organ development | 49 | 7.62E-14 | 3.43 | 1.35E-10 |
| GO:0048562 | embryonic organ morphogenesis | 38 | 7.85E-14 | 4.24 | 1.39E-10 |
| GO:0006357 | regulation of transcription from RNA polymerase II promoter | 83 | 7.95E-14 | 2.42 | 1.41E-10 |
| GO:0051173 | positive regulation of nitrogen compound metabolic process | 75 | 8.60E-14 | 2.56 | 1.53E-10 |
| GO:0051254 | positive regulation of RNA metabolic process | 65 | 1.03E-13 | 2.79 | 1.82E-10 |
| GO:0060429 | epithelium development | 50 | 1.52E-13 | 3.32 | 2.69E-10 |
| GO:0009891 | positive regulation of biosynthetic process | 77 | 1.94E-13 | 2.48 | 3.45E-10 |
| GO:0048666 | neuron development | 52 | 1.96E-13 | 3.20 | 3.47E-10 |
| GO:0009792 | embryonic development ending in birth or egg hatching | 65 | 2.01E-13 | 2.75 | 3.56E-10 |
| GO:0031328 | positive regulation of cellular biosynthetic process | 76 | 3.55E-13 | 2.47 | 6.31E-10 |
| GO:0010557 | positive regulation of macromolecule biosynthetic process | 74 | 3.78E-13 | 2.51 | 6.70E-10 |
| GO:0043009 | chordate embryonic development | 64 | 4.18E-13 | 2.73 | 7.42E-10 |
| GO:0045944 | positive regulation of transcription from RNA polymerase II promoter | 58 | 4.20E-13 | 2.91 | 7.46E-10 |
| GO:0035239 | tube morphogenesis | 38 | 5.70E-13 | 3.99 | 1.01E-09 |
| GO:0045449 | regulation of transcription | 199 | 1.03E-12 | 1.61 | 1.83E-09 |
| GO:0009952 | anterior/posterior pattern formation | 35 | 2.26E-12 | 4.11 | 4.01E-09 |
| GO:0048732 | gland development | 39 | 1.15E-11 | 3.56 | 2.05E-08 |
| GO:0045165 | cell fate commitment | 33 | 1.80E-11 | 4.03 | 3.20E-08 |
| GO:0048729 | tissue morphogenesis | 43 | 1.96E-11 | 3.25 | 3.48E-08 |
| GO:0048667 | cell morphogenesis involved in neuron differentiation | 36 | 8.26E-11 | 3.56 | 1.47E-07 |
| GO:0002009 | morphogenesis of an epithelium | 35 | 8.32E-11 | 3.64 | 1.48E-07 |
| GO:0007267 | cell-cell signaling | 47 | 9.44E-11 | 2.91 | 1.68E-07 |
| GO:0000904 | cell morphogenesis involved in differentiation | 39 | 1.13E-10 | 3.31 | 2.00E-07 |
| GO:0010604 | positive regulation of macromolecule metabolic process | 77 | 1.17E-10 | 2.19 | 2.08E-07 |
| GO:0048812 | neuron projection morphogenesis | 35 | 1.36E-10 | 3.57 | 2.41E-07 |
| GO:0030900 | forebrain development | 34 | 1.36E-10 | 3.66 | 2.41E-07 |
| GO:0007409 | axonogenesis | 33 | 3.09E-10 | 3.64 | 5.48E-07 |
| GO:0051960 | regulation of nervous system development | 31 | 4.94E-10 | 3.76 | 8.76E-07 |
| GO:0001501 | skeletal system development | 45 | 5.93E-10 | 2.84 | 1.05E-06 |
| GO:0006350 | transcription | 158 | 6.80E-10 | 1.60 | 1.21E-06 |
| GO:0031175 | neuron projection development | 38 | 9.81E-10 | 3.13 | 1.74E-06 |
| GO:0048858 | cell projection morphogenesis | 36 | 1.59E-09 | 3.20 | 2.81E-06 |
| GO:0043583 | ear development | 25 | 1.76E-09 | 4.32 | 3.12E-06 |
| GO:0048663 | neuron fate commitment | 17 | 2.09E-09 | 6.50 | 3.72E-06 |
| GO:0042472 | inner ear morphogenesis | 20 | 2.19E-09 | 5.37 | 3.88E-06 |
| GO:0042471 | ear morphogenesis | 21 | 3.49E-09 | 4.97 | 6.20E-06 |
| GO:0032990 | cell part morphogenesis | 36 | 5.91E-09 | 3.05 | 1.05E-05 |
| GO:0030030 | cell projection organization | 46 | 7.04E-09 | 2.59 | 1.25E-05 |
| GO:0000902 | cell morphogenesis | 45 | 7.71E-09 | 2.62 | 1.37E-05 |
| GO:0048839 | inner ear development | 22 | 9.63E-09 | 4.49 | 1.71E-05 |
| GO:0060284 | regulation of cell development | 30 | 1.22E-08 | 3.39 | 2.17E-05 |
| GO:0050767 | regulation of neurogenesis | 27 | 1.33E-08 | 3.68 | 2.37E-05 |
| GO:0007155 | cell adhesion | 64 | 6.50E-08 | 2.05 | 1.15E-04 |
| GO:0022610 | biological adhesion | 64 | 6.95E-08 | 2.05 | 1.23E-04 |
| GO:0001568 | blood vessel development | 37 | 7.28E-08 | 2.73 | 1.29E-04 |
| GO:0032989 | cellular component morphogenesis | 46 | 1.29E-07 | 2.36 | 2.29E-04 |
| GO:0001944 | vasculature development | 37 | 1.36E-07 | 2.66 | 2.41E-04 |
| GO:0045664 | regulation of neuron differentiation | 22 | 1.50E-07 | 3.88 | 2.66E-04 |
| GO:0045596 | negative regulation of cell differentiation | 30 | 2.61E-07 | 2.96 | 4.63E-04 |
| GO:0035113 | embryonic appendage morphogenesis | 21 | 2.89E-07 | 3.89 | 5.12E-04 |
| GO:0030326 | embryonic limb morphogenesis | 21 | 2.89E-07 | 3.89 | 5.12E-04 |
| GO:0001763 | morphogenesis of a branching structure | 24 | 3.34E-07 | 3.45 | 5.92E-04 |
| GO:0007167 | enzyme linked receptor protein signaling pathway | 38 | 4.32E-07 | 2.50 | 7.67E-04 |
| GO:0048514 | blood vessel morphogenesis | 31 | 4.99E-07 | 2.81 | 8.85E-04 |
| GO:0048706 | embryonic skeletal system development | 19 | 5.12E-07 | 4.11 | 9.10E-04 |
| GO:0000122 | negative regulation of transcription from RNA polymerase II promoter | 34 | 5.43E-07 | 2.65 | 9.63E-04 |
| GO:0021515 | cell differentiation in spinal cord | 12 | 6.38E-07 | 6.74 | 0.0011327 |
| GO:0060562 | epithelial tube morphogenesis | 22 | 6.68E-07 | 3.56 | 0.0011853 |
| GO:0051094 | positive regulation of developmental process | 32 | 8.80E-07 | 2.69 | 0.0015621 |
| GO:0030879 | mammary gland development | 19 | 8.94E-07 | 3.97 | 0.0015871 |
| GO:0035108 | limb morphogenesis | 22 | 1.23E-06 | 3.44 | 0.0021766 |
| GO:0035107 | appendage morphogenesis | 22 | 1.23E-06 | 3.44 | 0.0021766 |
| GO:0007411 | axon guidance | 20 | 1.54E-06 | 3.67 | 0.0027251 |
| GO:0048736 | appendage development | 22 | 2.18E-06 | 3.32 | 0.0038762 |
| GO:0060173 | limb development | 22 | 2.18E-06 | 3.32 | 0.0038762 |
| GO:0048754 | branching morphogenesis of a tube | 19 | 2.96E-06 | 3.67 | 0.0052496 |
| GO:0048638 | regulation of developmental growth | 12 | 3.27E-06 | 5.83 | 0.0058126 |
| GO:0010648 | negative regulation of cell communication | 28 | 4.26E-06 | 2.71 | 0.0075545 |
| GO:0060541 | respiratory system development | 22 | 4.32E-06 | 3.19 | 0.0076631 |
| GO:0021510 | spinal cord development | 13 | 5.37E-06 | 5.08 | 0.0095243 |
| GO:0006928 | cell motion | 43 | 6.70E-06 | 2.11 | 0.0118849 |
| GO:0016337 | cell-cell adhesion | 32 | 7.11E-06 | 2.44 | 0.0126201 |
| GO:0035270 | endocrine system development | 16 | 7.67E-06 | 3.99 | 0.0136121 |
| GO:0009968 | negative regulation of signal transduction | 26 | 8.43E-06 | 2.73 | 0.0149688 |
| GO:0048592 | eye morphogenesis | 16 | 9.16E-06 | 3.94 | 0.0162656 |
| GO:0045665 | negative regulation of neuron differentiation | 11 | 1.02E-05 | 5.81 | 0.018174 |
| GO:0030324 | lung development | 20 | 1.04E-05 | 3.24 | 0.0185367 |
| GO:0030323 | respiratory tube development | 20 | 1.36E-05 | 3.18 | 0.0241665 |
| GO:0002053 | positive regulation of mesenchymal cell proliferation | 9 | 1.39E-05 | 7.35 | 0.0247474 |
| GO:0007507 | heart development | 30 | 1.69E-05 | 2.42 | 0.0300462 |
| GO:0007268 | synaptic transmission | 26 | 1.71E-05 | 2.63 | 0.0304342 |
| GO:0001654 | eye development | 24 | 1.85E-05 | 2.75 | 0.03292 |
| GO:0045892 | negative regulation of transcription, DNA-dependent | 37 | 1.92E-05 | 2.16 | 0.0340212 |
| GO:0001503 | ossification | 19 | 1.99E-05 | 3.22 | 0.035248 |
| GO:0010464 | regulation of mesenchymal cell proliferation | 9 | 2.03E-05 | 7.03 | 0.0361046 |
| GO:0051253 | negative regulation of RNA metabolic process | 37 | 2.21E-05 | 2.15 | 0.039204 |
| GO:0007169 | transmembrane receptor protein tyrosine kinase signaling pathway | 27 | 2.25E-05 | 2.53 | 0.0399441 |

**Table S22. Biological functions significantly (FDR<0.05) implicated in genes that lost or showed decreased H3K27me3 marks from 1- to 4-wk old lung by DAVID gene ontology analysis**

| **GO number** | **GO Term** | **Count** | **P-Value** | **Fold Enrichment** | **FDR** |
| --- | --- | --- | --- | --- | --- |
| GO:0030182 | neuron differentiation | 64 | 7.13E-19 | 3.58 | 1.25E-15 |
| GO:0048598 | embryonic morphogenesis | 53 | 4.40E-14 | 3.29 | 7.71E-11 |
| GO:0007389 | pattern specification process | 46 | 9.45E-14 | 3.61 | 1.66E-10 |
| GO:0048666 | neuron development | 45 | 1.08E-12 | 3.44 | 1.90E-09 |
| GO:0048568 | embryonic organ development | 40 | 2.26E-12 | 3.70 | 3.96E-09 |
| GO:0007267 | cell-cell signaling | 43 | 1.35E-11 | 3.31 | 2.37E-08 |
| GO:0045944 | positive regulation of transcription from RNA polymerase II promoter | 48 | 2.70E-11 | 2.99 | 4.73E-08 |
| GO:0045893 | positive regulation of transcription, DNA-dependent | 52 | 4.93E-11 | 2.79 | 8.64E-08 |
| GO:0051254 | positive regulation of RNA metabolic process | 52 | 6.36E-11 | 2.77 | 1.12E-07 |
| GO:0031175 | neuron projection development | 35 | 1.68E-10 | 3.58 | 2.95E-07 |
| GO:0006355 | regulation of transcription, DNA-dependent | 118 | 1.83E-10 | 1.80 | 3.21E-07 |
| GO:0006357 | regulation of transcription from RNA polymerase II promoter | 65 | 2.09E-10 | 2.35 | 3.66E-07 |
| GO:0051252 | regulation of RNA metabolic process | 119 | 2.31E-10 | 1.78 | 4.04E-07 |
| GO:0003002 | regionalization | 34 | 4.25E-10 | 3.54 | 7.46E-07 |
| GO:0048562 | embryonic organ morphogenesis | 29 | 5.55E-10 | 4.02 | 9.74E-07 |
| GO:0045941 | positive regulation of transcription | 54 | 6.70E-10 | 2.54 | 1.18E-06 |
| GO:0007423 | sensory organ development | 37 | 1.04E-09 | 3.21 | 1.82E-06 |
| GO:0000904 | cell morphogenesis involved in differentiation | 33 | 1.36E-09 | 3.47 | 2.39E-06 |
| GO:0010628 | positive regulation of gene expression | 54 | 1.76E-09 | 2.47 | 3.09E-06 |
| GO:0006928 | cell motion | 45 | 2.23E-09 | 2.74 | 3.90E-06 |
| GO:0010557 | positive regulation of macromolecule biosynthetic process | 56 | 4.46E-09 | 2.36 | 7.82E-06 |
| GO:0048812 | neuron projection morphogenesis | 29 | 4.47E-09 | 3.68 | 7.84E-06 |
| GO:0048858 | cell projection morphogenesis | 31 | 6.62E-09 | 3.42 | 1.16E-05 |
| GO:0045165 | cell fate commitment | 26 | 7.54E-09 | 3.95 | 1.32E-05 |
| GO:0042127 | regulation of cell proliferation | 56 | 7.68E-09 | 2.32 | 1.35E-05 |
| GO:0045935 | positive regulation of nucleobase, nucleoside, nucleotide and nucleic acid metabolic process | 54 | 8.23E-09 | 2.36 | 1.44E-05 |
| GO:0051173 | positive regulation of nitrogen compound metabolic process | 55 | 9.05E-09 | 2.33 | 1.59E-05 |
| GO:0048667 | cell morphogenesis involved in neuron differentiation | 29 | 9.60E-09 | 3.56 | 1.68E-05 |
| GO:0009891 | positive regulation of biosynthetic process | 57 | 1.00E-08 | 2.28 | 1.75E-05 |
| GO:0007409 | axonogenesis | 27 | 1.51E-08 | 3.70 | 2.65E-05 |
| GO:0031328 | positive regulation of cellular biosynthetic process | 56 | 1.87E-08 | 2.26 | 3.27E-05 |
| GO:0032990 | cell part morphogenesis | 31 | 2.07E-08 | 3.26 | 3.63E-05 |
| GO:0030900 | forebrain development | 27 | 2.53E-08 | 3.61 | 4.43E-05 |
| GO:0035295 | tube development | 35 | 2.65E-08 | 2.96 | 4.64E-05 |
| GO:0030030 | cell projection organization | 39 | 3.39E-08 | 2.73 | 5.94E-05 |
| GO:0000902 | cell morphogenesis | 38 | 4.49E-08 | 2.74 | 7.88E-05 |
| GO:0010604 | positive regulation of macromolecule metabolic process | 60 | 6.18E-08 | 2.11 | 1.08E-04 |
| GO:0032989 | cellular component morphogenesis | 40 | 1.46E-07 | 2.54 | 2.57E-04 |
| GO:0045449 | regulation of transcription | 148 | 2.80E-07 | 1.48 | 4.92E-04 |
| GO:0048705 | skeletal system morphogenesis | 22 | 2.95E-07 | 3.78 | 5.18E-04 |
| GO:0006350 | transcription | 122 | 7.95E-07 | 1.54 | 0.0013937 |
| GO:0009952 | anterior/posterior pattern formation | 23 | 1.21E-06 | 3.35 | 0.0021275 |
| GO:0007411 | axon guidance | 18 | 1.43E-06 | 4.10 | 0.0025139 |
| GO:0048839 | inner ear development | 17 | 1.53E-06 | 4.31 | 0.0026771 |
| GO:0035239 | tube morphogenesis | 24 | 2.28E-06 | 3.13 | 0.0039892 |
| GO:0007155 | cell adhesion | 51 | 2.45E-06 | 2.03 | 0.0042983 |
| GO:0022610 | biological adhesion | 51 | 2.57E-06 | 2.02 | 0.0045064 |
| GO:0010648 | negative regulation of cell communication | 25 | 2.90E-06 | 3.00 | 0.0050872 |
| GO:0043583 | ear development | 18 | 3.36E-06 | 3.86 | 0.0058827 |
| GO:0008284 | positive regulation of cell proliferation | 32 | 4.09E-06 | 2.51 | 0.0071641 |
| GO:0001501 | skeletal system development | 32 | 4.39E-06 | 2.51 | 0.0076963 |
| GO:0009968 | negative regulation of signal transduction | 23 | 7.84E-06 | 3.00 | 0.0137486 |
| GO:0051216 | cartilage development | 15 | 8.02E-06 | 4.29 | 0.0140656 |
| GO:0048729 | tissue morphogenesis | 28 | 8.37E-06 | 2.62 | 0.0146657 |
| GO:0006813 | potassium ion transport | 22 | 9.19E-06 | 3.07 | 0.016113 |
| GO:0019226 | transmission of nerve impulse | 27 | 9.52E-06 | 2.67 | 0.0166936 |
| GO:0006811 | ion transport | 58 | 1.32E-05 | 1.82 | 0.023089 |
| GO:0001568 | blood vessel development | 28 | 1.32E-05 | 2.56 | 0.0231953 |
| GO:0043009 | chordate embryonic development | 40 | 1.33E-05 | 2.12 | 0.0232714 |
| GO:0007167 | enzyme linked receptor protein signaling pathway | 30 | 1.41E-05 | 2.45 | 0.0247314 |
| GO:0009792 | embryonic development ending in birth or egg hatching | 40 | 1.65E-05 | 2.10 | 0.0289718 |
| GO:0030001 | metal ion transport | 41 | 1.78E-05 | 2.07 | 0.0312777 |
| GO:0001944 | vasculature development | 28 | 2.06E-05 | 2.50 | 0.0360266 |
| GO:0001525 | angiogenesis | 19 | 2.61E-05 | 3.19 | 0.0456735 |
| GO:0048514 | blood vessel morphogenesis | 24 | 2.66E-05 | 2.70 | 0.0465985 |
| GO:0016477 | cell migration | 27 | 2.76E-05 | 2.51 | 0.0484139 |

**Table S23. Biological functions significantly (FDR<0.05) implicated in genes that have acquired or showed increased H3K27me3 marks from 1- to 4-wk old kidney by DAVID gene ontology analysis**

| **GO number** | **GO Term** | **Count** | **P-Value** | **Fold Enrichment** | **FDR** |
| --- | --- | --- | --- | --- | --- |
| GO:0030001 | metal ion transport | 70 | 4.53E-13 | 2.58 | 8.14E-10 |
| GO:0044057 | regulation of system process | 42 | 6.36E-12 | 3.40 | 1.14E-08 |
| GO:0007267 | cell-cell signaling | 51 | 2.25E-11 | 2.86 | 4.04E-08 |
| GO:0030799 | regulation of cyclic nucleotide metabolic process | 24 | 2.98E-11 | 5.35 | 5.35E-08 |
| GO:0006140 | regulation of nucleotide metabolic process | 24 | 5.55E-11 | 5.21 | 9.96E-08 |
| GO:0007610 | behavior | 61 | 1.46E-10 | 2.45 | 2.62E-07 |
| GO:0006811 | ion transport | 89 | 1.51E-10 | 2.03 | 2.72E-07 |
| GO:0006812 | cation transport | 70 | 5.48E-10 | 2.21 | 9.85E-07 |
| GO:0030802 | regulation of cyclic nucleotide biosynthetic process | 22 | 6.01E-10 | 5.11 | 1.08E-06 |
| GO:0030808 | regulation of nucleotide biosynthetic process | 22 | 6.01E-10 | 5.11 | 1.08E-06 |
| GO:0048562 | embryonic organ morphogenesis | 33 | 2.65E-09 | 3.34 | 4.75E-06 |
| GO:0006928 | cell motion | 54 | 4.46E-09 | 2.39 | 8.01E-06 |
| GO:0030182 | neuron differentiation | 57 | 4.65E-09 | 2.32 | 8.35E-06 |
| GO:0048568 | embryonic organ development | 41 | 7.22E-09 | 2.77 | 1.30E-05 |
| GO:0030814 | regulation of cAMP metabolic process | 20 | 1.12E-08 | 4.86 | 2.01E-05 |
| GO:0019226 | transmission of nerve impulse | 39 | 1.20E-08 | 2.81 | 2.15E-05 |
| GO:0007626 | locomotory behavior | 40 | 1.81E-08 | 2.72 | 3.25E-05 |
| GO:0007155 | cell adhesion | 70 | 2.02E-08 | 2.03 | 3.63E-05 |
| GO:0022610 | biological adhesion | 70 | 2.15E-08 | 2.03 | 3.86E-05 |
| GO:0007268 | synaptic transmission | 33 | 3.37E-08 | 3.02 | 6.06E-05 |
| GO:0030817 | regulation of cAMP biosynthetic process | 19 | 4.09E-08 | 4.76 | 7.35E-05 |
| GO:0006813 | potassium ion transport | 30 | 1.23E-07 | 3.05 | 2.21E-04 |
| GO:0006816 | calcium ion transport | 25 | 2.67E-07 | 3.36 | 4.80E-04 |
| GO:0048598 | embryonic morphogenesis | 49 | 2.68E-07 | 2.22 | 4.82E-04 |
| GO:0015674 | di-, tri-valent inorganic cation transport | 29 | 4.91E-07 | 2.93 | 8.82E-04 |
| GO:0001944 | vasculature development | 38 | 5.34E-07 | 2.47 | 9.59E-04 |
| GO:0042471 | ear morphogenesis | 19 | 5.43E-07 | 4.07 | 9.74E-04 |
| GO:0051339 | regulation of lyase activity | 17 | 6.66E-07 | 4.46 | 0.0011961 |
| GO:0031279 | regulation of cyclase activity | 17 | 6.66E-07 | 4.46 | 0.0011961 |
| GO:0007389 | pattern specification process | 41 | 6.99E-07 | 2.35 | 0.0012547 |
| GO:0048666 | neuron development | 41 | 1.44E-06 | 2.28 | 0.0025795 |
| GO:0001568 | blood vessel development | 36 | 2.26E-06 | 2.40 | 0.0040512 |
| GO:0045761 | regulation of adenylate cyclase activity | 16 | 2.30E-06 | 4.34 | 0.0041255 |
| GO:0008015 | blood circulation | 22 | 3.29E-06 | 3.23 | 0.0058988 |
| GO:0003013 | circulatory system process | 22 | 3.29E-06 | 3.23 | 0.0058988 |
| GO:0008344 | adult locomotory behavior | 16 | 3.58E-06 | 4.20 | 0.0064304 |
| GO:0043583 | ear development | 21 | 4.31E-06 | 3.29 | 0.0077463 |
| GO:0042127 | regulation of cell proliferation | 61 | 4.68E-06 | 1.85 | 0.0083976 |
| GO:0007411 | axon guidance | 20 | 6.58E-06 | 3.32 | 0.0118217 |
| GO:0008016 | regulation of heart contraction | 14 | 6.79E-06 | 4.56 | 0.0121868 |
| GO:0051094 | positive regulation of developmental process | 32 | 6.92E-06 | 2.43 | 0.0124194 |
| GO:0007423 | sensory organ development | 36 | 7.39E-06 | 2.28 | 0.0132775 |
| GO:0015672 | monovalent inorganic cation transport | 40 | 8.89E-06 | 2.15 | 0.0159591 |
| GO:0051241 | negative regulation of multicellular organismal process | 20 | 8.95E-06 | 3.25 | 0.0160772 |
| GO:0031175 | neuron projection development | 32 | 1.01E-05 | 2.39 | 0.0182071 |
| GO:0001501 | skeletal system development | 38 | 1.24E-05 | 2.17 | 0.0222142 |
| GO:0000904 | cell morphogenesis involved in differentiation | 31 | 1.55E-05 | 2.38 | 0.0277662 |
| GO:0031644 | regulation of neurological system process | 21 | 1.58E-05 | 3.02 | 0.0284581 |
| GO:0051216 | cartilage development | 17 | 1.66E-05 | 3.55 | 0.0298337 |
| GO:0048667 | cell morphogenesis involved in neuron differentiation | 28 | 1.73E-05 | 2.50 | 0.0309872 |
| GO:0001505 | regulation of neurotransmitter levels | 15 | 1.77E-05 | 3.94 | 0.0317747 |
| GO:0014033 | neural crest cell differentiation | 11 | 1.82E-05 | 5.42 | 0.0327075 |
| GO:0014032 | neural crest cell development | 11 | 1.82E-05 | 5.42 | 0.0327075 |
| GO:0051240 | positive regulation of multicellular organismal process | 26 | 1.97E-05 | 2.60 | 0.0353453 |
| GO:0048839 | inner ear development | 18 | 2.10E-05 | 3.33 | 0.037628 |
| GO:0048729 | tissue morphogenesis | 33 | 2.34E-05 | 2.26 | 0.041999 |
| GO:0032989 | cellular component morphogenesis | 43 | 2.45E-05 | 1.99 | 0.0440173 |
| GO:0048812 | neuron projection morphogenesis | 27 | 2.65E-05 | 2.50 | 0.0475677 |

**Table S24. Biological functions significantly (FDR<0.05) implicated in genes that have acquired or showed increased H3K27me3 marks from 1- to 4-wk old lung by DAVID gene ontology analysis**

| **GO number** | **GO Term** | **Count** | **P-Value** | **Fold Enrichment** | **FDR** |
| --- | --- | --- | --- | --- | --- |
| GO:0045165 | cell fate commitment | 40 | 1.68E-13 | 3.89 | 3.02E-10 |
| GO:0030182 | neuron differentiation | 69 | 4.10E-12 | 2.47 | 7.37E-09 |
| GO:0001501 | skeletal system development | 55 | 1.14E-11 | 2.76 | 2.05E-08 |
| GO:0007389 | pattern specification process | 53 | 1.03E-10 | 2.67 | 1.86E-07 |
| GO:0003002 | regionalization | 44 | 2.15E-10 | 2.94 | 3.87E-07 |
| GO:0007267 | cell-cell signaling | 52 | 6.88E-10 | 2.56 | 1.24E-06 |
| GO:0048562 | embryonic organ morphogenesis | 36 | 1.16E-09 | 3.20 | 2.09E-06 |
| GO:0006928 | cell motion | 60 | 1.19E-09 | 2.34 | 2.14E-06 |
| GO:0048598 | embryonic morphogenesis | 59 | 1.36E-09 | 2.35 | 2.44E-06 |
| GO:0000904 | cell morphogenesis involved in differentiation | 41 | 6.25E-09 | 2.77 | 1.12E-05 |
| GO:0016477 | cell migration | 44 | 8.60E-09 | 2.62 | 1.55E-05 |
| GO:0048568 | embryonic organ development | 44 | 9.79E-09 | 2.61 | 1.76E-05 |
| GO:0000902 | cell morphogenesis | 51 | 1.80E-08 | 2.36 | 3.25E-05 |
| GO:0001764 | neuron migration | 21 | 3.54E-08 | 4.29 | 6.37E-05 |
| GO:0030900 | forebrain development | 34 | 4.17E-08 | 2.91 | 7.49E-05 |
| GO:0048663 | neuron fate commitment | 17 | 5.41E-08 | 5.17 | 9.74E-05 |
| GO:0032989 | cellular component morphogenesis | 54 | 7.29E-08 | 2.20 | 1.31E-04 |
| GO:0006811 | ion transport | 89 | 8.15E-08 | 1.79 | 1.46E-04 |
| GO:0051960 | regulation of nervous system development | 31 | 9.46E-08 | 3.00 | 1.70E-04 |
| GO:0007155 | cell adhesion | 74 | 1.45E-07 | 1.89 | 2.61E-04 |
| GO:0060284 | regulation of cell development | 32 | 1.47E-07 | 2.88 | 2.64E-04 |
| GO:0048666 | neuron development | 47 | 1.53E-07 | 2.30 | 2.75E-04 |
| GO:0007517 | muscle organ development | 34 | 1.54E-07 | 2.76 | 2.77E-04 |
| GO:0022610 | biological adhesion | 74 | 1.58E-07 | 1.88 | 2.85E-04 |
| GO:0006355 | regulation of transcription, DNA-dependent | 153 | 2.13E-07 | 1.49 | 3.83E-04 |
| GO:0001944 | vasculature development | 42 | 2.41E-07 | 2.40 | 4.33E-04 |
| GO:0007409 | axonogenesis | 32 | 2.63E-07 | 2.81 | 4.72E-04 |
| GO:0051252 | regulation of RNA metabolic process | 154 | 3.29E-07 | 1.48 | 5.92E-04 |
| GO:0048667 | cell morphogenesis involved in neuron differentiation | 34 | 3.48E-07 | 2.67 | 6.25E-04 |
| GO:0044057 | regulation of system process | 36 | 4.25E-07 | 2.56 | 7.65E-04 |
| GO:0048870 | cell motility | 45 | 4.57E-07 | 2.27 | 8.23E-04 |
| GO:0051674 | localization of cell | 45 | 4.57E-07 | 2.27 | 8.23E-04 |
| GO:0010628 | positive regulation of gene expression | 65 | 6.66E-07 | 1.91 | 0.001198 |
| GO:0045893 | positive regulation of transcription, DNA-dependent | 58 | 6.69E-07 | 1.99 | 0.0012028 |
| GO:0010557 | positive regulation of macromolecule biosynthetic process | 69 | 6.76E-07 | 1.86 | 0.0012152 |
| GO:0009952 | anterior/posterior pattern formation | 30 | 6.84E-07 | 2.80 | 0.0012296 |
| GO:0048706 | embryonic skeletal system development | 21 | 7.50E-07 | 3.62 | 0.0013487 |
| GO:0007411 | axon guidance | 23 | 7.86E-07 | 3.36 | 0.0014142 |
| GO:0051254 | positive regulation of RNA metabolic process | 58 | 8.47E-07 | 1.98 | 0.0015226 |
| GO:0001568 | blood vessel development | 40 | 9.24E-07 | 2.34 | 0.0016618 |
| GO:0009891 | positive regulation of biosynthetic process | 71 | 9.92E-07 | 1.82 | 0.0017845 |
| GO:0045596 | negative regulation of cell differentiation | 33 | 1.06E-06 | 2.59 | 0.0019056 |
| GO:0045941 | positive regulation of transcription | 63 | 1.18E-06 | 1.90 | 0.0021246 |
| GO:0051216 | cartilage development | 20 | 1.18E-06 | 3.67 | 0.0021284 |
| GO:0050767 | regulation of neurogenesis | 27 | 1.20E-06 | 2.93 | 0.0021528 |
| GO:0009792 | embryonic development ending in birth or egg hatching | 58 | 1.34E-06 | 1.95 | 0.0024184 |
| GO:0048858 | cell projection morphogenesis | 35 | 1.39E-06 | 2.48 | 0.0024982 |
| GO:0048812 | neuron projection morphogenesis | 32 | 1.50E-06 | 2.60 | 0.0026915 |
| GO:0045664 | regulation of neuron differentiation | 23 | 1.62E-06 | 3.23 | 0.0029057 |
| GO:0045665 | negative regulation of neuron differentiation | 13 | 1.64E-06 | 5.47 | 0.0029415 |
| GO:0006836 | neurotransmitter transport | 20 | 1.79E-06 | 3.58 | 0.0032161 |
| GO:0045944 | positive regulation of transcription from RNA polymerase II promoter | 51 | 1.86E-06 | 2.04 | 0.0033486 |
| GO:0030030 | cell projection organization | 47 | 2.00E-06 | 2.11 | 0.0035882 |
| GO:0043009 | chordate embryonic development | 57 | 2.16E-06 | 1.94 | 0.003887 |
| GO:0042127 | regulation of cell proliferation | 68 | 2.32E-06 | 1.81 | 0.004174 |
| GO:0050801 | ion homeostasis | 44 | 2.69E-06 | 2.15 | 0.0048387 |
| GO:0031328 | positive regulation of cellular biosynthetic process | 69 | 2.89E-06 | 1.79 | 0.0051986 |
| GO:0031175 | neuron projection development | 36 | 2.99E-06 | 2.36 | 0.005377 |
| GO:0045935 | positive regulation of nucleobase, nucleoside, nucleotide and nucleic acid metabolic process | 65 | 3.06E-06 | 1.82 | 0.0054986 |
| GO:0048878 | chemical homeostasis | 51 | 3.28E-06 | 2.00 | 0.005893 |
| GO:0048729 | tissue morphogenesis | 38 | 3.43E-06 | 2.28 | 0.0061744 |
| GO:0032990 | cell part morphogenesis | 35 | 4.23E-06 | 2.36 | 0.0076055 |
| GO:0030001 | metal ion transport | 58 | 4.65E-06 | 1.88 | 0.0083562 |
| GO:0007268 | synaptic transmission | 31 | 5.57E-06 | 2.49 | 0.0100207 |
| GO:0048514 | blood vessel morphogenesis | 33 | 6.83E-06 | 2.38 | 0.0122769 |
| GO:0010604 | positive regulation of macromolecule metabolic process | 75 | 7.16E-06 | 1.69 | 0.0128771 |
| GO:0051173 | positive regulation of nitrogen compound metabolic process | 65 | 8.51E-06 | 1.77 | 0.0153067 |
| GO:0001708 | cell fate specification | 16 | 8.91E-06 | 3.88 | 0.0160243 |
| GO:0006357 | regulation of transcription from RNA polymerase II promoter | 73 | 9.54E-06 | 1.70 | 0.0171483 |
| GO:0048705 | skeletal system morphogenesis | 25 | 9.84E-06 | 2.75 | 0.017701 |
| GO:0048732 | gland development | 32 | 1.64E-05 | 2.32 | 0.0294589 |
| GO:0019226 | transmission of nerve impulse | 35 | 1.73E-05 | 2.22 | 0.031165 |
| GO:0007423 | sensory organ development | 38 | 2.05E-05 | 2.11 | 0.0368055 |
| GO:0014706 | striated muscle tissue development | 24 | 2.07E-05 | 2.70 | 0.0372734 |
| GO:0043279 | response to alkaloid | 11 | 2.18E-05 | 5.24 | 0.0392093 |
